# Energy Infrastructure Clears the Way for Coyotes in Alberta's Oil Sands

**DOI:** 10.1002/ece3.71904

**Published:** 2025-08-18

**Authors:** Jamie F. Clarke, Larissa Bron, Madison Carlson, Sophia S. Labiy, Zoe Penno, Hayley Webster, Jason T. Fisher, Marissa A. Dyck

**Affiliations:** ^1^ School of Environmental Studies University of Victoria Victoria British Columbia Canada; ^2^ Department of Biology University of Victoria Victoria British Columbia Canada

**Keywords:** Alberta oil sands, camera trap, *Canis latrans*, competition, industrial disturbance, predation

## Abstract

Energy extraction and development are fragmenting the landscape in Canada's oil sands region, creating patches of boreal forest connected by millions of kilometers of cleared linear features. The impacts of oil and gas disturbance on some wildlife species, like caribou and wolves, have been a topic of much research; yet, the influence of energy development on other species, like coyotes—which have recently expanded into the boreal forest and established strong populations—is not well understood. Here, we assessed the effects of linear features on coyote distribution and interspecific interactions by deploying camera traps across multiple landscapes of varying energy disturbance intensities. Using an information theoretic approach, we tested hypotheses about the effects of linear feature type and density, natural feature coverage, and prey and competitor relative abundances on coyote monthly occurrence. High densities of wide linear features and high relative abundances of small mammal prey and large competitors best predicted coyote occurrence, whereas natural features had a negative effect. Selection for higher densities of these features suggests that wide linear clearings, like roads and geo‐survey seismic lines, provide movement paths for coyotes as they do for wolves, although they may also provide prey subsidies. Snowshoe hare and red squirrel prey, but not ungulates, had a strong positive effect on coyote occurrence, although coyote–prey relationships could shift with the hare cycle. Coyotes appeared to co‐occur with wolf and lynx competitors, perhaps through shared use of abundant resources and temporal segregation or mediated by large coyote populations—and potentially indicating a departure from top‐down coyote suppression by dominant heterospecifics. Energy development has fundamentally reshaped the boreal forest of Canada's oil sands region, giving way to landscapes that support generalist, range‐expanding species like coyotes and altering community dynamics.

## Graphical abstract

Energy development has fundamentally reshaped the boreal forest of Canada's oil sands region. Dense networks of linear features, including roads and seismic lines, have created new landscapes that support generalist, range‐expanding species like coyotes, potentially altering community dynamics.

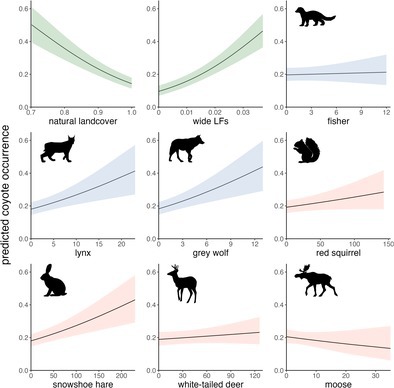

## 1 Introduction

People have profoundly changed the Earth's surface. More than 75% of the planet's ice‐free land has been anthropogenically modified (Ellis and Ramankutty 2008), affecting both climate and natural life (Steffen et al. 2005). Land use change—leading to habitat loss and fragmentation—has altered ecosystem structure and function, to the detriment of biodiversity and biological interactions (Díaz et al. 2019; Sage 2020).

An extreme example of land use change is Alberta's oil sands region: a 140,000 km^2^ stretch of boreal forest in Nearctic Canada that sits atop one of the largest hydrocarbon deposits in the world. The region has undergone intensive resource development in the last 50‐plus years, with timber harvest, road infrastructure, and energy extraction significantly and rapidly altering the landscape (Schieck et al. 2014; Dabros et al. 2017). Decades of cumulative disturbance have created a landscape without historic or global parallels (Pickell et al. 2015; Fisher and Burton 2018; Dabros and Higgins 2023).

**FIGURE 1:**
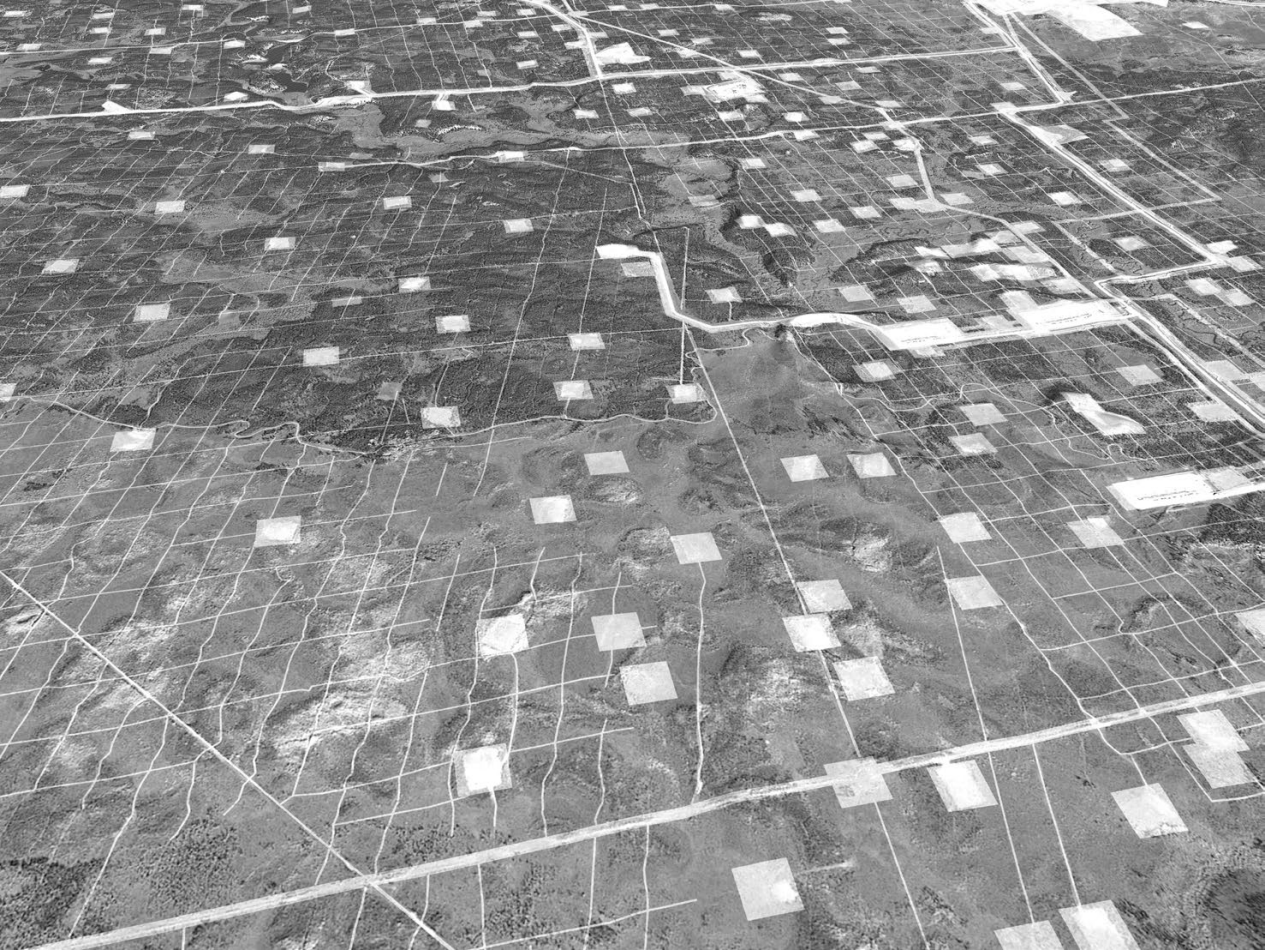
Aerial depiction of oil sands disturbances, including industrial facilities (large polygons), well pads (squares), roads (wide lines), conventional seismic lines (straight narrow lines) and 3D seismic lines (crosshatched lines). Image by Scott Heckbert for Jason T Fisher.

Particularly unique is the density of anthropogenic disturbance. The boreal forest of the oil sands region is a matrix of linear (e.g., roads) and polygonal (e.g., well pads) clearings (Figure 1). Large swaths of forest have been removed for surface mining operations, with additional mines exhausted or slated for development (Jordaan 2012). Millions more kilometers have been cleared to locate deep petroleum deposits and service in situ wells, creating novel patterns on the landscape (Timoney and Lee 2001; Jordaan 2012; Roberts et al. 2022). These clearings are mostly linear and include geo‐survey (seismic) lines, pipelines, power transmission lines, and access roads. In some parts of the oil sands region, the density of seismic lines alone was estimated to be as high as 40 km/km^2^ (Stern et al. 2018); on some leases, narrow grid‐patterned 3D seismic lines represent more than 10% of the surface footprint (Kansas et al. 2015).

Such intensive and extensive disturbance affects wildlife species variably. On the one hand, wildlife “winners” are able to capitalize on the movement and forage subsidies linear features provide (Fisher and Burton 2018; Tattersall et al. 2023). Gray wolves ( *Canis lupus* ), for example, show a preference for linear features, using them to travel farther and faster across challenging boreal terrain, potentially increasing kill rates (James and Stuart‐Smith 2000; Fryxell et al. 2007; McKenzie et al. 2012; Dickie et al. 2017). Early‐seral vegetation (e.g., grasses, forbs, and browse) planted or regrowing along linear features supports non‐native, range‐expanding white‐tailed deer, improving survival and supporting population growth (Dawe et al. 2014; Darlington et al. 2022). Wildlife “losers,” on the other hand, struggle under the pressures of forest conversion and hyperconnectivity (Fisher and Burton 2018). The mature, undisturbed forests that threatened woodland caribou ( *Rangifer tarandus caribou* ) rely on for shelter and forage have been fragmented by oil and gas development (Boutin et al. 2012; Lesmerises et al. 2013), with linear features increasing predators' access to caribou habitat (Latham, Latham, Boyce, and Boutin 2011a; Whittington et al. 2011; DeMars and Boutin 2018) and potentially shifting caribou distribution (Nellemann et al. 2001).

The impacts of energy infrastructure on another member of the oil sands community—the coyote ( *Canis latrans* )—are not as well understood. Historically, coyotes occupied the western United States and parts of south‐central Canada, primarily inhabiting grasslands, prairies and deserts, (Hody and Kays 2018). As human populations boomed, forests were converted for agriculture and wolves culled (Macdonald and Sillero‐Zubiri 2004), coyotes dramatically expanded their range, becoming the most widely distributed *Canis* species in North America (Laliberte and Ripple 2004; Hody and Kays 2018; Ward et al. 2018). Coyotes now occur in every Canadian province and territory, save Nunavut. In Alberta, coyotes expanded from their previous prairie habitat and into the boreal forest about 100 years ago, facilitated by industrial disturbance (Latham, Latham, Boyce, and Boutin 2011b). In the oil sands region, that makes coyotes a novel species on a novel landscape.

Coyotes are a critically understudied component of the oil sands ecosystem (Latham, Latham, Boyce, and Boutin 2011b). In the western boreal forest, evidence suggests coyotes prefer disturbed sites and landscapes (Barnas et al. 2024), and particularly areas of high linear feature density, potentially using them as movement corridors like wolves do (Fisher and Burton 2018; Toews et al. 2018; Tattersall et al. 2020). Yet, the kinds of linear features coyotes select for—or whether they select for them at all—is unclear. Also of interest is the interplay between energy infrastructure and coyotes' interspecific interactions. Oil and gas disturbance is known to influence community dynamics (Fisher and Ladle 2022). The increased connectivity and permeability linear features provide (Dickie et al. 2017), coupled with coyotes' recent expansion into and success in the region (Burgar et al. 2019), could have important consequences on other species' behavior and distribution (Heim et al. 2017; Lendrum et al. 2018; Mumma et al. 2019; Chow‐Fraser et al. 2022; Fisher and Ladle 2022; Boczulak et al. 2023).

Diet studies show that coyotes mostly consume small mammals, ungulates, vegetation, and anthropogenic “by‐catch” like pets and livestock (Todd et al. 1981; Lukasik and Alexander 2012; Shi et al. 2021; Jensen et al. 2022; Hayward et al. 2023). Analyses of coyote scat from Alberta indicate that snowshoe hares, rodents, and ungulates are some of the most important food sources for coyotes in the region (Todd et al. 1981; Murray et al. 2015). Although deer fawns and moose calves are coyotes' preferred ungulate prey, there have also been reports of “spill‐over” predation on caribou in the eastern boreal forest (Boisjoly et al. 2010; Latham, Latham, Boyce, and Boutin 2011b) and coyotes prey on caribou calves in Atlantic Canada, where wolves are absent (Mahoney et al. 2016). Whether those ungulates are preyed on or scavenged is typically unclear, however, unless directly observed.

Interspecific competition is challenging to quantify (Murray et al. 2023), but there is some evidence of interference and exploitative competition between coyotes and felids, mustelids, and other canids. Coyotes share prey species—including snowshoe hares—with lynx, for example (Ruggiero 1994; Krebs, Boutin, and Boonstra 2001). Coyotes and cougars also minimize spatial and temporal overlap to reduce conflict (Jensen et al. 2024). Linear features increase competition between coyotes and wolverines (Chow‐Fraser et al. 2022), whereas fishers and coyotes both preferentially prey on small mammals (Weir et al. 2005). Similarly, coyotes appear to be limited by competition with gray wolves (Berger and Gese 2007).

To better understand how linear features influence coyote distribution and behavior, we measured monthly coyote occurrence in the western boreal forest, across six landscapes with different degrees of energy development, using camera traps (O'Connell et al. 2011; Burton et al. 2015). Using generalized linear models informed by these data, we employed an information‐theoretic approach, which weighed evidence for additive and interactive models representing several competing hypotheses, sensu Burnham and Anderson (2002). We hypothesized that coyote occurrence would (1) increase with linear feature density; (2) increase with increasing relative abundance of herbivore prey species; and (3) decrease with increasing relative abundance of competitor species. We predicted that coyote occurrence would increase with linear feature density since they provide movement subsidies for canids (Dickie et al. 2017) and forage subsidies for herbivore prey (Finnegan et al. 2019; Wittische et al. 2021; Darlington et al. 2022). We further predicted that coyote occurrence would increase with higher relative abundance of prey species and decrease with higher relative abundance of competitor species, as coyotes would frequent prey‐rich areas but reduce spatial overlap with competitors (Ballard et al. 2003).

## 2 Methods

### 2.1 Study Area

**FIGURE 2:**
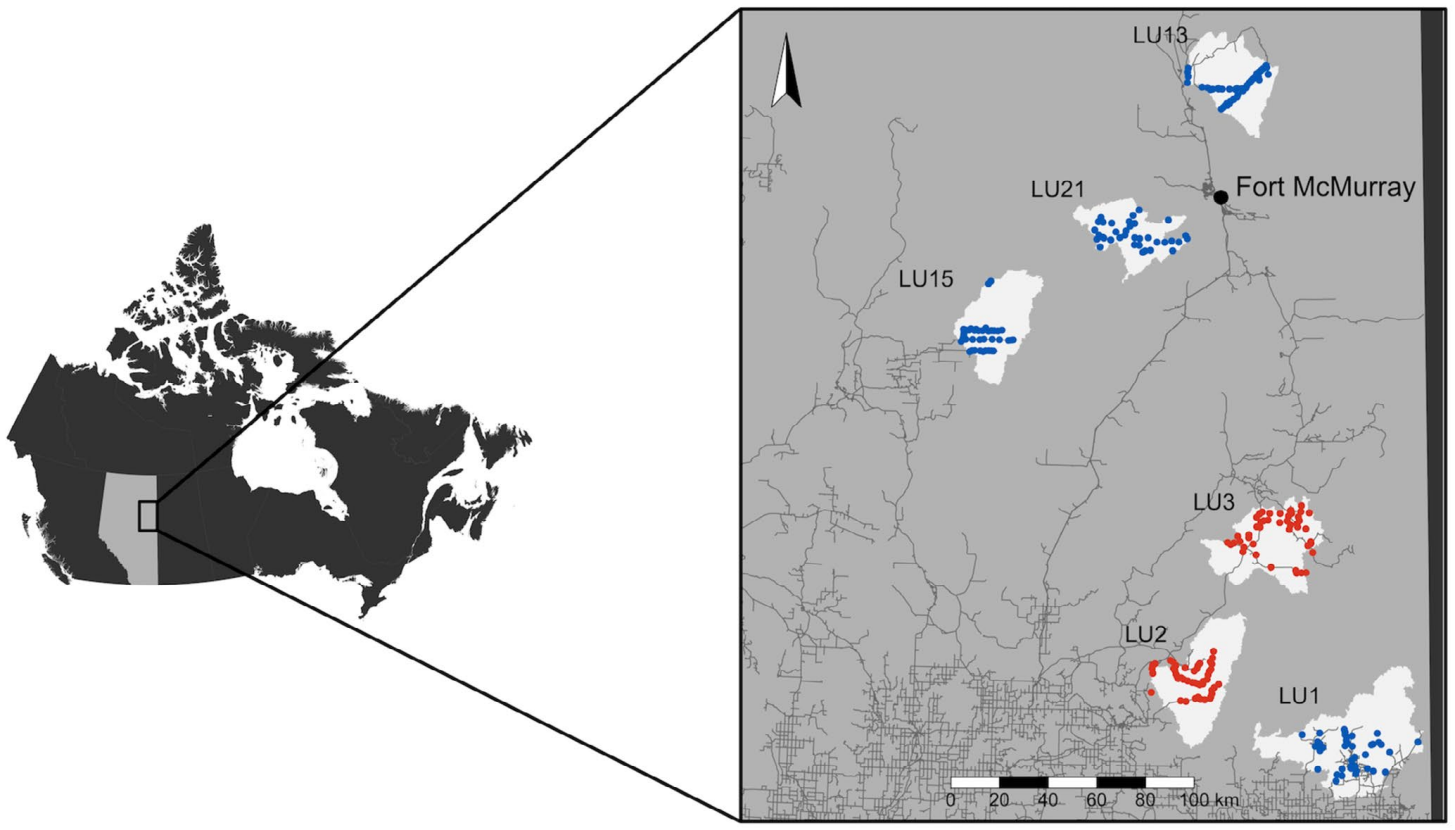
Map of camera trap deployments across six LUs (light polygons) in the oil sands region of Alberta. Left: Map of Canada, with Alberta in light gray and the study area enclosed in the black box. Right: Red dots represent cameras active 2021–2022 (*n* = 78); blue dots represent cameras active 2022–2023 (*n* = 155). Gray lines show road networks, including unpaved roads.

Our study frame was the vast portion of the boreal forest known as the western sedimentary basin (Porter et al. 1982); within this frame, our study extent was Canada's oil sands region, in northeastern Alberta (Figure 2). The region's topography is flat‐to‐undulating and is composed of upland forests—filled with white pine ( *Picea glauca* ), black spruce ( *Picea mariana* ), trembling aspen ( *Populus tremuloides* ), and jack pine ( *Pinus banksiana* ) – and Labrador tea‐ ( *Rhododendron groenlandicum* ) dominated lowland muskegs. Winters in the oil teas region are typically long and cold, and summers short and warm; in the coldest month, the mean temperature is about −19°C, and in the hottest month, 16°C, with about 45the 0 mm of annual precipitation (Downing and Pettapiece 2006). The boreal forest supports a diversity of mammal species, including wolves, coyotes, lynx ( *Lynx canadensis* ), red foxes ( *Vulpes vulpes* ), black bear ( *Ursus americanus* ), fishers ( *Pekania pennanti* ), wolverines ( *Gulo gulo* ), martens ( *Martes americana* ), woodland caribou, moose ( *Alces alces* ), and white‐tailed deer ( *Odocoileus virginianus* ).

This research was conducted under the joint Canada‐Alberta Oil Sands Monitoring program (Roberts et al. 2022). The study design for this program divides the region into ~1000 km^2^ landscape units (LUs) on the basis of hydrological boundaries, with each representing a differing degree of cumulative development from forestry, road building, and oil and gas exploration and extraction (Bayne et al. 2021). LUs were characterized as (1) currently developed for in situ or mine extraction, (2) proposed for in situ development or mine site, or (3) low disturbance reference site (Bayne et al. 2021). A subset of LUs representing all three disturbance levels was selected for this project.

### 2.2 Sampling Design

A total of 233 Reconyx Hyperfire 2X camera traps (Reconyx, Homen, Wisconsin) were deployed in six LUs (Figure 2, Table 1): one mine site, one proposed in situ site, two active in situ sites, and two low‐disturbance sites. Camera traps were deployed using a constrained stratified sampling design. Each LU was divided into 60 2‐km^2^ hexagonal cells using ArcGIS (version 10.3); cells were then categorized as upland (> 50% deciduous) or lowland (> 50% wet coniferous) forest. About 40 cells (actual range: 36–42) were selected for camera trap deployment, with forest types represented roughly equally. We chose a constrained stratified design to control for natural variability, to tease out the effects of industrial development on mammal communities. 78 cameras were set across two LUs from October 2021–2022, and 155 were set across four LUs from fall 2022–2023, for a maximum duration of 13 months per camera trap.

**TABLE 1:** LU numbers, descriptions and sampling information.

LU	Classification	Year sampled	Number of cameras
1	In situ extraction	2022–2023	39
2	Low disturbance	2021–2022	42
3	In situ extraction	2021–2022	36
13	Mine site	2022–2023	41
15	Pre in situ extraction	2022–2023	39
21	Low disturbance	2022–2023	36

In each selected cell, a camera was deployed at least 100 m from roads or trails and 1 km from camera stations in adjacent cells. Cameras were set about 0.5 m off the ground and pointing down a defined wildlife trail (i.e., a clear, well‐trodden path in the forest, not human‐made). Ca. 40 mL of scent lure (Long Distance Call, O'Gorman's, Montana) was spread on a tree within each camera's viewshed. Our design maximizes site accessibility and the probability of medium‐to‐large mammal detections while maintaining site independence (Diniz‐Filho et al. 2003; Hawkins et al. 2007). Cameras were set to take one image per motion sensor trigger to prolong battery life and storage capabilities. Sensitivity was set to high, with no quiet period between photos, and a daily timelapse image was captured at noon to confirm camera trap operability.

### 2.3 Defining Variables

To quantify natural boreal heterogeneity, landscape data were derived from the Alberta Biodiversity Monitoring Institute's wall‐to‐wall landcover map (Alberta Biodiversity Monitoring Institute 2010, 2021, 2024). Landscape and industrial features within a 4750 m radius of each camera station were considered, since 4750 m was the top‐performing buffer distance for coyotes in anthropogenically disturbed areas in the oil sands region (Dyck et al., In preparation). To avoid overparameterization in our models, and given that coyotes are habitat generalists, we grouped natural landcover variables (grasslands, shrubland, and coniferous/broadleaf/mixed forest) into a single covariate (Mastro et al. 2019; Petroelje et al. 2021). Linear features considered included pipelines, transmission lines, roads, conventional seismic lines, 3D seismic lines, and trails (Table S1). All landscape and industrial features were measured as proportions.

Image data were processed by trained technicians using Timelapse2.0 software (Greenberg and Godin 2012). Images of a single species captured within a 30‐min window at the same site were grouped into independent detections. For example, at a given camera site, an image sequence of a coyote captured at 10:00 and an image sequence of a coyote captured at 10:28 were considered a single independent detection event. Independent detections of snowshoe hares, red squirrels, white‐tailed deer, caribou, and moose were considered for prey species; independent detections of lynx, cougars, wolverines, fishers, and wolves were considered for competitor species. We interpreted total independent detections as relative abundances—that is, indices of abundance relative to other species.

Coyote detections were used to calculate the monthly occurrence (hereafter, occurrence) of coyotes at every camera site. Only cameras with ≥ 15 operational days per month were used to calculate coyote occurrence, to account for occasional camera failures (Fisher and Ladle 2022). The response metric was thus the number of months a coyote was detected, and the number of months a coyote was not detected, to inform a proportional binomial model wherein each month is a Bernoulli trial (Crawley 2012; Faraway 2016). Here, we considered a zero as a true zero and not partitioned as error into *p* (probability of detection) as is the case with occupancy models (MacKenzie et al. 2002): if we did not detect a coyote in a month on a lured wildlife trail, we were confident of its absence. Such an assumption could induce false absences (e.g., a coyote passed too quickly through a camera trap's viewshed to be captured in an image, resulting in no detection despite presence) and potentially lead to false zeroes for monthly occurrence, but given our attempts to maximize detections, we considered it a reasonable assumption. Moreover, the probability of false absence (1‐*p*) compounds over multiple surveys to near zero even for rarely detected species (Fisher et al. 2014), let alone a common animal over 12–13 surveys.

### 2.4 Model Framework

We carried out a two‐step model selection to (1) explore which linear features to include in analyses and (2) test our hypotheses on coyote occurrence. Before modeling, we tested multicollinearity between covariates using pairwise Pearson and Spearman's correlation tests. Covariates with a correlation coefficient (r) ≥ 0.6 were not included in the same models. Only pipelines were correlated with other covariates (transmission lines, roads, and 3D seismic lines; Script S1).

Models were constructed using the *lme4* package (Bates et al. 2015) and ranked using the Akaike Information Criterion corrected for small sample size (AICc; Akaike 1973; Sugiura 1978) using the *MuMIn* package (Bartoń 2023) in R (version 4.3.2). Models were fit using maximum likelihood. The best‐supported models had the lowest AICc scores by ∆ AICc of ≥ 2 (Burnham and Anderson 2002). Variables were scaled for standardized comparison between estimated model coefficients, with a mean of 0 and a standard deviation of 1. Beta coefficients (*β*) are reported as estimate ± standard error, with associated *p* value. For our best‐fit model in each step, we calculated variance inflation factors (VIFs) using the package *car* (Fox et al. 2019) to test for collinearity between covariates. VIFs report how much of a given covariate's variability is explained by other covariates, owing to correlation (Craney and Surles 2002). A VIF value of 1 indicates no correlation, with larger values (e.g., > 5) signaling severe correlation.

#### 2.4.1 Step 1—Linear Feature Groupings

In step 1, we competed different groupings of linear features—narrow (~5 m across; 3D seismic lines and trails), wide (> 5 m across; roads, conventional seismic lines and roads), vegetated (not paved or graveled; conventional seismic lines, 3D seismic lines, transmission lines, and trails), unvegetated (paved or graveled; roads), pipelines (modeled individually given correlation with other linear features) and global linear features (all uncorrelated linear features). We binned linear features into groups to limit interaction terms in step 2, which could complicate model selection and interpretation. We used a generalized linear mixed model (GLMM) framework with a binomial distribution and set LU as a random effect, such that, for the global linear features model:

$$\begin{array}{c} \eta_{\mathrm{ij}}=\beta_{0}+\beta_{1}{\text{Roads}}_{\mathrm{ij}}+\beta_{2}{\text{Seismic}}_{\mathrm{ij}}\\ \;+\beta_{3}3{\text{DSeismic}}_{\mathrm{ij}}+\beta_{4}{\text{Transmission}}_{\mathrm{ij}}\\ \;+\beta_{5}{\text{Trails}}_{\mathrm{ij}}+{\mathrm{LU}}_{\mathrm{ij}} \end{array}$$

$$\text{logit}\left(\theta_{\mathrm{ij}}\right)=\eta_{\mathrm{ij}}$$

$$\text{monthly coyote occurrence}\sim \text{Bernoulli}\left(\theta_{\mathrm{ij}}\right)$$

$${\mathrm{LU}}_{\mathrm{ij}}\sim \text{Normal}\left(0,\sigma^{2}\right)$$

where $\eta_{\mathrm{ij}}$ is the linear predictor at the i th observation and j th level random effect; $\beta_{0}$ is the intercept, $\beta_{n}X_{\mathrm{ij}}$ is a covariate of interest; and ${\mathrm{LU}}_{\mathrm{ij}}$ is the random effect. The link function was used to predict the effect of covariates on coyote occurrence. Coyote occurrence was assumed to follow a Bernoulli distribution, where each month was considered an independent trial and coyotes were either detected (1) or not (0), with each camera site considered a unique replication (Fisher and Ladle 2022). We included LU as a random effect to account for inherent variability between landscapes, since models that included random effects outperformed fixed effect‐only models (Script S2). LU also served as a partial proxy for sampling year, as each LU was sampled for a single year.

#### 2.4.2 Step 2—Landscape Features, Prey and Competitors

In step 2, we tested the effects of proportional coverage of landscape features (the best‐performing linear feature grouping and natural landcover) and independent prey and competitor detections on coyote occurrence (Figure S1). Again, we used a GLMM with a binomial distribution and set LU as a random effect, such that for the global model:

$$\begin{array}{c} \eta_{\mathrm{ij}}=\beta_{0}+\beta_{1}{\text{Natural}}_{\mathrm{ij}}+\beta_{2}{\text{Linear Feature}}_{\mathrm{ij}}+\beta_{3}{\text{Deer}}_{\mathrm{ij}}\\ \;+\beta_{4}{\text{Moose}}_{\mathrm{ij}}+\beta_{5}{\text{Squirrel}}_{\mathrm{ij}}+\beta_{6}{\text{Hare}}_{\mathrm{ij}}+\beta_{7}{\text{Wolf}}_{\mathrm{ij}}\\ \;+\beta_{8}{\text{Lynx}}_{\mathrm{ij}}+\beta_{9}{\text{Fisher}}_{\mathrm{ij}}+{\mathrm{LU}}_{\mathrm{ij}} \end{array}$$

$$\text{logit}\left(\theta_{\mathrm{ij}}\right)=\eta_{\mathrm{ij}}$$

$$\text{monthly coyote occurrence}\sim \text{Bernoulli}\left(\theta_{\mathrm{ij}}\right)$$

$${\mathrm{LU}}_{\mathrm{ij}}\sim \text{Normal}\left(0,\sigma^{2}\right)$$

We also competed models with interaction terms between prey or competitor species and linear features. For interaction models, we chose the prey and competitor species we believed exerted the most influence on coyote occurrence, given previous findings. We selected snowshoe hares as the main prey species, as lagomorphs are a key component of the coyote diet (Prugh 2005; Shi et al. 2021; Hayward et al. 2023), especially in their boreal range (Todd et al. 1981) where they overlap with wolves (Petroelje et al. 2021). Likewise, we selected wolves as the main competitor species, since they are competitively dominant to coyotes (Merkle et al. 2009), harassing and occasionally killing coyotes in areas of high wolf use and density (Miller et al. 2012; Flagel et al. 2017).

## 3 Results

### 3.1 Mammal Detections

We captured a total of 208,655 camera trap images (excluding blanks) over the course of the study. Of those, 178,730 images were of mammals identifiable to the species level (excluding people and domestic dogs).

Camera images generated 15,944 total independent detections of 10 focal species over a cumulative total of 82,027 camera trap days (Figure S2). The most‐detected species was white‐tailed deer (6143 independent detections), followed by snowshoe hare (4572), red squirrel (2200), coyote (1319), moose (696), lynx (526), fisher (262) and gray wolf (226). Caribou, cougars, and wolverines had too few detections (115, 37, and 8, respectively) to carry forward into analyses. Coyotes were detected at 172 of 233 sampling sites (74%). Mean naive occupancy (number of months present divided by total number of months sampled at each camera site, averaged across all camera sites) for coyotes was 0.24 (Figure S3).

### 3.2 Coyote Occurrence Increases With Road and Seismic Line Densities

Higher densities of wide linear features—which included roads, conventional seismic lines, and transmission lines—best explained coyote occurrence when compared with other linear feature groupings. In step1, the wide linear feature model—which grouped linear features > 5 m in width—was top performing (AICc = 980.4, ∆ AICc = 0, weight = 0.58; Table 2). Of the three wide linear feature types, roads had the strongest positive effect on coyote occurrence (*β* = 0.59 ± 0.06, *p* < 0.001), with conventional seismic lines also having a positive—but slightly smaller—effect size (*β* = 0.18 ± 0.07, *p* = 0.01). Transmission lines had a negligible effect on coyote occurrence (*β* = 0.01 ± 0.06, *p* = 0.82). Odds of coyote occurrence increased markedly with scaled proportions of roads and conventional seismic lines (Figure S4). VIF values for all top model covariates were near 1 (range: 1.06–1.13), suggesting minimal collinearity. The global linear feature model, which included all linear features save for pipelines, was the second‐most supported (AICc = 982.5, ∆ AICc = 2.04, weight = 0.21), followed by the road‐only unvegetated linear feature model (AICc = 982.5, ∆ AICc = 2.05, weight = 0.21). The difference in AICc scores between the wide and global linear features models was > 2, lending support for the top model (Burnham and Anderson 2002; Burnham et al. 2011; Table S2).

**TABLE 2:** Step 1: GLMMs predicting monthly coyote occurrence given linear feature types.

Model	Covariates	df	logLik	AICc	∆ AICc	AICc weight
Wide linear features	Roads + seismic lines + transmission lines	5	−485.1	980.4	0	0.58
Global linear features	Roads + seismic lines +3D seismic lines + trails + transmission lines	7	−484.0	982.5	2.04	0.21
Unvegetated linear features	Roads	3	−488.2	982.5	2.05	0.21
Vegetated linear features	Seismic lines +3D seismic lines+ trails + transmission lines	6	−527.8	1068.0	87.53	0
Narrow linear features	3D seismic lines + trails	4	−533.2	1074.5	94.09	0
Pipelines	Pipelines	3	−534.3	1074.8	94.34	0
Null	—	2	−537.3	1078.6	98.13	0

*Note:* Models are ranked from most‐to‐least supported. Model statistics include degrees of freedom (df), log likelihood (logLik), Akaike information criterion score corrected for small sample size (AICc), difference in AICc score from the best‐supported model (∆ AICc) and explanatory value of each model (AICc weight).

### 3.3 Wide Linear Features, Prey, and Competitors Positively Influence Coyote Occurrence

Coyote occurrence increased with wide linear feature density, and small mammal and large competitor relative abundance. In step 2: the global model, which included terms for natural landcover, wide linear features, and prey and competitor species, best predicted coyote occurrence (AICc = 921.9, ∆ AICc = 2.09, weight = 0.74; Table 3). The global model with interaction terms was the second‐best performing (AICc = 924.0, ∆ AICc = 2.09, weight = 0.26; Table S3). Given the difference in AICc scores between the first‐ and second‐ranked models was > 2, the top‐performing global model was supported.

**TABLE 3:** Step 2: GLMMs predicting monthly coyote occurrence given proportion of landcover and wide linear features, and total detections of prey and competitor species.

Model	Covariates	df	logLik	AICc	∆ AICc	AICc weight
Global	Natural landcover + wide linear features + red squirrel + snowshoe hare + white‐tailed deer + moose + fisher + lynx + gray wolf	11	−449.3	921.9	0	0.74
Global interaction	Natural landcover + red squirrel + white‐tailed deer + moose + fisher + lynx + wide linear features*snowshoe hare + wide linear features*gray wolf	13	−448.2	924	2.09	0.26
Competitor species, natural landcover, and wide linear features	Natural landcover + wide linear features + fisher + lynx + gray wolf	7	−461.3	937.1	15.25	0
Global competitor interaction	Natural landcover + fisher + lynx + wide linear features*gray wolf	8	−460.7	938.1	16.20	0
Global prey interaction	Natural landcover + red squirrel + white‐tailed deer + moose + wide linear features*snowshoe hare	9	−461.0	940.8	18.95	0
Prey species, natural landcover and wide linear features	Natural landcover + wide linear features + red squirrel + snowshoe hare + white‐tailed deer + moose	8	−146.2	941	19.12	0
Natural landcover and wide linear features	Natural landcover + wide linear features	4	−479.0	966.2	44.34	0
Prey species and natural landcover	Natural landcover + red squirrel + snowshoe hare + white‐tailed deer + moose	7	−480.9	976.2	54.33	0
Competitor species and natural landcover	Natural landcover + fisher + lynx + gray wolf	6	−489.1	990.5	68.62	0
Prey species and wide linear features	Wide linear features + red squirrel + snowshoe hare + white‐tailed deer + moose	7	−489.9	994.3	72.44	0
Prey interaction	Red squirrel + white‐tailed deer + moose + wide linear features*snowshoe hare	8	−489.4	995.5	73.61	0
Competitor species and wide linear features	Wide linear features + fisher + lynx + gray wolf	6	−492.3	996.9	75.01	0
Competitor interaction	Fisher + lynx + wide linear features*gray wolf	7	−491.7	997.8	75.91	0
Natural landcover	Natural landcover	3	−503.7	1013.6	91.69	0
Prey species	Red squirrel + snowshoe hare + white‐tailed deer + moose	6	−512.2	1036.8	114.89	0
Competitor species	Fisher + lynx + gray wolf	5	−522.6	1055.5	133.63	0
Null	—	2	−537.3	1078.6	156.69	0

*Note:* Models are ranked from most to least supported. Model statistics include degrees of freedom (df), log likelihood (logLik), Akaike information criterion score corrected for small sample size (AICc), difference in AICc score from the best‐supported model (∆ AICc) and explanatory value of each model (AICc weight). Note: For interaction models, only the interaction term and not the lower‐level terms were included (i.e., y ~ A*B and not y ~ A + B + A*B).

**FIGURE 3:**
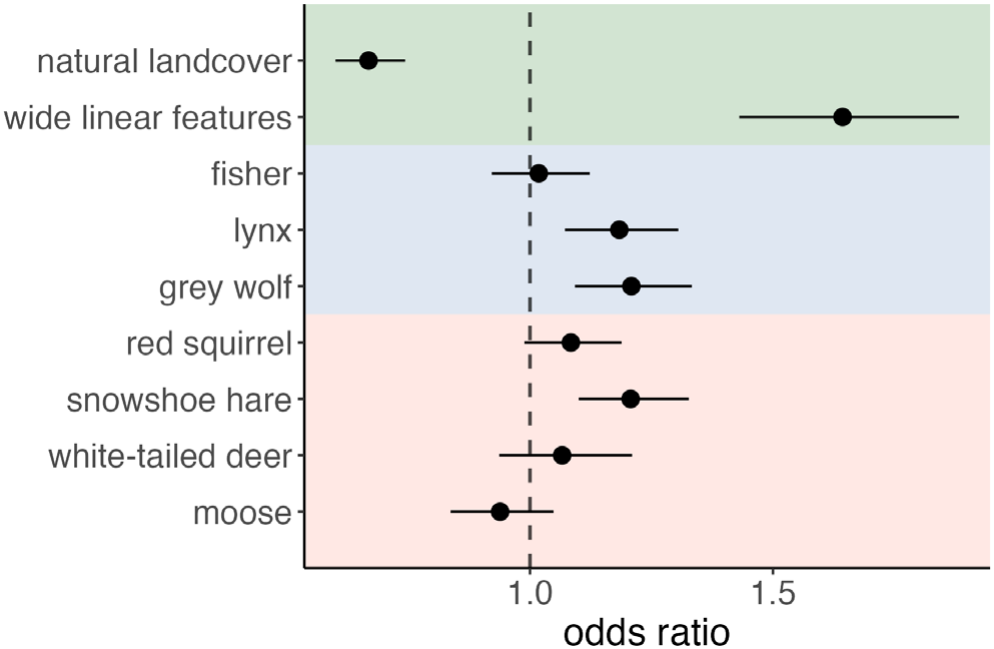
Odds ratio plot for the top‐performing global model showing the effects of proportional coverage of natural landcover and wide linear features (green box), and of total independent competitor (blue box) and prey (red box) species detections, on monthly coyote occurrence. Black dots represent exponentiated model coefficients; bars represent 95% confidence intervals. The dashed line represents an odds ratio of 1.

**FIGURE 4:**
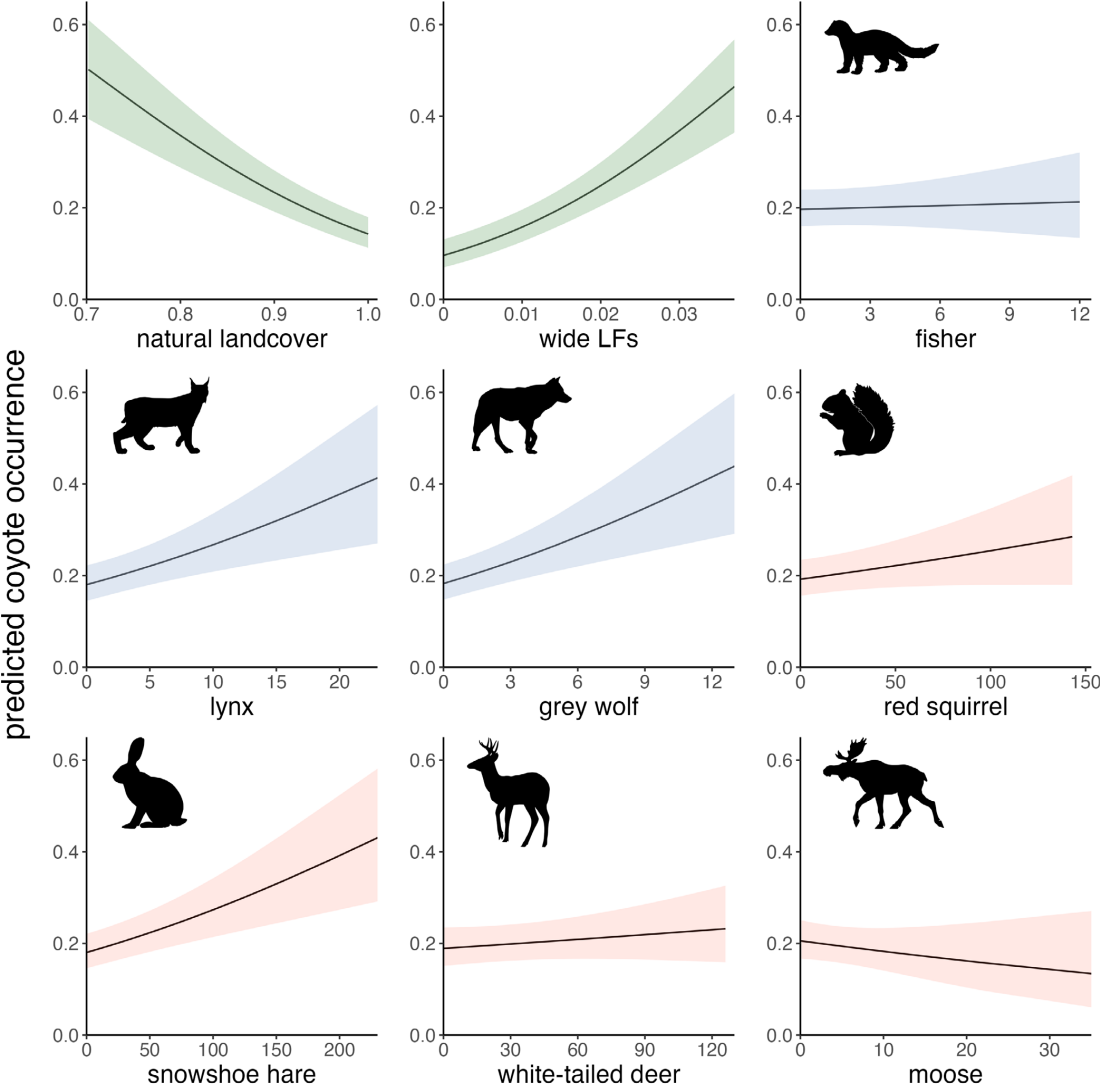
Proportions of natural landcover and wide linear features near camera trap sites, and total independent detections of prey and competitor species, influence predicted monthly coyote occurrence. Black lines represent the predicted relationship between coyote occurrence and covariates; shaded colors represent 95% confidence intervals (landscape features in green, competitors in blue and prey in red). LFs is shorthand for linear features.

The proportion of wide linear features on the landscape retained a strong positive effect on coyote occurrence (*β* = 0.50 ± 0.07, *p* < 0.001), additive to detections of small mammal prey like snowshoe hares (*β* = 0.19 ± 0.05, *p* < 0.001) and red squirrels (*β* = 0.08 ± 0.05, *p* = 0.08), and competitors like gray wolves (*β* = 0.19 ± 0.05, *p* < 0.001) and lynx (*β* = 0.17 ± 0.05, *p* < 0.001; Figures 3 and 4). Total white‐tailed deer detections also had a positive relationship with coyote occurrence, but the effective size was smaller (white‐tailed deer: *β* = 0.06 ± 0.07, p = 0.33). The area of grouped natural landcover was the only covariate to have a clear negative effect on coyote occurrence (*β* = −0.40 ± 0.05, *p* < 0.001). Total moose detections had a slight negative relationship, but again, the effect size was small (*β* = −0.06 ± 0.06 *p* = 0.27). Fishers had a negligible influence on coyote occurrence (*β* = 0.02 ± 0.05, *p* = 0.72). VIF values for all top model covariates were near 1, indicating little collinearity (range: 1.04–1.31; Figure S5). Simulation from the top model showed high precision in fixed effect estimates (Figure S6), and high certainty in top model selection (Figure S7).

## 4 Discussion

Anthropogenic landscape change facilitates species range expansions globally (Laliberte and Ripple 2004; Essl et al. 2019; Forgione et al. 2022). Contemporary range expansions are often extensions of longer‐term, larger‐scale patterns, and we propose the same for coyote movement into the western boreal forest (Hody and Kays 2018). Our research suggests that wide oil and gas linear features are clearing the way for range‐expanding coyotes in Alberta's oil sands. Across multiple landscapes spanning a gradient of disturbance, coyote occurrence was greater in places of high wide linear feature density and lower in natural landscapes. Contrary to expectations, coyotes co‐occurred with competitors in space, especially where prey relative abundance was high.

### 4.1 Road and Seismic Line Networks Provide Paths for Coyotes

Wide linear features—especially roads and seismic lines—had the largest positive effect on coyote occurrence, whereas the proportion of natural landcover had the largest negative effect. Compared to dense boreal forest (Crête et al. 2001) and even natural corridors (Newton et al. 2017), linear features likely represent the least‐cost path for coyotes (Sawyer et al. 2011). Coyotes may show a preference for wide linear features because these clearings are long, straight, and unobstructed, maximizing travel speed, distance covered, and line‐of‐sight for movement and hunting compared to narrow linear features or forest patches (Dickie et al. 2017). Our findings are in line with other multispecies studies from the oil sands region broadly linking predators and wide linear features (Skatter et al. 2020; Beirne et al. 2021), particularly roads (Fisher and Burton 2018) where prey are present (Fisher and Ladle 2022). Many roads within the oil sands region are low‐traffic access routes, with less road‐use risk (Van Scoyoc et al. 2024) to weigh against the benefits of roadside hunting and movement.

Importantly, the wide linear feature model (which included roads, seismic lines and transmission lines) outperformed the road‐only unvegetated linear feature model, indicating seismic lines' importance (the effect of transmission lines was negligible). Roads had the strongest predictive effect on coyote occurrence, but seismic lines had the second‐strongest, suggesting they could provide alternate or additive pathways to roads—as roads can also be risky for coyotes, bringing wildlife into closer contact with people and increasing chances of vehicle strikes (reviewed in Coffin 2007). Coyotes may also exploit seismic lines simply because they are more pervasive than roads. Within our study LUs and site buffer radius, seismic lines had a mean proportional coverage of 0.007 (0.7%) compared to 0.004 (0.4%) for roads; moreover, seismic lines were represented within‐buffer‐radius for 99% of camera sites versus 87% for roads. Coyotes may therefore select seismic lines as a “second choice” linear feature because they are structurally similar (e.g., unobstructed, straight) and numerous.

### 4.2 A Case for Competitor Coexistence and Dominance Re‐Structuring

Contrary to our last hypothesis, coyotes co‐occurred with competitor species—particularly gray wolves and lynx. One potential explanation is that coyotes aggregate near competitors to feed on their kills (Paquet 1992; Wilmers et al. 2003). Coyotes are facultative scavengers (Walker et al. 2021) that have been shown to consume more carcasses when wolves are on the landscape (Switalski 2003; Atwood and Gese 2008) and form foraging associations with other mammals (Thornton et al. 2018). The strong predictive signal of wolf detections on coyote occurrence, coupled with the knowledge that linear features may improve predation rates for wolves (Messier and Crête 1985; Fryxell et al. 2007; Dickie et al. 2017), could indicate a higher number of wolf kills and subsequently more scavenging by coyotes.

Outcomes of wolf–coyote interactions also depend on pack size. Wolves are considered the dominant of the two canids (Levi and Wilmers 2012), with many documented cases of wolves killing coyotes in direct competition (see Mech and Boitani 2019). They can, however, be overrun by coyotes when wolf packs are small (i.e., one or two individuals; Merkle et al. 2009). Wolves in Alberta's boreal forest have been culled in several landscapes to support caribou recovery (Hervieux et al. 2014; Marris 2015), potentially reducing wolf pack size and altering pack cohesion and persistence (Borg et al. 2015; Cassidy et al. 2023; Grente et al. 2024). Fracturing packs and reducing pack size could make wolves vulnerable to displacement by coyotes, potentially altering dominance structure. Taken together, our results suggest that wolves may not be suppressing coyotes in Alberta's oil sands region, as they do in other systems (e.g., Krefting 1969; Smith et al. 2003; Berger and Conner 2008; Levi and Wilmers 2012).

Snowshoe hare detections had a clear, positive influence on coyote occurrence, further suggesting that hares are an important prey species for coyotes in the boreal (Todd et al. 1981; O'Donoghue et al. 1998), and indicating that lynx and coyote (and wolf) diets are likely to overlap in the oil sands region—especially at disturbed sites (Fisher et al. 2021) and when hares are abundant. Indeed, evidence from the oil sands region places the hare cycle near its 10‐year peak during our sampling period (Skatter et al. 2020). Estimates of peak hare density in the Yukon and Alaska have been as high as 300–1000 hares/km^2^ (Ward and Krebs 1985; Slough and Mowat 1996; Krebs, Boonstra, et al. 2001); given that lynx eat roughly two snowshoe hares every 3 days when they are plentiful (Government of Northwest Territories), if hare densities were similar to peak northern estimates during our camera trap study, competition for food resources between the two species may have been minimal and spatial overlap high.

### 4.3 Knowledge Gaps

Still unknown in this system are coyotes' impacts on endangered caribou. Spill‐over predation, facilitated by hyper‐connective linear features (Mumma et al. 2018) and spurred by large coyote populations, has been posited in the oil sands region. Indeed, facultative, disturbance‐mediated coyote‐caribou predation is likely in an eastern boreal ecosystem (Boisjoly et al. 2010) and caribou comprise an important part of coyote diets in other provinces (see Huang et al. 2021). Further investigation into patterns of coyote predation, especially at lows in the hare cycle, could elucidate the relationship between coyotes and endangered caribou and clarify management priorities.

Future research could also investigate the nuances of coyote linear feature use throughout the year. Wolves have been shown to exert seasonal preferences for linear feature type (Dickie et al. 2017) and weaker selection for linear features during the wintertime (Latham, Latham, Boyce, and Boutin 2011a); similar patterns for coyotes may have been masked by the coarseness of our analyses. Coyotes' overlap with prey species could likewise change seasonally. Other canids consume prey of different sizes during alternating seasons, for example (Latham, Latham, McCutchen, and Boutin 2011). Ungulates could therefore be a better predictor of coyote occurrence at finer time scales, including during calving, when they are more vulnerable to coyote attacks.

We analyzed the effects of wide linear features on coyote distribution and behavior, given that the wide linear feature model was best supported in step 1. Our approach streamlines model selection but ignores the potentially ecologically meaningful influences of other linear feature types. Future research could explore how different linear features interact with coyote prey and competitor relative abundances.

### 4.4 A Thousand‐Foot View

Alberta's oil sands region has changed drastically under the compounding pressures of industry. Linear features created for forestry and energy extraction have fragmented the boreal forest, creating matrices of treed patches connected by networks of linear clearings (Pattison et al. 2016). What was previously good habitat for moose, caribou, and lynx has given way to landscapes that support generalist, range‐expanding species like coyotes and white‐tailed deer, fundamentally changing the distributions and relative abundances of mammal populations (Fisher and Burton 2018).

Linear features have outsized effects on wildlife ecology (e.g., Trombulak and Frissell 2000; Whittington et al. 2005; Ibisch et al. 2016). In the oil sands, where linear feature density is high (Komers and Stanojevic 2013; Stern et al. 2018), that effect is even more pronounced, influencing mammals and birds (Lankau et al. 2013; Fisher and Burton 2018; Darling et al. 2019), vegetation community composition and regeneration (van Rensen et al. 2015; Dabros et al. 2017), and interspecific relationships (Heim et al. 2017; Lendrum et al. 2018; Mumma et al. 2019; Chow‐Fraser et al. 2022; Fisher and Ladle 2022; Boczulak et al. 2023). Indeed, anthropogenic disturbance is believed to have a bigger role (*β* coefficient effect size) in the spatial distribution of mammals than natural ecological processes in Alberta's oil sands (Fisher and Ladle 2022). With thousands of kilometers of new linear features cleared each year (Komers and Stanojevic 2013), a holistic understanding of wildlife linear feature use is therefore critical for impactful management and conservation (Latham, Latham, Boyce, and Boutin 2011a; Newton et al. 2017; Finnegan et al. 2023; Benoit‐Pépin et al. 2024).

Alberta's oil sands are the harbinger of a new hydrocarbon age. As global sources of conventional oil are depleted (Bentley 2002), international interest is shifting towards “unconventional” oil sands deposits, which sit beneath thousands of square kilometers of forest (Rosa et al. 2017) inhabited by diverse fauna. Regions considering oil sands development should look to Canada to better understand how industrial infrastructure influences wildlife species and interactions, and weigh the ecological costs of energy extraction and habitat restoration with potential economic benefits.

## Author Contributions

**Jamie F. Clarke:** conceptualization (equal), formal analysis (lead), writing – original draft (lead). **Larissa Bron:** conceptualization (equal), formal analysis (supporting), writing – review and editing (supporting). **Madison Carlson:** conceptualization (equal), writing – review and editing (supporting). **Sophia S. Labiy:** conceptualization (equal), writing – review and editing (supporting). **Zoe Penno:** conceptualization (equal), writing – review and editing (supporting). **Hayley Webster:** conceptualization (equal), writing – review and editing (supporting). **Jason T. Fisher:** supervision (lead), writing – review and editing (equal). **Marissa A. Dyck:** conceptualization (lead), data curation (lead), supervision (lead), writing – review and editing (equal).

## Conflicts of Interest

The authors declare no conflicts of interest.

## Supporting information


Appendix S1.

## Data Availability

All data are available at https://borealisdata.ca/.
