# Supplementary material for: Energy Infrastructure Clears the Way for Coyotes in Alberta's Oil Sands
Source: Ecol Evol. 2025 Aug 18;15(8):e71904. doi: 10.1002/ece3.71904 (PMC12360962; doi:10.1002/ece3.71904)
Supplement: Supplementary file 1 — Appendix S1. [file ECE3-15-e71904-s001.zip › coyote_analysis.pdf]

coyote_analysis

Jamie Clarke

last updated: 26/03/2025

about

This is a data analysis code walk-through for the manuscript *Energy infrastructure clears the way for coyotes in Alberta's oil sands*. It was written by Jamie F Clarke with help from Marissa A Dyck, and based on preliminary scripts by Larissa Bron. Before running this script, you will need to format the data using the coyote_formatting script. Happy analyzing :-)

set-up

start by loading in relevant packages:

```
library(tidyverse)
library(PerformanceAnalytics)
library(lme4)
library(MuMIn)
library(purrr)
library(broom.mixed)
library(car)
library(ggplot2)
library(cowplot)
library(ggpubr)
library(broom.mixed)
library(insight)
```

data import

read in processed data (created using coyote_formatting script):

```
coyote_data <-

  read_csv('data/processed/coyote_data.csv')
```

linear feature model set

step 1: doing some exploratory analyses to decide which linear features to include in further models

H0: null model

```
null <-

  glmer(
    cbind(coyote_pres, coyote_abs) ~ 1 +
      (1 | array),
    data = coyote_data,
    family = binomial)
```

H1: global model (all uncorrelated linear features)

```
global_lf <-

  glmer(
    cbind(coyote_pres, coyote_abs) ~
      scale(roads) +
      scale(seismic_lines) +
      scale(seismic_lines_3D) +
      scale(trails) +
      scale(transmission_lines) +
      (1 | array),
    data = coyote_data,
    family = binomial)
```

H2: pipelines (on their own since hard to classify, variable widths + correlated with other features)

```
pipeline_lf <-

  glmer(
    cbind(coyote_pres, coyote_abs) ~
      scale(pipeline) +
```

```
      (1 | array),
    data = coyote_data,
    family = binomial)
```

H3: narrow linear features (~ 5 m wide)

```
narrow_lf <-

  glmer(
  cbind(coyote_pres, coyote_abs) ~
    scale(seismic_lines_3D) +
    scale(trails) +
    (1 | array),
  data = coyote_data,
  family = binomial)
```

H4: wide linear features (> 5 m wide)

```
wide_lf <-

  glmer(
    cbind(coyote_pres, coyote_abs) ~
      scale(roads) +
      scale(seismic_lines) +
      scale(transmission_lines) +
      (1 | array),
    data = coyote_data,
    family = binomial)
```

H5: vegetated linear features (not paved/graveled)

```
veg_lf <-

  glmer(
    cbind(coyote_pres, coyote_abs) ~
      scale(seismic_lines) +
      scale(seismic_lines_3D) +
      scale(trails) +
      scale(transmission_lines) +
      (1 | array),
    data = coyote_data,
    family = binomial)
```

H6: un-vegetated linear features (paved/graveled)

```r
unveg_lf <-

  glmer(
    cbind(coyote_pres, coyote_abs) ~
      scale(roads) +
      (1 | array),
    data = coyote_data,
    family = binomial)
```

linear feature model selection

```r
lf_sel <-

  model.sel(null,
            global_lf,
            pipeline_lf,
            narrow_lf,
            wide_lf,
            veg_lf,
            unveg_lf)

# run lf_sel to see output
lf_sel
```

```
## Model selection table
##              (Int) scl(rds) scl(ssm_lns_3D) scl(ssm_lns) scl(trl) scl(trn_lns)
## wide_lf     -1.371   0.5861                      0.18440               0.01404
## global_lf   -1.371   0.5640        -0.06417      0.19330  0.05832      0.05274
## unveg_lf    -1.368   0.5513
## veg_lf      -1.363                 -0.13970     -0.00527  0.14850      0.20740
## narrow_lf   -1.363                 -0.04550               0.13650
## pipeline_lf -1.361
## null        -1.359
##             scl(ppl) df   logLik   AICc delta weight
## wide_lf               5 -485.086  980.4  0.00  0.582
## global_lf             7 -483.988  982.5  2.04  0.210
## unveg_lf              3 -488.193  982.5  2.05  0.208
## veg_lf                6 -527.796 1068.0 87.53  0.000
## narrow_lf             4 -533.175 1074.5 94.09  0.000
```

```
## pipeline_lf   0.1535  3 -534.338 1074.8 94.34  0.000
## null                   2 -537.258 1078.6 98.13  0.000
## Models ranked by AICc(x)
## Random terms (all models):
##   1 | array
```

result:
- wide_lf model best-supported by delta > 2.00
- global_lf model second-best supported

creating a wide linear feature variable

```
coyote_data <-

  coyote_data %>%

  mutate(wide_linear =
           roads +
           seismic_lines +
           transmission_lines)
```

hypothesis-testing model set

step 2: test hypotheses using wide_linear variable

H1: global model (natural landcover, wide linear features, all mammals)

```
global <-

  glmer(
    cbind(coyote_pres, coyote_abs) ~
      scale(nat_land) +
      scale(wide_linear) +
      scale(white_tailed_deer) +
      scale(moose) +
      scale(red_squirrel) +
      scale(snowshoe_hare) +
      scale(grey_wolf) +
      scale(lynx) +
      scale(fisher) +
```

```
      (1 | array),
    data = coyote_data,
    family = binomial)
```

H2: natural landcover

```
lc <-

  glmer(
    cbind(coyote_pres, coyote_abs) ~
      scale(nat_land) +
      (1 | array),
    data = coyote_data,
    family = binomial)
```

H3: wide linear features and natural landcover

```
wide_lc <-

  glmer(
    cbind(coyote_pres, coyote_abs) ~
      scale(wide_linear) +
      scale(nat_land) +
      (1 | array),
    data = coyote_data,
    family = binomial)
```

H4: prey species

```
prey <-

  glmer(
    cbind(coyote_pres, coyote_abs) ~
      scale(white_tailed_deer) +
      scale(moose) +
      scale(red_squirrel) +
      scale(snowshoe_hare) +
      (1 | array),
    data = coyote_data,
    family = binomial)
```

H5: prey species and natural landcover

```r
prey_lc <-

  glmer(
    cbind(coyote_pres, coyote_abs) ~
      scale(white_tailed_deer) +
      scale(moose) +
      scale(red_squirrel) +
      scale(snowshoe_hare) +
      scale(nat_land) +
      (1 | array),
    data = coyote_data,
    family = binomial)
```

H6: competitor species

```r
comp <-

  glmer(
    cbind(coyote_pres, coyote_abs) ~
      scale(grey_wolf) +
      scale(lynx) +
      scale(fisher) +
      (1 | array),
    data = coyote_data,
    family = binomial)
```

H7: competitor species and natural landcover

```r
comp_lc <-

  glmer(
    cbind(coyote_pres, coyote_abs) ~
      scale(grey_wolf) +
      scale(lynx) +
      scale(fisher) +
      scale(nat_land) +
      (1 | array),
    data = coyote_data,
    family = binomial)
```

H8: prey species and wide linear features

```
prey_wide <-

  glmer(
  cbind(coyote_pres, coyote_abs) ~
    scale(white_tailed_deer) +
    scale(moose) +
    scale(red_squirrel) +
    scale(snowshoe_hare) +
    scale(wide_linear) +
    (1 | array),
  data = coyote_data,
  family = binomial)
```

H9: competitor species and wide linear features

```
comp_wide <-

  glmer(
    cbind(coyote_pres, coyote_abs) ~
      scale(grey_wolf) +
      scale(lynx) +
      scale(fisher) +
      scale(wide_linear) +
      (1 | array),
    data = coyote_data,
    family = binomial)
```

H10: prey species, wide linear features and natural landcover

```
prey_wide_lc <-

  glmer(
    cbind(coyote_pres, coyote_abs) ~
      scale(white_tailed_deer) +
      scale(moose) +
      scale(red_squirrel) +
      scale(snowshoe_hare) +
      scale(wide_linear) +
      scale(nat_land) +
      (1 | array),
    data = coyote_data,
    family = binomial)
```

H11: competitor species, wide linear features and natural landcover

```r
comp_wide_lc <-

  glmer(
    cbind(coyote_pres, coyote_abs) ~
      scale(grey_wolf) +
      scale(lynx) +
      scale(fisher) +
      scale(nat_land) +
      scale(wide_linear) +
      (1 | array),
    data = coyote_data,
    family = binomial)
```

H12: global interaction model (natural landcover, all mammals, and interactions between wide linear features and top prey/competitor species)

```r
global_int <-

  glmer(
    cbind(coyote_pres, coyote_abs) ~
      scale(nat_land) +
      scale(white_tailed_deer) +
      scale(moose) +
      scale(red_squirrel) +
      scale(lynx) +
      scale(fisher) +
      scale(wide_linear) * scale(snowshoe_hare) +
      scale(wide_linear) * scale(grey_wolf) +
      (1 | array),
    data = coyote_data,
    family = binomial)
```

H13: prey interaction model (all prey species and interaction between wide linear features and top prey species)

```r
prey_int <-

  glmer(
    cbind(coyote_pres, coyote_abs) ~
      scale(white_tailed_deer) +
      scale(moose) +
      scale(red_squirrel) +
      scale(wide_linear) * scale(snowshoe_hare) +
      (1 | array),
```

```
    data = coyote_data,
    family = binomial)
```

H14: competitor interaction model (all competitor species and interaction between wide linear features and top competitor species)

```
comp_int <-

  glmer(
    cbind(coyote_pres, coyote_abs) ~
      scale(lynx) +
      scale(fisher) +
      scale(wide_linear) * scale(grey_wolf) +
      (1 | array),
    data = coyote_data,
    family = binomial)
```

H15: global prey interaction model (all prey species, natural landcover and interaction between wide linear features and top prey species)

```
prey_lc_int <-

  glmer(
    cbind(coyote_pres, coyote_abs) ~
      scale(white_tailed_deer) +
      scale(moose) +
      scale(red_squirrel) +
      scale(nat_land) +
      scale(wide_linear) * scale(snowshoe_hare) +
      (1 | array),
    data = coyote_data,
    family = binomial)
```

H16: global competitor interaction model (all competitor species, natural landcover and interaction between wide linear features and top competitor species)

```
comp_lc_int <-

  glmer(
    cbind(coyote_pres, coyote_abs) ~
      scale(lynx) +
      scale(fisher) +
      scale(nat_land) +
```

```
      scale(wide_linear) * scale(grey_wolf) +
      (1 | array),
    data = coyote_data,
    family = binomial)
```

comparing fixed vs random effects

testing an example model (global) with a random effect for array against the same model without a random effect (i.e., a fixed effect global model)

H17: fixed effect global model (natural landcover, wide linear features, all mammals) without random effect

```
fe_global <-

  glm(
    cbind(coyote_pres, coyote_abs) ~
      scale(nat_land) +
      scale(wide_linear) +
      scale(white_tailed_deer) +
      scale(moose) +
      scale(red_squirrel) +
      scale(snowshoe_hare) +
      scale(grey_wolf) +
      scale(lynx) +
      scale(fisher),
    data = coyote_data,
    family = binomial)
```

run model selection:

```
model.sel(global,
          fe_global)
```

```
## Model selection table
##             (Int) scl(fsh) scl(gry_wlf) scl(lyn) scl(mos) scl(nat_lnd)
## global     -1.435  0.01794       0.1895   0.1688 -0.06344      -0.4037
## fe_global -1.434  0.07783       0.1685   0.1525 -0.07579      -0.4229
##           scl(red_sqr) scl(snw_har) scl(wht_tld_der) scl(wid_lnr)    class
## global         0.08085       0.1883          0.06411       0.4967 glmerMod
## fe_global      0.11220       0.2116          0.13640       0.5204      glm
##           random df   logLik  AICc delta weight
```

```
## global          a 11 -449.342 921.9  0.00  0.999
## fe_global         10 -457.064 935.1 13.24  0.001
## Models ranked by AICc(x)
## Random terms:
##  a: 1 | array
```

result: random effects model best-supported, carry that forward

hypothesis-testing model selection

```
h_sel <-

  model.sel(null,
            global,
            lc,
            wide_lc,
            prey,
            prey_lc,
            comp,
            comp_lc,
            prey_wide,
            comp_wide,
            prey_wide_lc,
            comp_wide_lc,
            global_int,
            prey_int,
            comp_int,
            prey_lc_int,
            comp_lc_int)

# run h_sel to see output
h_sel
```

```
## Model selection table
##                (Int) scl(fsh) scl(gry_wlf) scl(lyn)   scl(mos) scl(nat_lnd)
## global        -1.435  0.01794      0.18950   0.1688 -0.0634400      -0.4037
## global_int    -1.451  0.00612      0.19680   0.1548 -0.0612300      -0.4155
## comp_wide_lc -1.422  0.01571      0.16610   0.2260                 -0.3984
## comp_lc_int   -1.438  0.01165      0.16740   0.2231                 -0.3985
## prey_lc_int   -1.398                                  0.0269600      -0.4074
## prey_wide_lc -1.401                                  0.0100200      -0.4001
```

```
## wide_lc      -1.383                                                      -0.3918
## prey_lc      -1.400                               0.0325900      -0.4220
## comp_lc      -1.408  0.03463      0.11870   0.2206                          -0.4050
## prey_wide    -1.388                              -0.0211600
## prey_int     -1.385                              -0.0113900
## comp_wide    -1.400  0.04164      0.14680   0.2301
## comp_int     -1.416  0.03830      0.14790   0.2275
## lc           -1.375                                                      -0.4007
## prey         -1.388                              -0.0009068
## comp         -1.388  0.06482      0.09415   0.2224
## null         -1.359
##              scl(red_sqr) scl(snw_har) scl(wht_tld_der) scl(wid_lnr)
## global            0.08085       0.1883          0.06411       0.4967
## global_int        0.07737       0.2212          0.04133       0.4890
## comp_wide_lc                                                  0.5365
## comp_lc_int                                                   0.5186
## prey_lc_int       0.08106       0.2634          0.04555       0.4618
## prey_wide_lc      0.09034       0.2217          0.05484       0.4461
## wide_lc                                                       0.4962
## prey_lc           0.11910       0.2382          0.01504
## comp_lc
## prey_wide         0.07239       0.2129          0.22430       0.4817
## prey_int          0.06597       0.2400          0.22090       0.4932
## comp_wide                                                     0.5549
## comp_int                                                      0.5353
## lc
## prey              0.10490       0.2328          0.20190
## comp
## null
##              scl(gry_wlf):scl(wid_lnr) scl(snw_har):scl(wid_lnr) df   logLik
## global                                                           11 -449.342
## global_int                    -0.09829                  -0.05045 13 -448.150
## comp_wide_lc                                                      7 -461.318
## comp_lc_int                   -0.08160                            8 -460.718
## prey_lc_int                                             -0.06731  9 -461.012
## prey_wide_lc                                                      8 -462.181
## wide_lc                                                           4 -479.026
## prey_lc                                                           7 -480.855
## comp_lc                                                           6 -489.063
## prey_wide                                                         7 -489.912
## prey_int                                                -0.04264  8 -489.425
## comp_wide                                                         6 -492.259
## comp_int                      -0.08085                            7 -491.648
## lc                                                                3 -503.736
## prey                                                              6 -512.199
```

```
## comp                                                               5 -522.625
## null                                                               2 -537.258
##                  AICc  delta weight
## global          921.9   0.00  0.739
## global_int      924.0   2.09  0.260
## comp_wide_lc    937.1  15.25  0.000
## comp_lc_int     938.1  16.20  0.000
## prey_lc_int     940.8  18.95  0.000
## prey_wide_lc    941.0  19.12  0.000
## wide_lc         966.2  44.34  0.000
## prey_lc         976.2  54.33  0.000
## comp_lc         990.5  68.62  0.000
## prey_wide       994.3  72.44  0.000
## prey_int        995.5  73.61  0.000
## comp_wide       996.9  75.01  0.000
## comp_int        997.8  75.91  0.000
## lc             1013.6  91.69  0.000
## prey           1036.8 114.89  0.000
## comp           1055.5 133.63  0.000
## null           1078.6 156.69  0.000
## Models ranked by AICc(x)
## Random terms (all models):
##   1 | array
```

result:
- global model best supported by delta > 2.00
- global_int model second-best supported

detection data summaries

sum the number of independent detections of each focal species using `purrr`:

```
coyote_data %>%

  select_if(is.numeric) %>% # only consider numeric data

  map_dbl(sum) # sum down each column
```

```
##          pipeline              roads      seismic_lines   seismic_lines_3D
##         3.1495689          1.0120405          1.6212058          1.3443708
##            trails transmission_lines           nat_land        coyote_pres
##         0.3438247          0.8075866        218.0484554                 NA
```

```
##         coyote_abs          coyote_tot              fisher       snowshoe_hare
##                 NA        1319.0000000         262.0000000        4571.0000000
##  white_tailed_deer                lynx        red_squirrel               moose
##       6143.0000000         526.0000000        2200.0000000         696.0000000
##          grey_wolf         wide_linear
##        226.0000000           3.4408329
```

count the number of camera stations where coyotes were detected:

```
sum(coyote_data$coyote_pres != 0,
    na.rm = TRUE)
```

```
## [1] 172
```

mean number of presences/absences:

```
coyote_data %>%

  select(coyote_pres, coyote_abs) %>%

  summary()
```

```
##   coyote_pres       coyote_abs
##  Min.   : 0.000   Min.   : 0.000
##  1st Qu.: 0.000   1st Qu.: 6.000
##  Median : 2.000   Median :10.000
##  Mean   : 2.707   Mean   : 8.927
##  3rd Qu.: 4.000   3rd Qu.:12.000
##  Max.   :14.000   Max.   :15.000
##  NA's   :1        NA's   :1
```

evaluation by simulation

this section is adapted from Ariel Muldoon's 'Simulate! Simulate!' series and work by Dr Andrew Barnas, with heaps of coding help from Andrew (thank you!)

create a data-generating, modelling and model-selecting function:

```
coyote_glmm_sim = function(n_cts = 40, # approximate number of cameras/array
                           n_arrays = 6, # number of arrays
                           n_obs = 1, # number of years cameras were deployed
                           n = n_cts * n_arrays * n_obs, # total observations
                           b0 = -1.4, # intercept value from global model
                           b1 = -0.40, # slope coefficient for nat_land
                           b2 = 0.50, # slope coefficient for wide_linear
                           b3 = 0.06, # slope coefficient for deer
                           b4 = -0.06, # slope coefficient for moose
                           b5 = 0.08, # slope coefficient for squirrels
                           b6 = 0.19, # slope coefficient for hares
                           b7 = 0.19, # slope coefficient for wolves
                           b8 = 0.17, # slope coefficient for lynx
                           b9 = 0.02, # slope coefficient for fishers
                           array_sd = 0.28)
{
  # assign camera and array IDs
  ct <- rep(1:(n_cts * n_arrays),
          each = n_obs)

  array <- rep (1:n_arrays,
               each = n_cts * n_obs) %>%
    as.factor()

  # simulate 'collected' data
  # for landscape data: proportional coverage ranges from 0 to 1
  sim_nat_land <- rep(runif(n_cts * n_arrays,
                            min = 0,
                            max = 1), # uniform draws from 0 to 1
                      each = n_obs)

  sim_wide_lf <- rep(runif(n_cts * n_arrays,
                           min = 0,
                           max = 1), # uniform draws from 0 to 1
                     each = n_obs)

  # for independent detection data: number of detections ranges from scaled min to max
  sim_wtd <- rep(runif(n_cts * n_arrays,
                       min = min(scale(coyote_data$white_tailed_deer)),
                       max = max(scale(coyote_data$white_tailed_deer))),
                 each = n_obs)

  sim_moose <- rep(runif(n_cts * n_arrays,
                         min = min(scale(coyote_data$moose)),
```

```
                    max = max(scale(coyote_data$moose))),
              each = n_obs)

sim_squirrel <- rep(runif(n_cts * n_arrays,
                          min = min(scale(coyote_data$red_squirrel)),
                          max = max(scale(coyote_data$red_squirrel))),
                    each = n_obs)

sim_hare <- rep(runif(n_cts * n_arrays,
                      min = min(scale(coyote_data$snowshoe_hare)),
                      max = max(scale(coyote_data$snowshoe_hare))),
                each = n_obs)

sim_wolf <- rep(runif(n_cts * n_arrays,
                      min = min(scale(coyote_data$grey_wolf)),
                      max = max(scale(coyote_data$grey_wolf))),
                each = n_obs)

sim_lynx <- rep(runif(n_cts * n_arrays,
                      min = min(scale(coyote_data$lynx)),
                      max = max(scale(coyote_data$lynx))),
                each = n_obs)

sim_fisher <- rep(runif(n_cts * n_arrays,
                        min = min(scale(coyote_data$fisher)),
                        max = max(scale(coyote_data$fisher))),
                  each = n_obs)

# simulate random effect of array
array_effect <- rep(rnorm(n_arrays,
                          mean = 0,
                          sd = array_sd),
                    each = n_cts * n_obs)

# calculate the linear predictor for each observation
linear_pred <-

  b0 +
  b1 * sim_nat_land +
  b2 * sim_wide_lf +
  b3 * sim_wtd +
  b4 * sim_moose +
  b5 * sim_squirrel +
  b6 * sim_hare +
```

```
  b7 * sim_wolf +
  b8 * sim_lynx +
  b9 * sim_fisher +
  array_effect

# convert linear predictors to probabilities, using logit link function
prob <- plogis(linear_pred)

# simulate Bernoulli trials based on probabilities, with variable effort per camera
# 4-15 sampling opportunities (= months) per trial - min/max deployment durations wh
total_trials <- sample(4:15,
                       n,
                       replace = TRUE)

# use random number generator to determine number of successes (presences) and failu
# run as many times as observations in dataset
present <- rbinom(n,
                  size = total_trials,
                  prob = prob)

absent <- total_trials - present

# wrap everything into dataframe
df <- data.frame(ct,
                 array,
                 array_effect,
                 sim_nat_land,
                 sim_wide_lf,
                 sim_wtd,
                 sim_moose,
                 sim_squirrel,
                 sim_hare,
                 sim_wolf,
                 sim_lynx,
                 sim_fisher,
                 linear_pred,
                 prob,
                 total_trials,
                 present,
                 absent)

# fit GLMMs to simulated data
sim_global <- glmer(
  cbind(present, absent) ~
```

```
    sim_nat_land +
    sim_wide_lf +
    sim_wtd +
    sim_moose +
    sim_squirrel +
    sim_hare +
    sim_wolf +
    sim_lynx +
    sim_fisher +
    (1|array),
  data = df,
  family = binomial)

sim_lc <- glmer(
  cbind(present, absent) ~
    sim_nat_land +
    (1|array),
  data = df,
  family = binomial)

sim_wide_lc <- glmer(
  cbind(present, absent) ~
    sim_nat_land +
    sim_wide_lf +
    (1|array),
  data = df,
  family = binomial)

sim_prey <- glmer(
  cbind(present, absent) ~
    sim_wtd +
    sim_moose +
    sim_squirrel +
    sim_hare +
    (1|array),
  data = df,
  family = binomial)

sim_prey_lc <- glmer(
  cbind(present, absent) ~
    sim_nat_land +
    sim_wtd +
    sim_moose +
    sim_squirrel +
```

```
    sim_hare +
    (1|array),
  data = df,
  family = binomial)

sim_comp <- glmer(
  cbind(present, absent) ~
    sim_wolf +
    sim_lynx +
    sim_fisher +
    (1|array),
  data = df,
  family = binomial)

sim_comp_lc <- glmer(
  cbind(present, absent) ~
    sim_nat_land +
    sim_wolf +
    sim_lynx +
    sim_fisher +
    (1|array),
  data = df,
  family = binomial)

sim_prey_wide <- glmer(
  cbind(present, absent) ~
    sim_wide_lf +
    sim_wtd +
    sim_moose +
    sim_squirrel +
    sim_hare +
    (1|array),
  data = df,
  family = binomial)

sim_comp_wide <- glmer(
  cbind(present, absent) ~
    sim_wide_lf +
    sim_wolf +
    sim_lynx +
    sim_fisher +
    (1|array),
  data = df,
  family = binomial)
```

```
sim_prey_wide_lc <- glmer(
  cbind(present, absent) ~
    sim_nat_land +
    sim_wide_lf +
    sim_wtd +
    sim_moose +
    sim_squirrel +
    sim_hare +
    (1|array),
  data = df,
  family = binomial)

sim_comp_wide_lc <- glmer(
  cbind(present, absent) ~
    sim_nat_land +
    sim_wide_lf +
    sim_wolf +
    sim_lynx +
    sim_fisher +
    (1|array),
  data = df,
  family = binomial)

sim_global_int <- glmer(
  cbind(present, absent) ~
    sim_nat_land +
    sim_wtd +
    sim_moose +
    sim_squirrel +
    sim_lynx +
    sim_fisher +
    sim_wide_lf * sim_hare +
    sim_wide_lf * sim_wolf +
    (1|array),
  data = df,
  family = binomial)

sim_prey_int <- glmer(
    cbind(present, absent) ~
      sim_wtd +
      sim_moose +
      sim_squirrel +
      sim_wide_lf * sim_hare +
      (1|array),
```

```
    data = df,
    family = binomial)

sim_comp_int <- glmer(
  cbind(present, absent) ~
    sim_lynx +
    sim_fisher +
    sim_wide_lf * sim_wolf +
    (1|array),
  data = df,
  family = binomial)

sim_prey_lc_int <- glmer(
  cbind(present, absent) ~
    sim_nat_land +
    sim_wtd +
    sim_moose +
    sim_squirrel +
    sim_wide_lf * sim_hare +
    (1|array),
  data = df,
  family = binomial)

sim_comp_lc_int <- glmer(
  cbind(present, absent) ~
    sim_nat_land +
    sim_lynx +
    sim_fisher +
    sim_wide_lf * sim_wolf +
    (1|array),
  data = df,
  family = binomial)

sim_hsel <- model.sel(null,
                      sim_global,
                      sim_lc,
                      sim_wide_lc,
                      sim_prey,
                      sim_prey_lc,
                      sim_comp,
                      sim_comp_lc,
                      sim_prey_wide,
                      sim_comp_wide,
                      sim_prey_wide_lc,
```

```
                        sim_comp_wide_lc,
                        sim_global_int,
                        sim_prey_int,
                        sim_comp_int,
                        sim_prey_lc_int,
                        sim_comp_lc_int)

  # return parameter name and beta coefficient for global model
  sim_pars <- get_parameters(sim_global)

  # transform sim_hsel into a tibble with models as rownames
  # extract top model
  top_mod <-  sim_hsel %>%

    as_tibble(rownames = 'model') %>%

    select(model) %>%

    slice(1)

  # return just the bits we want
  return(list(sim_pars,
              top_mod))
}
```

repeat the simulation for many iterations (in this case, 1,000):

```
glmm_sim <- replicate(1000,
                  coyote_glmm_sim(),
                  simplify = FALSE)
```

extract model coefficients:

```
sim_results <- glmm_sim %>%

  map_dfr(bind_rows,
          .id = 'simulation')

# run sim_results to see output
sim_results
```

save simulation results as .csv in 'processed' data folder:

```
write_csv(sim_results,
          'data/processed/simulation_output.csv')
```
