# Supplementary material for: Energy Infrastructure Clears the Way for Coyotes in Alberta's Oil Sands
Source: Ecol Evol. 2025 Aug 18;15(8):e71904. doi: 10.1002/ece3.71904 (PMC12360962; doi:10.1002/ece3.71904)
Supplement: Supplementary file 1 — Appendix S1. [file ECE3-15-e71904-s001.zip › coyote_figures.pdf]

coyote_figures

Jamie Clarke

last updated: 31/03/2025

about

This is a figure- and map-making code walkthrough for the manuscript *Energy infrastructure clears the way for coyotes in Alberta's oil sands*. It was written by Jamie F Clarke with help from Marissa A Dyck. Before running this script, you will need to format and analyze the data using the coyote_formatting and coyote_analysis scripts. The spatial data required to run the script is available on Jamie's GitHub, and/or in the links provided. Happy figure- and map-making :-)

set-up

start by loading in relevant packages:

```
library(tidyverse)
library(PerformanceAnalytics)
library(ggplot2)
library(cowplot)
library(sf)
library(dplyr)
library(tmap)
library(tmaptools)
library(ggpubr)
library(grid)
library(sp)
library(lme4)
library(ggeffects)
library(scales)
library(rphylopic)
library(MuMIn)
```

```r
library(car)
library(readr)
library(insight)
library(grt)
```

and setting the ggplot theme:

```r
theme_set(theme_classic())
```

data import

read in processed data (created using coyote_formatting script):

```r
coyote_data <-

  read_csv('data/processed/coyote_data.csv') %>%

  # add wide_linear column combining all wide linear features
  mutate(wide_linear =
           roads +
           seismic_lines +
           transmission_lines)
```

run top models (determined using coyote_analysis script):

```r
wide_lf <-

  glmer(
    cbind(coyote_pres, coyote_abs) ~
      scale(roads) +
      scale(seismic_lines) +
      scale(transmission_lines) +
      (1 | array),
    data = coyote_data,
    family = binomial)

global <-

  glmer(
    cbind(coyote_pres, coyote_abs) ~
      scale(nat_land) +
```

```
      scale(wide_linear) +
      scale(white_tailed_deer) +
      scale(moose) +
      scale(red_squirrel) +
      scale(snowshoe_hare) +
      scale(grey_wolf) +
      scale(lynx) +
      scale(fisher) +
      (1 | array),
    data = coyote_data,
    family = binomial)
```

covariate distribution plots

plot each covariate individually:

```
nat_land_dist <-

  ggplot(coyote_data,
         aes(x = nat_land)) +

  geom_density(fill = 'darkseagreen',
               color = 'darkseagreen') +

  geom_vline(xintercept = mean(coyote_data$nat_land),
             linetype = "dashed",
             linewidth = 0.7,
             alpha = 0.7) +

  xlab('proportion natural land') +

  ylab(' ') +

  scale_x_continuous(expand = c(0, 0),
                     limits = c(0.65, 1),
                     breaks = seq(0.65, 1, by = 0.1)) +

  scale_y_continuous(expand = c(0, 0)) +

  theme(axis.title.y = element_blank(),
        axis.text = element_text(size = 12),
        axis.title.x = element_text(size = 16),
```

```
      axis.title.y.left = element_text(size = 35))

wide_lf_dist <-

  ggplot(coyote_data,
         aes(x = wide_linear)) +

  geom_density(fill = 'darkseagreen',
               color = 'darkseagreen') +

  geom_vline(xintercept = mean(coyote_data$wide_linear),
             linetype = "dashed",
             linewidth = 0.7,
             alpha = 0.7) +

  xlab('proportion wide LFs') +

  ylab(' ') +

  scale_x_continuous(expand = c(0, 0),
                     breaks = breaks_pretty()) +

  scale_y_continuous(expand = c(0, 0)) +

  theme(axis.title.y = element_blank(),
        axis.text = element_text(size = 12),
        axis.title.x = element_text(size = 16),
        axis.title.y.left = element_text(size = 35))

fisher_dist <-

  ggplot(coyote_data,
         aes(x = fisher)) +

  geom_density(fill = 'lightsteelblue',
               color = 'lightsteelblue') +

  geom_vline(xintercept = mean(coyote_data$fisher),
             linetype = "dashed",
             linewidth = 0.7,
             alpha = 0.7) +

  xlab('total fisher detections') +
```

```r
  ylab(' ') +

  scale_x_continuous(expand = c(0, 0),
                     breaks = breaks_pretty()) +

  scale_y_continuous(expand = c(0, 0)) +

  theme(axis.title.y = element_blank(),
        axis.text = element_text(size = 12),
        axis.title.x = element_text(size = 16),
        axis.title.y.left = element_text(size = 35))

lynx_dist <-

  ggplot(coyote_data,
         aes(x = lynx)) +

  geom_density(fill = 'lightsteelblue',
               color = 'lightsteelblue') +

  geom_vline(xintercept = mean(coyote_data$lynx),
             linetype = "dashed",
             linewidth = 0.7,
             alpha = 0.7) +

  xlab('total lynx detections') +

  ylab(' ') +

  scale_x_continuous(expand = c(0, 0),
                     breaks = breaks_pretty()) +

  scale_y_continuous(expand = c(0, 0)) +

  theme(axis.title.y = element_blank(),
        axis.text = element_text(size = 12),
        axis.title.x = element_text(size = 16),
        axis.title.y.left = element_text(size = 35))

wolf_dist <-

  ggplot(coyote_data,
         aes(x = grey_wolf)) +
```

```
  geom_density(fill = 'lightsteelblue',
               color = 'lightsteelblue') +

  geom_vline(xintercept = mean(coyote_data$grey_wolf),
             linetype = "dashed",
             linewidth = 0.7,
             alpha = 0.7) +

  xlab('total wolf detections') +

  ylab(' ') +

  scale_x_continuous(expand = c(0, 0),
                     breaks = breaks_pretty()) +

  scale_y_continuous(expand = c(0, 0)) +

  theme(axis.title.y = element_blank(),
        axis.text = element_text(size = 12),
        axis.title.x = element_text(size = 16),
        axis.title.y.left = element_text(size = 35))

squirrel_dist <-

  ggplot(coyote_data,
         aes(x = red_squirrel)) +

  geom_density(fill = 'tomato',
               color = 'tomato') +

  geom_vline(xintercept = mean(coyote_data$red_squirrel),
             linetype = "dashed",
             linewidth = 0.7,
             alpha = 0.7) +

  xlab('total squirrel detections') +

  ylab(' ') +

  scale_x_continuous(expand = c(0, 0),
                     breaks = breaks_pretty()) +

  scale_y_continuous(expand = c(0, 0)) +
```

```r
  theme(axis.title.y = element_blank(),
        axis.text = element_text(size = 12),
        axis.title.x = element_text(size = 16),
        axis.title.y.left = element_text(size = 35))

hare_dist <-

  ggplot(coyote_data,
         aes(x = snowshoe_hare)) +

  geom_density(fill = 'tomato',
               color = 'tomato') +

  geom_vline(xintercept = mean(coyote_data$snowshoe_hare),
             linetype = "dashed",
             linewidth = 0.7,
             alpha = 0.7) +

  xlab('total hare detections') +

  ylab(' ') +

  scale_x_continuous(expand = c(0, 0),
                     breaks = breaks_pretty()) +

  scale_y_continuous(expand = c(0, 0)) +

  theme(axis.title.y = element_blank(),
        axis.text = element_text(size = 12),
        axis.title.x = element_text(size = 16),
        axis.title.y.left = element_text(size = 35))

deer_dist <-

  ggplot(coyote_data,
         aes(x = white_tailed_deer)) +

  geom_density(fill = 'tomato',
               color = 'tomato') +

  geom_vline(xintercept = mean(coyote_data$white_tailed_deer),
             linetype = "dashed",
             linewidth = 0.7,
             alpha = 0.7) +
```

```
  xlab('total deer detections') +

  ylab(' ') +

  scale_x_continuous(expand = c(0, 0),
                     breaks = breaks_pretty()) +

  scale_y_continuous(expand = c(0, 0)) +

  theme(axis.title.y = element_blank(),
        axis.text = element_text(size = 12),
        axis.title.x = element_text(size = 16),
        axis.title.y.left = element_text(size = 35))

moose_dist <-

  ggplot(coyote_data,
         aes(x = moose)) +

  geom_density(fill = 'tomato',
               color = 'tomato') +

  geom_vline(xintercept = mean(coyote_data$moose),
             linetype = "dashed",
             linewidth = 0.7,
             alpha = 0.7) +

  xlab('total moose detections') +

  ylab(' ') +

  scale_x_continuous(expand = c(0, 0),
                     breaks = breaks_pretty()) +

  scale_y_continuous(expand = c(0, 0)) +

  theme(axis.title.y = element_blank(),
        axis.text = element_text(size = 12),
        axis.title.x = element_text(size = 16),
        axis.title.y.left = element_text(size = 35))
```

covariate distribution plot panel

combine predicted probabilities plots together in one figure:

```
dist_plot <-

  ggarrange(nat_land_dist,
            wide_lf_dist,
            fisher_dist,
            lynx_dist,
            wolf_dist,
            squirrel_dist,
            hare_dist,
            deer_dist,
            moose_dist,
            font.label = list(size = 14),
            ncol = 3,
            nrow = 3) %>%

  annotate_figure(left = text_grob('density',
                                   rot = 90,
                                   vjust = 0.5,
                                   size = 22))

# run dist_plot to see figure
dist_plot
```

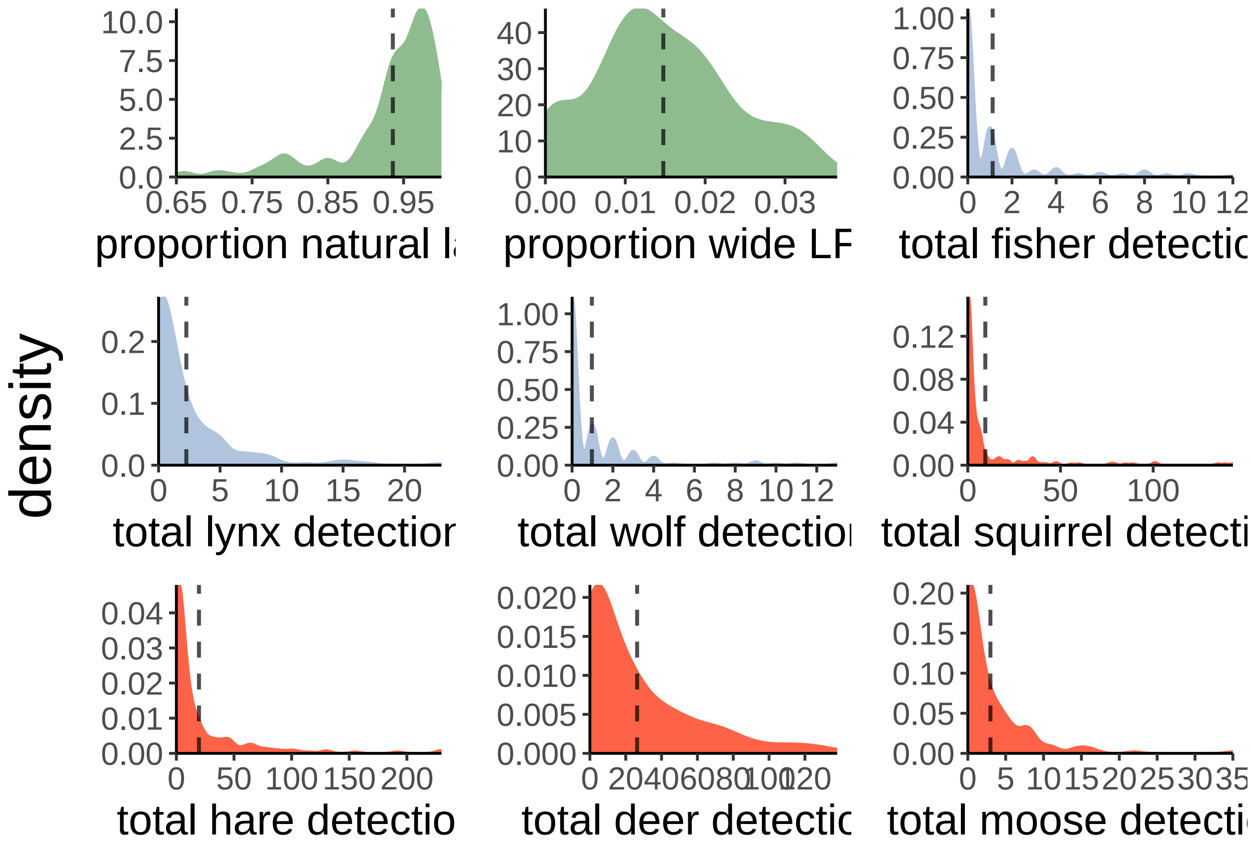

export covariate distribution figure

save `dist_plot` to 'figures' folder:

```
ggsave('covariate_distribution_panel.png',
       dist_plot,
       path = 'figures',
       width = 250,
       height = 250,
       units = 'mm',
       bg = 'white')
```

calculate odds ratios

step 1 - calculate odds ratio for wide linear features model:

```r
wide_lf_odds <-

  # calculate confidence intervals
  confint(wide_lf,
          parm = 'beta_') %>%

  # extract fixed effects coefficients
  cbind(est = fixef(wide_lf)) %>%

  # exponentiate to get odds ratios
  exp() %>%

  as.data.frame() %>%

  # name first column and preserve it for next step
  rownames_to_column(var = 'term') %>%

  # convert to tibble for easier manipulation
  as_tibble() %>%

  # remove intercept information
  filter(term != '(Intercept)') %>%

  # rename confidence %s
  rename(lower = '2.5 %',
         upper = '97.5 %') %>%

  # add a column with cleaned-up plot label names
  # (so we don't have to do this in ggplot2)
  add_column(label = c('roads',
                       'seismic lines',
                       'transmission lines')) %>%

  # change label column into a factor for plotting
  mutate(label = as.factor(label))
```

step 2 - calculate odds ratio for global model:

```r
global_odds <-

  confint(global,
          parm = 'beta_') %>%

  cbind(est = fixef(global)) %>%
```

```
  exp() %>%

  as.data.frame() %>%

  rownames_to_column(var = 'term') %>%

  as_tibble() %>%

  filter(term != '(Intercept)') %>%

  rename(lower = '2.5 %',
         upper = '97.5 %') %>%

  add_column(label = c('natural landcover',
                       'wide linear features',
                       'white-tailed deer',
                       'moose',
                       'red squirrel',
                       'snowshoe hare',
                       'grey wolf',
                       'lynx',
                       'fisher')) %>%

  mutate(label = as.factor(label))
```

odds ratios plots

step 1 - plotting wide linear features model:

```
odds_plot_1 <-

  ggplot(data = wide_lf_odds,
         aes(x = label,
             y = est)) +

  scale_y_continuous(limits = c(0.75, 2.15),
                     breaks = seq(0.8, 2.15, by = 0.2),
                     expand = c(0, 0)) +

  # add line at 1
  geom_hline(yintercept = 1,
             alpha = 0.7,
```

```
             linetype = 'dashed',
             linewidth = 0.7) +

  # add points for estimates
  geom_point(size = 3) +

  # add confidence intervals
  geom_linerange(aes(ymin = lower,
                     ymax = upper)) +

  # reverse the order of labels on the x axis
  # (ggplot annoyingly plots them in reverse order)
  scale_x_discrete(limits = rev) +

  # rename y axis title
  ylab('odds ratio') +

  # flip x and y axis
  coord_flip() +

  theme_classic() +

  # specify theme elements
  theme(panel.grid = element_blank(),
        axis.title.y = element_blank(),
        axis.text = element_text(size = 14),
        legend.position = 'none',
        axis.title.x = element_text(size = 16))

# run odds_plot_1 to see figure
odds_plot_1
```

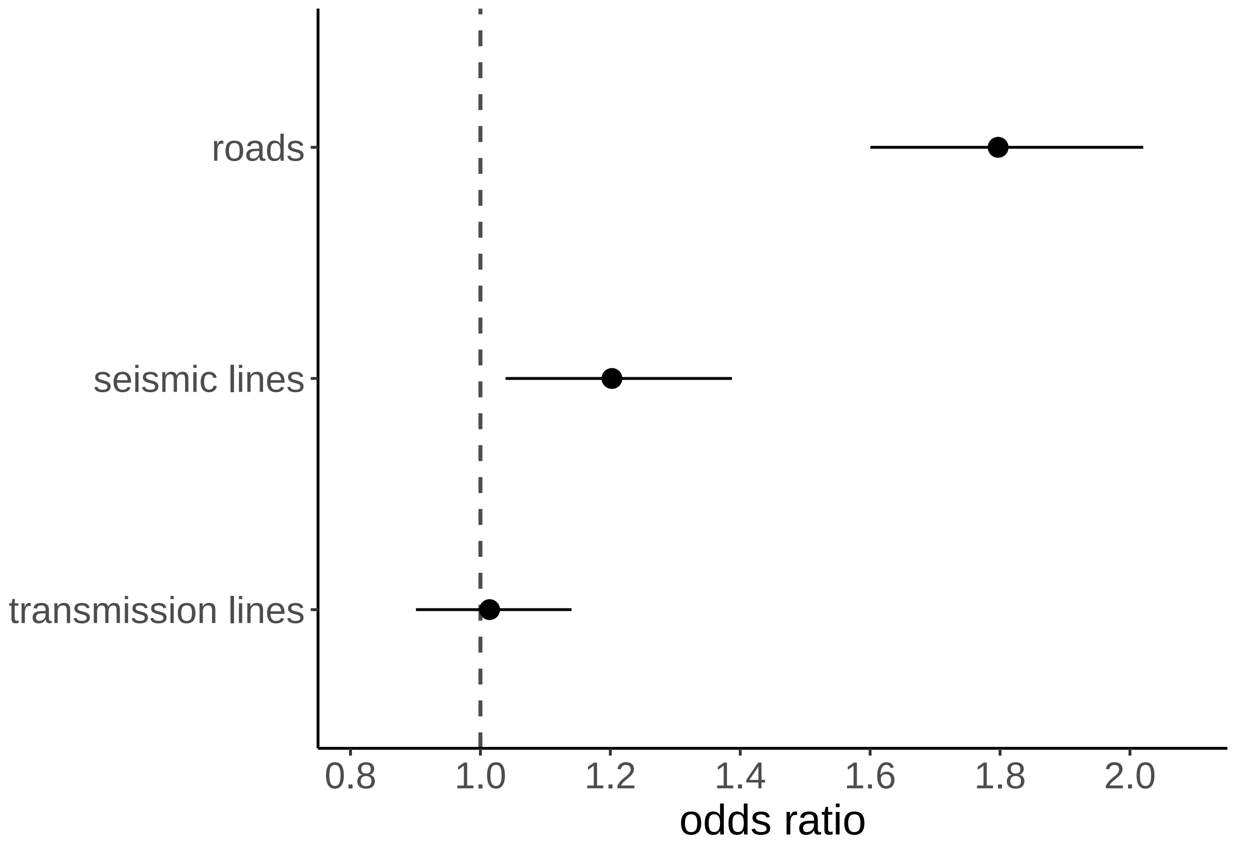

step 2 - plotting global model:

```
odds_plot_2 <-

  ggplot(data = global_odds %>%

           # customize order of labels
           mutate(label = fct_relevel(label,
                                      'natural landcover',
                                      'wide linear features',
                                      'fisher',
                                      'lynx',
                                      'grey wolf',
                                      'red squirrel',
                                      'snowshoe hare',
                                      'white-tailed deer',
                                      'moose')),
         aes(x = label,
             y = est)) +

  # add background for landscape covariates
  annotate('rect',
```

```
          xmin = 7.5,
          xmax = 10,
          ymin = -Inf,
          ymax = Inf,
          fill = 'darkseagreen',
          alpha = 0.4) +

# add background for competitor covariates
annotate('rect',
          xmin = 4.5,
          xmax = 7.5,
          ymin = -Inf,
          ymax = Inf,
          fill = 'lightsteelblue',
          alpha = 0.4) +

# add background for prey covariates
annotate('rect',
          xmin = 0,
          xmax = 4.5,
          ymin = -Inf,
          ymax = Inf,
          fill = 'tomato',
          alpha = 0.15) +

# add line at 1
geom_hline(yintercept = 1,
           alpha = 0.7,
           linetype = 'dashed',
           linewidth = 0.7) +

# add points for estimates
geom_point(size = 3) +

# add confidence intervals
geom_linerange(aes(ymin = lower,
                   ymax = upper),
               linewidth = 0.5) +

# reverse the order of labels on the x axis
# (ggplot annoyingly plots them in reverse order)
scale_x_discrete(limits = rev) +

# rename y axis title
```

```r
  ylab('odds ratio') +

  # flip x and y axis
  coord_flip() +

  theme_classic() +

  # specify theme elements
  theme(panel.grid = element_blank(),
        axis.title.y = element_blank(),
        axis.text = element_text(size = 14),
        legend.position = 'none',
        axis.title.x = element_text(size = 16))

# run odds_plot_2 to see figure
odds_plot_2
```

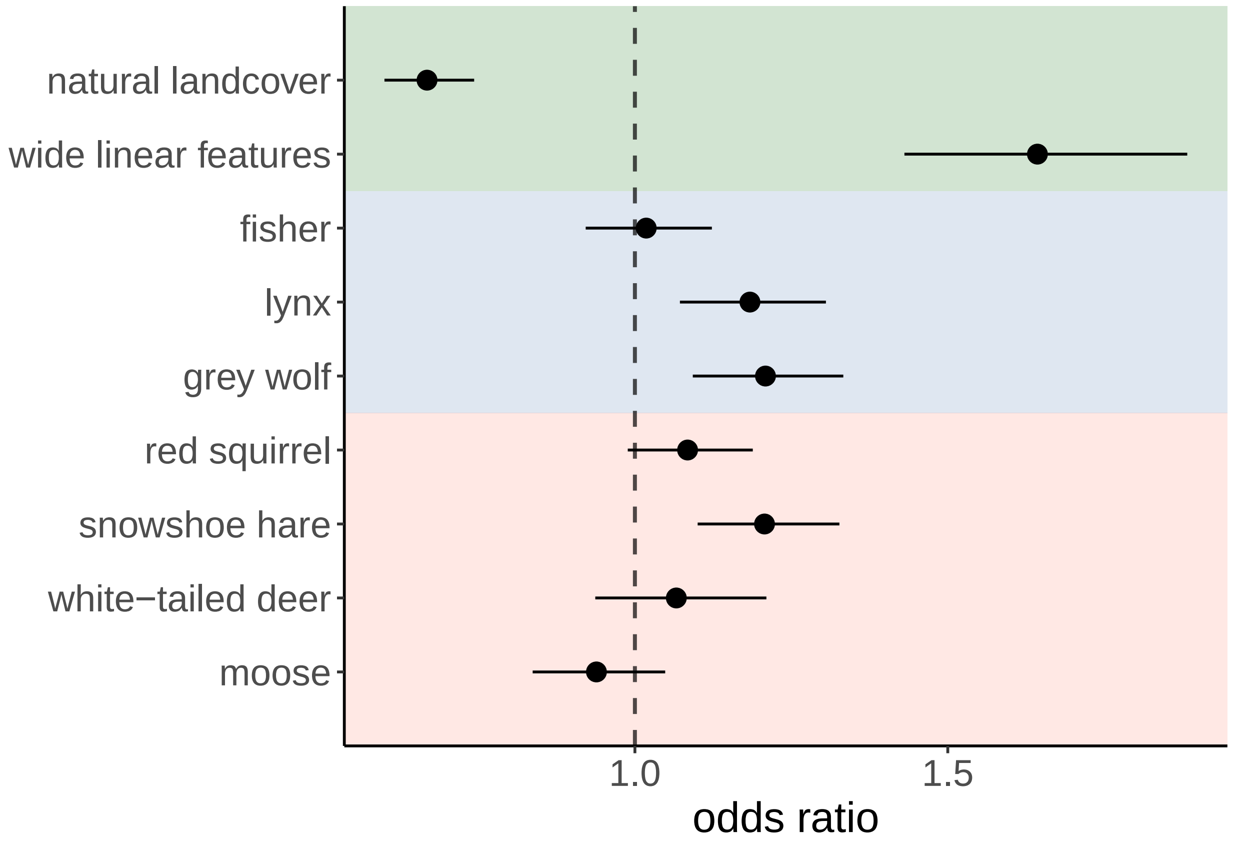

export odds ratio plots

save plots to 'figures' folder:

```
ggsave('odds_ratio_lf.tiff',
       odds_plot_1,
       width = 150,
       height = 100,
       units = 'mm',
       path = 'figures')

# save odds_plot_2 to 'figures' folder
ggsave('odds_ratio_h.tiff',
       odds_plot_2,
       width = 150,
       height = 100,
       units = 'mm',
       path = 'figures')
```

determine predicted probabilities

calculate predicted probabilities given each covariate of interest:

```
pp_nat_land <-

  ggpredict(global,
            terms = 'nat_land [all]',
            type = 'fe') # fe = fixed effects

pp_wide_linear <-

  ggpredict(global,
            terms = 'wide_linear [all]',
            type = 'fe')

pp_moose <-

  ggpredict(global,
            terms = 'moose [all]',
            type = 'fe')

pp_red_squirrel <-
```

```
  ggpredict(global,
            terms = 'red_squirrel [all]',
            type = 'fe')

pp_snowshoe_hare <-

  ggpredict(global,
            terms = 'snowshoe_hare [all]',
            type = 'fe')

pp_white_tailed_deer <-

  ggpredict(global,
            terms = 'white_tailed_deer [all]',
            type = 'fe')

pp_fisher <-

  ggpredict(global,
            terms = 'fisher [all]',
            type = 'fe')

pp_grey_wolf <-

  ggpredict(global,
            terms = 'grey_wolf [all]',
            type = 'fe')

pp_lynx <-

  ggpredict(global,
            terms = 'lynx [all]',
            type = 'fe')
```

predicted probabilities plots

side note - to find the PhyloPic uuid (= image ID), type the following code in the console:

```
pick_phylopic(name = 'Alces alces', n = 2)
```

where name is the scientific or common name of the species of interest and n is the number

of options you want to peruse; when you find the image you want, select it and find the uuid in the console output

plot predicted probability of coyote occurrence given proportion of natural landcover:

```
plot_nat_land <-

  ggplot(pp_nat_land,
         aes(x = x,
             y = predicted)) +

  geom_line(aes()) +

  geom_ribbon(aes(ymin = conf.low,
                  ymax = conf.high),
              fill = 'darkseagreen',
              alpha = 0.4) +

  scale_x_continuous(limits = c(0.7, 1.01),
                     breaks = seq(0.7, 1, by = 0.1),
                     expand = c(0, 0)) +

  scale_y_continuous(limits = c(0, 0.65),
                     breaks = seq(0, 0.65, by = 0.2),
                     expand = c(0, 0)) +

  xlab('natural landcover') +

  ylab(' ') +

  theme(axis.title.y = element_blank(),
        axis.text = element_text(size = 12),
        axis.title.x = element_text(size = 16),
        axis.title.y.left = element_text(size = 35))
```

plot predicted probability of coyote occurrence given proportion of wide linear features:

```
plot_wide_linear <-

  ggplot(pp_wide_linear,
         aes(x = x,
             y = predicted)) +

  geom_line(aes()) +
```

```
  geom_ribbon(aes(ymin = conf.low,
                  ymax = conf.high),
              fill = 'darkseagreen',
              alpha = 0.4) +

  scale_x_continuous(expand = c(0, 0),
                     labels = label_wrap(10)) +

  scale_y_continuous(limits = c(0, 0.65),
                     breaks = seq(0, 0.65, by = 0.2),
                     expand = c(0, 0)) +

  xlab('wide LFs') +

  ylab(' ') +

  theme(axis.title.y = element_blank(),
        axis.text = element_text(size = 12),
        axis.title.x = element_text(size = 16),
        axis.title.y.left = element_text(size = 35))
```

plot predicted probability of coyote occurrence given total independent moose detections:

```
plot_moose <-

  ggplot(pp_moose,
         aes(x = x,
             y = predicted)) +

  geom_line(aes()) +

  geom_ribbon(aes(ymin = conf.low,
                  ymax = conf.high),
              fill = 'tomato',
              alpha = 0.15) +

  scale_x_continuous(expand = c(0, 0),
                     labels = label_wrap(10)) +

  scale_y_continuous(limits = c(0, 0.65),
                     breaks = seq(0, 1, by = 0.2),
                     expand = c(0, 0)) +

  xlab('moose') +
```

```
  ylab(' ') +

  theme(axis.title.y = element_blank(),
        axis.text = element_text(size = 12),
        axis.title.x = element_text(size = 16),
        axis.title.y.left = element_text(size = 35)) +

  # add moose silhouette
  add_phylopic(uuid = '74eab34a-498c-4614-aece-f02361874f79',
               x = 10,
               y = 0.55,
               height = 0.2)
```

plot predicted probability of coyote occurrence given total independent red squirrel detections:

```
plot_red_squirrel <-

  ggplot(pp_red_squirrel,
         aes(x = x,
             y = predicted)) +

  geom_line(aes()) +

  geom_ribbon(aes(ymin = conf.low,
                  ymax = conf.high),
              fill = 'tomato',
              alpha = 0.15) +

  scale_x_continuous(limits = c(0, 153),
                     breaks = seq(0, 150, by = 50),
                     expand = c(0, 0),
                     labels = label_wrap(10)) +

  scale_y_continuous(limits = c(0, 0.65),
                     breaks = seq(0, 1, by = 0.2),
                     expand = c(0, 0)) +

  xlab('red squirrel') +

  ylab(' ') +

  theme(axis.title.y = element_blank(),
        axis.text = element_text(size = 12),
```

```
      axis.title.x = element_text(size = 16),
      axis.title.y.left = element_text(size = 35)) +

  # add squirrel silhouette
  add_phylopic(uuid = 'dad08fea-5263-4f57-a37b-c27cbe0eb9a5',
               x = 30,
               y = 0.54,
               height = 0.16)
```

plot predicted probability of coyote occurrence given total independent snowshoe hare detections:

```
plot_snowshoe_hare <-

  ggplot(pp_snowshoe_hare,
         aes(x = x,
             y = predicted)) +

  geom_line(aes()) +

  geom_ribbon(aes(ymin = conf.low,
                  ymax = conf.high),
              fill = 'tomato',
              alpha = 0.15) +

  scale_x_continuous(expand = c(0,0),
                     labels = label_wrap(10)) +

  scale_y_continuous(limits = c(0, 0.65),
                     breaks = seq(0, 1, by = 0.2),
                     expand = c(0, 0)) +

  xlab('snowshoe hare') +

  ylab(' ') +

  theme(axis.title.y = element_blank(),
        axis.text = element_text(size = 12),
        axis.title.x = element_text(size = 16),
        axis.title.y.left = element_text(size = 35)) +

  # add hare silhouette
  add_phylopic(uuid = '44f9db61-88e2-4a44-82f5-57dea9b3798d',
               x = 50,
```

```
              y = 0.55,
              height = 0.2)
```

plot predicted probability of coyote occurrence given total independent white-tailed deer detections:

```
plot_white_tailed_deer <-

  ggplot(pp_white_tailed_deer,
         aes(x = x,
             y = predicted)) +

  geom_line(aes()) +

  geom_ribbon(aes(ymin = conf.low,
                  ymax = conf.high),
              fill = 'tomato',
              alpha = 0.15) +

  scale_x_continuous(limits = c(0, 130),
                     breaks = seq(0, 130, by = 30),
                     expand = c(0, 0),
                     labels = label_wrap(10)) +

  scale_y_continuous(limits = c(0, 0.65),
                     breaks = seq(0, 1, by = 0.2),
                     expand = c(0, 0)) +

  xlab('white-tailed deer') +

  ylab(' ') +

  theme(axis.title.y = element_blank(),
        axis.text = element_text(size = 12),
        axis.title.x = element_text(size = 16),
        axis.title.y.left = element_text(size = 35)) +

  # add deer silhouette
  add_phylopic(uuid = '4584be20-4514-4673-a3e8-97e2a6a10e57',
              x = 29,
              y = 0.53,
              height = 0.24)
```

plot predicted probability of coyote occurrence given total independent fisher detections:

```r
plot_fisher <-

  ggplot(pp_fisher,
         aes(x = x,
             y = predicted)) +

  geom_line(aes()) +

  geom_ribbon(aes(ymin = conf.low,
                  ymax = conf.high),
              fill = 'lightsteelblue',
              alpha = 0.4) +

  scale_x_continuous(limits = c(0, 12.5),
                     breaks = seq(0, 12.5, by = 3),
                     expand = c(0, 0),
                     labels = wrap_format(10)) +

  scale_y_continuous(limits = c(0, 0.65),
                     breaks = seq(0, 1, by = 0.2),
                     expand = c(0, 0)) +

  xlab('fisher') +

  ylab(' ') +

  theme(axis.title.y = element_blank(),
        axis.text = element_text(size = 12),
        axis.title.x = element_text(size = 16),
        axis.title.y.left = element_text(size = 35)) +

  # add fisher silhouette
  add_phylopic(uuid = '735066c6-2f3e-4f97-acb1-06f55ae075c9',
               x = 3.5,
               y = 0.55,
               height = 0.12)
```

plot predicted probability of coyote occurrence given total independent grey wolf detections:

```r
plot_grey_wolf <-

  ggplot(pp_grey_wolf,
         aes(x = x,
```

```r
             y = predicted)) +

  geom_line(aes()) +

  geom_ribbon(aes(ymin = conf.low,
                   ymax = conf.high),
               fill = 'lightsteelblue',
               alpha = 0.4) +

  scale_x_continuous(limits = c(0, 13),
                     breaks = seq(0, 13, by = 3),
                     expand = c(0, 0),
                     labels = wrap_format(10)) +

  scale_y_continuous(limits = c(0, 0.65),
                     breaks = seq(0, 1, by = 0.2),
                     expand = c(0, 0)) +

  xlab('grey wolf') +

  ylab(' ') +

  theme(axis.title.y = element_blank(),
        axis.text = element_text(size = 12),
        axis.title.x = element_text(size = 16),
        axis.title.y.left = element_text(size = 35)) +

  # add wolf silhouette
  add_phylopic(uuid = '8cad2b22-30d3-4cbd-86a3-a6d2d004b201',
                x = 3.5,
                y = 0.55,
                height = 0.17)
```

plot predicted probability of coyote occurrence given total independent lynx detections:

```r
plot_lynx <-

  ggplot(pp_lynx,
         aes(x = x,
             y = predicted)) +

  geom_line(aes()) +

  geom_ribbon(aes(ymin = conf.low,
```

```
                ymax = conf.high),
            fill = 'lightsteelblue',
            alpha = 0.4) +

  scale_x_continuous(expand = c(0, 0),
                     labels = wrap_format(10)) +

  scale_y_continuous(limits = c(0, 0.65),
                     breaks = seq(0, 1, by = 0.2),
                     expand = c(0, 0)) +

  xlab('lynx') +

  ylab(' ') +

  theme(axis.title.y = element_blank(),
        axis.text = element_text(size = 12),
        axis.title.x = element_text(size = 16),
        axis.title.y.left = element_text(size = 35)) +

  # add lynx silhouette
  add_phylopic(uuid = '27a2173a-5903-46fc-83c5-29ed7f421046',
               x = 5.9,
               y = 0.55,
               height = 0.17)
```

predicted probabilities plot panel

combine predicted probabilities plots together in one figure:

```
pp_plot <-

  ggarrange(plot_nat_land,
            plot_wide_linear,
            plot_fisher,
            plot_lynx,
            plot_grey_wolf,
            plot_red_squirrel,
            plot_snowshoe_hare,
            plot_white_tailed_deer,
            plot_moose,
            ncol = 3,
```

```
            nrow = 3) %>%

  annotate_figure(left = text_grob('predicted coyote occurrence',
                                   rot = 90,
                                   vjust = 0.5,
                                   size = 20))

# run pp_plot to see figure
pp_plot
```

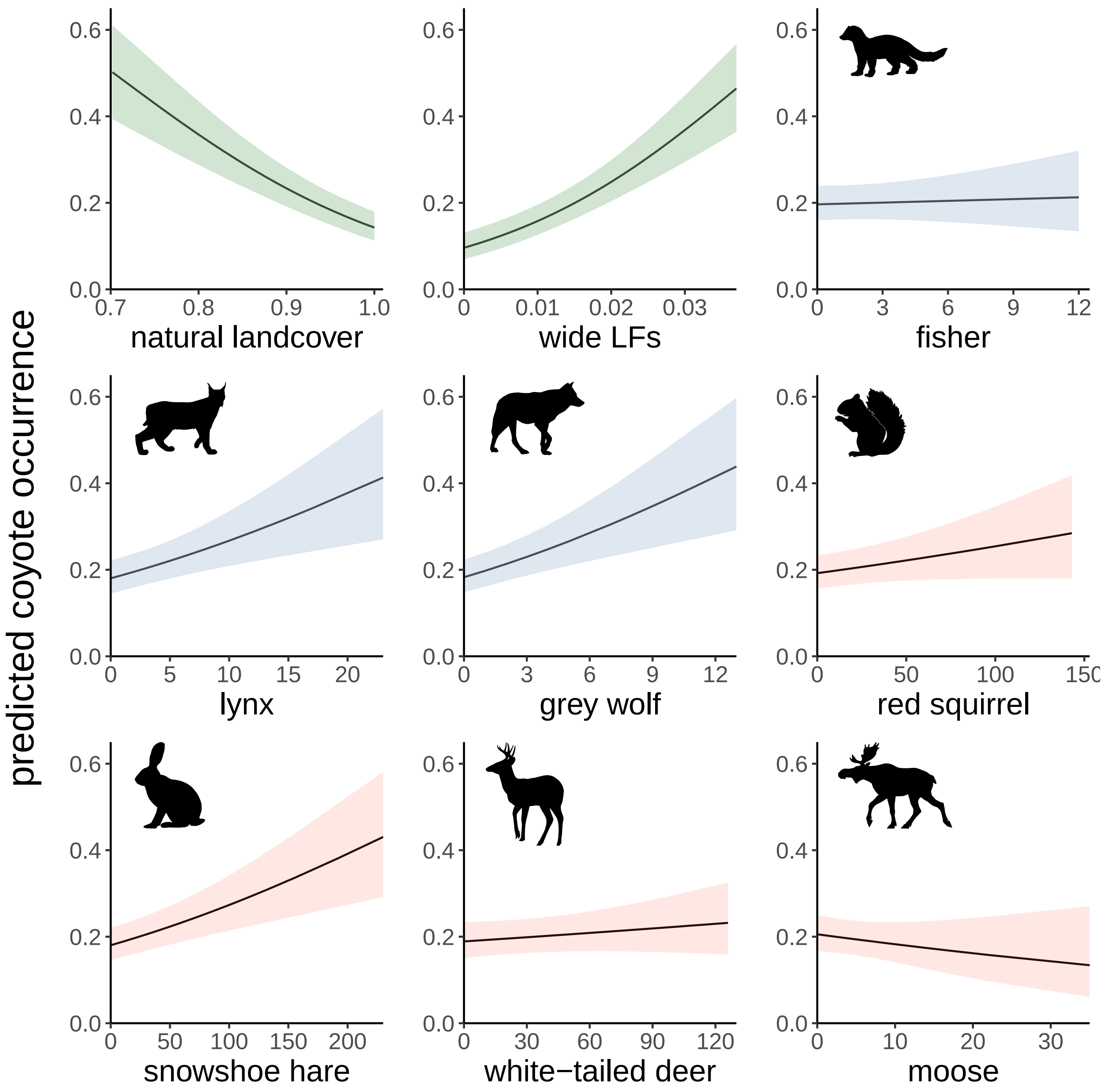

export predicted probabilities figure

save `pp_plot` to 'figures' folder:

```
ggsave('predicted_probabilities_panel.png',
       pp_plot,
       path = 'figures',
       width = 250,
       height = 250,
       units = 'mm',
       bg = 'white')
```

variance inflation factor

calculate VIFs:

```
# calculate VIFs for step 1
vif_wide_lf <-

  vif(wide_lf)

# calculate VIFs for step 2
vif_global <-

  vif(global) %>%

  # convert into a format that can be plotted
  as_tibble() %>%

  # add a column labelling covariate name
  add_column(label = c('natural landcover',
                       'wide linear features',
                       'fisher',
                       'lynx',
                       'grey wolf',
                       'red squirrel',
                       'snowshoe hare',
                       'white-tailed deer',
                       'moose')) %>%

  # add a column specifying covariate type
  add_column(cov_type = c('land',
```

```r
                          'land',
                          'comp',
                          'comp',
                          'comp',
                          'prey',
                          'prey',
                          'prey',
                          'prey'))
```

plot VIFs for step 2:

```r
vif_plot <-

  ggplot(data = vif_global %>%

           # customize order of labels
           mutate(label = fct_relevel(label,
                                      'natural landcover',
                                      'wide linear features',
                                      'fisher',
                                      'lynx',
                                      'grey wolf',
                                      'red squirrel',
                                      'snowshoe hare',
                                      'white-tailed deer',
                                      'moose')),
         aes(x = label,
             y = value,
             fill = cov_type)) +

  geom_col() +

  # set colour based on covariate type
  scale_fill_manual(values = c('lightsteelblue', 'darkseagreen', 'tomato'),
                    labels = c('land', 'comp', 'prey')) +

  # add line at 1
  geom_hline(yintercept = 1,
             alpha = 0.7,
             linetype = 'dashed',
             linewidth = 0.7) +

  # reverse the order of labels on the x axis (ggplot annoyingly plots them in reverse
  scale_x_discrete(limits = rev) +
```

```
  scale_y_continuous(limits = c(0, 1.35),
                     breaks = seq(0, 1.3, by = 0.5),
                     expand = c(0, 0)) +

  # rename x axis title
  ylab('VIF values') +

  # flip x and y axis
  coord_flip() +

  theme_classic() +

  # specify theme elements
  theme(axis.title.y = element_blank(),
        axis.text.y = element_text(size = 14),
        legend.position = 'none',
        axis.title.x = element_text(size = 16))

# run vif_plot to see figure
vif_plot
```

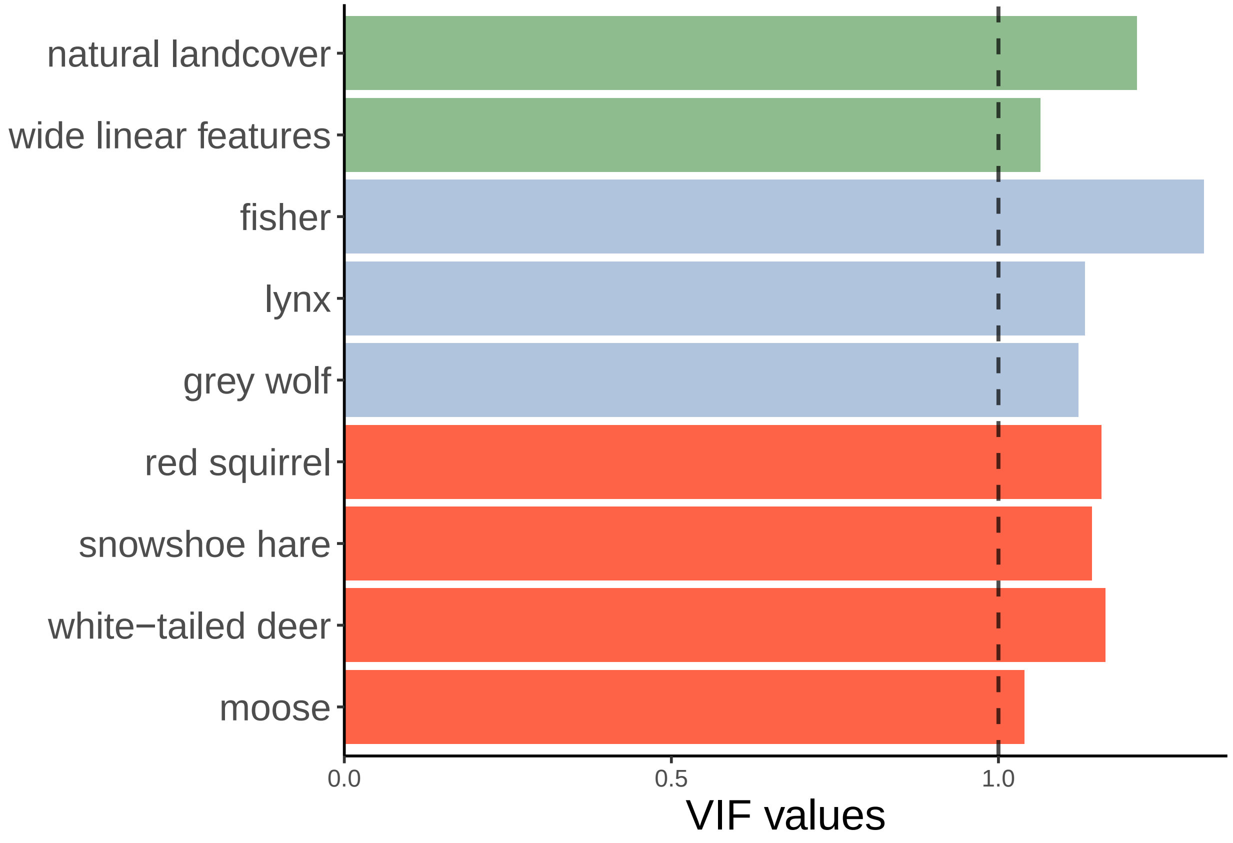

save `vif_plot` to 'figures' folder:

```
ggsave('vif_plot.png',
       vif_plot,
       path = 'figures',
       width = 100,
       height = 100,
       units = 'mm')
```

camera operability plot

this section is adapted from Marissa's OSM_2022-2023 script 1

read in deployment data:

```
deploy <-

  read_csv('data/raw/OSM_2021_2022_Deployment_Data.csv',

           # specify how to read in columns
           col_types = cols(Project.ID = readr::col_factor(),
                            Deployment.Location.ID = readr::col_factor(),
                            Camera.Deployment.Begin.Date = readr::col_date(
                              format = "%d-%b-%y"),
                            Camera.Deployment.End.Date = readr::col_date(
                              format = "%d-%b-%y"),
                            .default = readr::col_character())) %>%

  # set the column names to lower case
  set_names(
    names(.) %>%
      tolower() %>%

      # replace the '.' with '_'
      str_replace_all(pattern = '\\.',
                      replacement = '_')) %>%

  # rename start and end date so they are shorter, rename project_id and deployment_lo
  rename(start_date = camera_deployment_begin_date,
         end_date = camera_deployment_end_date,
         array = project_id,
         site = deployment_location_id) %>%
```

```
# rename site entries and remove prefix "OSM_" from array
# change LU01 to LU1 for consistency
# make array a factor
mutate(site = as.factor(case_when(site == 'LU15-44' ~ 'LU15_44',
                                  site == 'LI15_03' ~ 'LU15_03',
                                  TRUE  ~ site)),
       array = str_remove(array,
                          pattern = "OSM_"),
       array = dplyr::recode(array,
                             LU01 = 'LU1'),
       array = as.factor(array)) %>%

# remove columns we don't need
dplyr::select(!c(camera_failure_details,
          deployment_id)) %>%

# remove ABMI sites
na.omit() %>%

# to correct order of arrays in plot: make a new column with just LU number...
mutate(lu = str_remove(array,
                       pattern = 'LU'),
       lu = as.numeric(lu)) %>%

# arrange smallest to largest...
arrange(-lu) %>%

# and make another column with unique ascending values
# reorder according to values
mutate(value = 1:233,
       site_new = fct_reorder(site,
                              value))
```

calculate number of operational days:

```
ct_op <- deploy %>%

  # first group by site to calculate days camera at each site was operating for
  group_by(site) %>%

  # subtract end date from start date to get total number of days (adding 1 to ensure
  summarise(days_active = sum(end_date - start_date + 1)) %>%

  # sum all active days
```

```
  summarise(total_camera_days = sum(days_active))
```

plot camera operability:

```
ct_op_plot <-

  ggplot(deploy %>%

           # customize order of labels
           mutate(array = fct_relevel(array,
                                      'LU1',
                                      'LU2',
                                      'LU3',
                                      'LU13',
                                      'LU15',
                                      'LU21')),
         aes(color = array)) +

  geom_segment(aes(x = start_date,
                   xend = end_date,
                   y = site_new,
                   yend = site_new),
               linewidth = 0.7) +

  # set colours
  scale_color_manual(values = c('#332288', '#88CCEE', '#44AA99', '#117733', '#999933',

  xlab('date') +

  ylab('camera trap site') +

  theme(axis.text = element_text(size = 14),
        axis.text.y = element_blank(),
        axis.ticks.y = element_blank(),
        axis.title = element_text(size = 16),
        axis.title.y = element_text(vjust = +2),
        legend.text = element_text(size = 16),
        legend.title = element_blank())

# run ct_op_plot to see figure
ct_op_plot
```

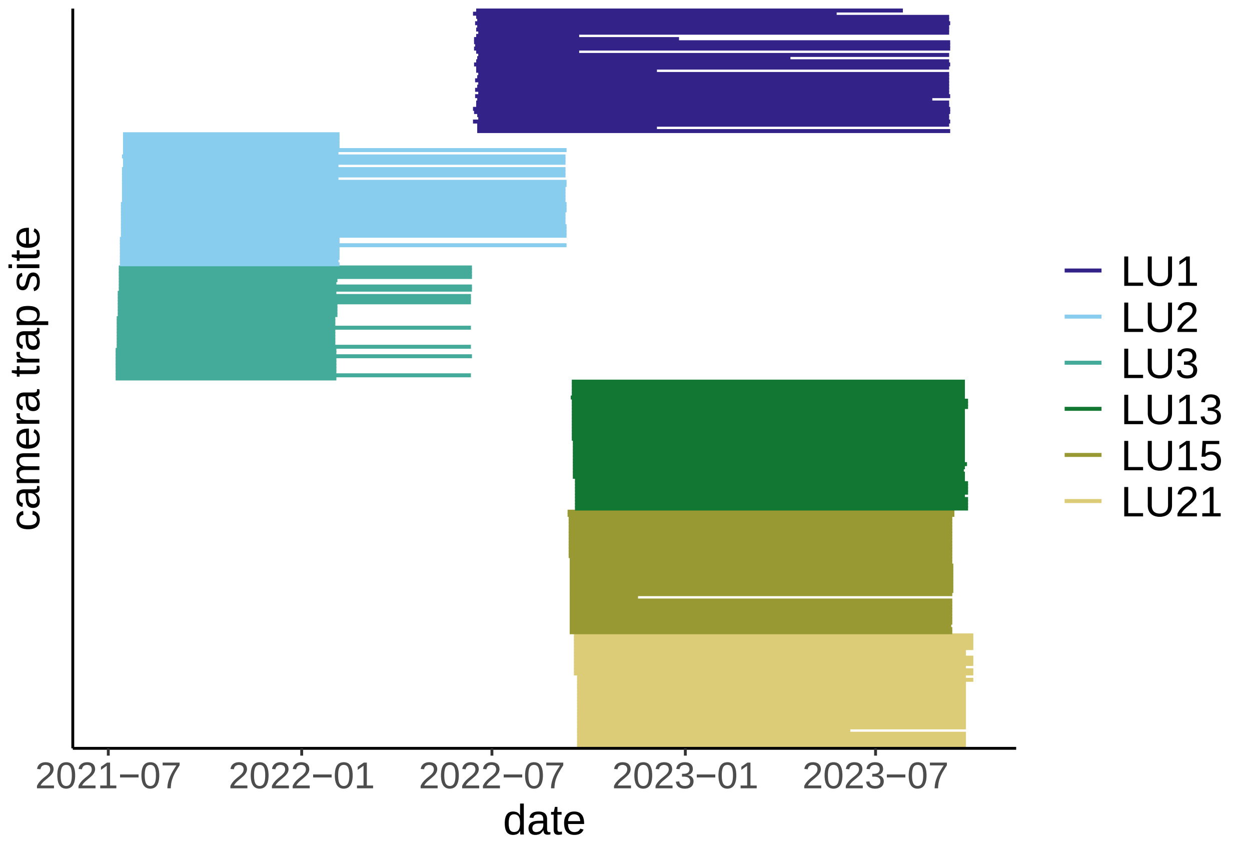

save `ct_op_plot` to 'figures' folder:

```
ggsave('ct_op.png',
       ct_op_plot,
       path = 'figures',
       width = 150,
       height = 150,
       units = 'mm')
```

naive occupancy plot

arrange dataframe for plotting:

```
naive_occ <-

  coyote_data %>%

  dplyr::select(array, coyote_pres, coyote_abs) %>%

  # to correct order of arrays in plot: make a new column with just LU number...
```

```
  mutate(array = dplyr::recode(array,
                               LU01 = 'LU1'),
         array = as.factor(array),
         lu = str_remove(array,
                         pattern = 'LU'),
         lu = as.numeric(lu)) %>%

  # arrange smallest to largest...
  arrange(lu) %>%

  # make another column with unique ascending values
  # reorder according to values
  mutate(value = 1:233,
         order = fct_reorder(array,
                             value)) %>%

  # make a column for total months camera was active and naive occupancy
  mutate(active = (coyote_pres + coyote_abs),
         occ = (coyote_pres / active))
```

plot naive occupancy:

```
naive_occ_plot <-

  ggplot(naive_occ %>%

           mutate(array = fct_relevel(array,
                                      'LU1',
                                      'LU2',
                                      'LU3',
                                      'LU13',
                                      'LU15',
                                      'LU21')),
         aes(x = reorder(array, value),
             y = occ,
             fill = array,
             color = array))+

  geom_violin() +

  scale_fill_manual(values = c('#332288', '#88CCEE', '#44AA99', '#117733', '#999933', '#

  scale_color_manual(values = c('#332288', '#88CCEE', '#44AA99', '#117733', '#999933',
```

```r
  xlab('array') +

  ylab('naive monthly occupancy') +

  theme(axis.text = element_text(size = 14),
        axis.title = element_text(size = 16),
        axis.title.y = element_text(vjust = +2),
        legend.position = 'none')

# run naive_occ_plot to see figure
naive_occ_plot
```

```
## Warning: Removed 1 rows containing non-finite values (`stat_ydensity()`).
```

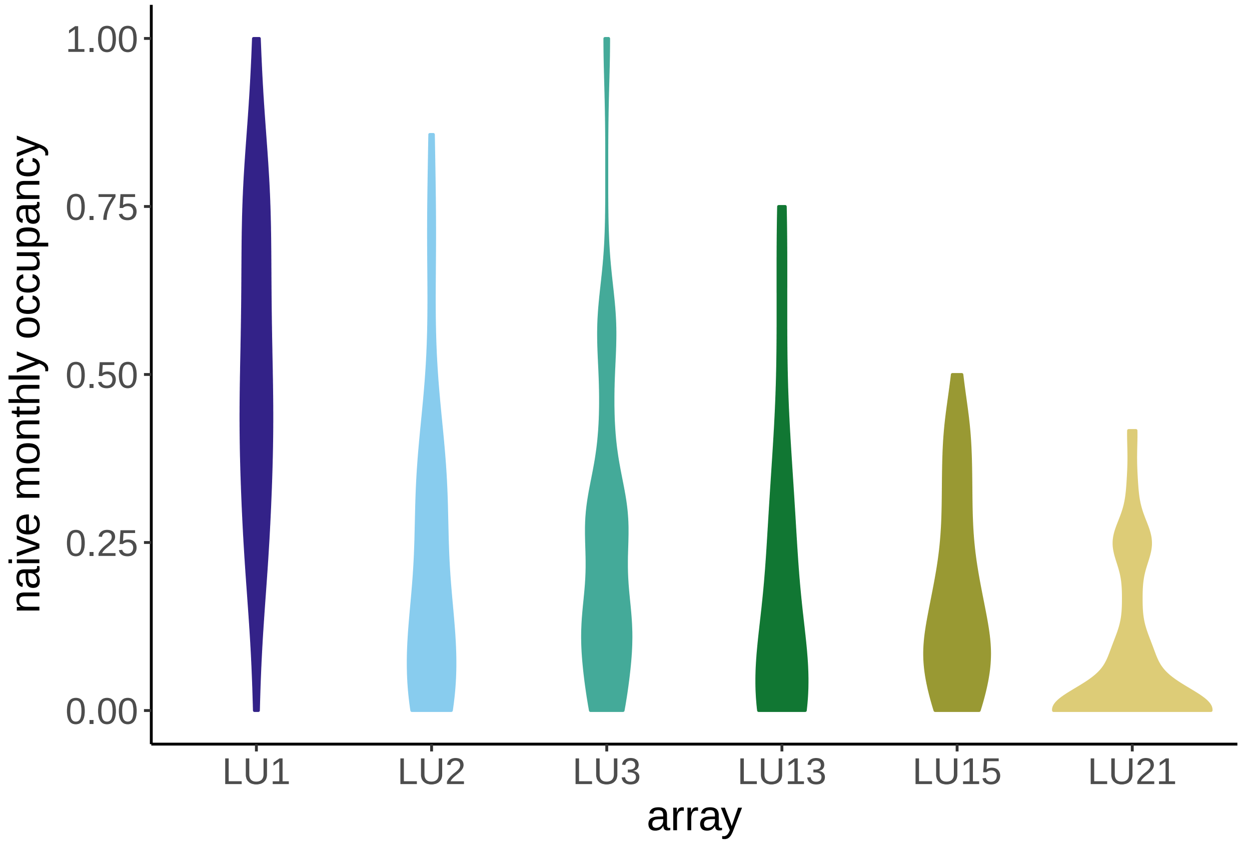

save `naive_occ_plot` to 'figures' folder:

```r
ggsave('naive_occupancy.png',
       naive_occ_plot,
       path = 'figures',
       width = 150,
```

```
        height = 150,
        units = 'mm')
```

simulation results

read in simulation results:

```
sim_results <-

  read_csv('data/processed/simulation_output.csv')
```

before plotting, extract 'true' parameter values:

```
nat_land_truth <-
  get_parameters(global) %>%
  filter(Parameter == 'scale(nat_land)') %>%
  pull(Estimate)

wide_linear_truth <-
  get_parameters(global) %>%
  filter(Parameter == 'scale(wide_linear)') %>%
  pull(Estimate)

fisher_truth <-
  get_parameters(global) %>%
  filter(Parameter == 'scale(fisher)') %>%
  pull(Estimate)

lynx_truth <-
  get_parameters(global) %>%
  filter(Parameter == 'scale(lynx)') %>%
  pull(Estimate)

grey_wolf_truth <-
  get_parameters(global) %>%
  filter(Parameter == 'scale(grey_wolf)') %>%
  pull(Estimate)

red_squirrel_truth <-
  get_parameters(global) %>%
  filter(Parameter == 'scale(red_squirrel)') %>%
  pull(Estimate)
```

```r
snowshoe_hare_truth <-
  get_parameters(global) %>%
  filter(Parameter == 'scale(snowshoe_hare)') %>%
  pull(Estimate)

white_tailed_deer_truth <-
  get_parameters(global) %>%
  filter(Parameter == 'scale(white_tailed_deer)') %>%
  pull(Estimate)

moose_truth <-
  get_parameters(global) %>%
  filter(Parameter == 'scale(moose)') %>%
  pull(Estimate)
```

plot the spread of simulated parameter estimates:

```r
d_nat_land <-

  ggplot(sim_results %>%
           filter(Parameter == 'sim_nat_land'),
         aes(x = Estimate)) +

  geom_density(fill = 'darkseagreen',
               color = 'darkseagreen') +

  geom_vline(xintercept = nat_land_truth,
             linetype = 'dashed',
             linewidth = 0.7) +

  scale_x_continuous(breaks = breaks_pretty(),
                     expand = c(0, 0)) +

  scale_y_continuous(expand = c(0, 0)) +

  xlab(' ') +

  ylab(' ') +

  ggtitle('natural land') +

  theme(axis.text = element_text(size = 14),
        plot.title = element_text(hjust = 0.5,
                                  size = 16,
```

```
                                     face = 'bold'))

d_wide_lf <-

  ggplot(sim_results %>%
           filter(Parameter == 'sim_wide_lf'),
         aes(x = Estimate)) +

  geom_density(fill = 'darkseagreen',
               color = 'darkseagreen') +

  geom_vline(xintercept = wide_linear_truth,
             linetype = 'dashed',
             linewidth = 0.7) +

  scale_x_continuous(breaks = breaks_pretty(),
                     expand = c(0, 0)) +

  scale_y_continuous(expand = c(0, 0)) +

  xlab(' ') +

  ylab(' ') +

  ggtitle('wide LFs') +

  theme(axis.text = element_text(size = 14),
        plot.title = element_text(hjust = 0.5,
                                  size = 16,
                                  face = 'bold'))

d_fisher <-

  ggplot(sim_results %>%
           filter(Parameter == 'sim_fisher'),
         aes(x = Estimate)) +

  geom_density(fill = 'lightsteelblue',
               color = 'lightsteelblue') +

  geom_vline(xintercept = fisher_truth,
             linetype = 'dashed',
             linewidth = 0.7) +
```

```r
  scale_x_continuous(breaks = breaks_pretty(),
                     expand = c(0, 0)) +

  scale_y_continuous(expand = c(0, 0)) +

  xlab(' ') +

  ylab(' ') +

  ggtitle('fisher') +

  theme(axis.text = element_text(size = 14),
        plot.title = element_text(hjust = 0.5,
                                  size = 16,
                                  face = 'bold'))

d_lynx <-

  ggplot(sim_results %>%
           filter(Parameter == 'sim_lynx'),
         aes(x = Estimate)) +

  geom_density(fill = 'lightsteelblue',
               color = 'lightsteelblue') +

  geom_vline(xintercept = lynx_truth,
             linetype = 'dashed',
             linewidth = 0.7) +

  scale_x_continuous(breaks = breaks_pretty(),
                     expand = c(0, 0)) +

  scale_y_continuous(expand = c(0, 0)) +

  xlab(' ') +

  ylab(' ') +

  ggtitle('lynx') +

  theme(axis.text = element_text(size = 14),
        plot.title = element_text(hjust = 0.5,
                                  size = 16,
                                  face = 'bold'))
```

```r
d_wolf <-

  ggplot(sim_results %>%
           filter(Parameter == 'sim_wolf'),
         aes(x = Estimate)) +

  geom_density(fill = 'lightsteelblue',
               color = 'lightsteelblue') +

  geom_vline(xintercept = grey_wolf_truth,
             linetype = 'dashed',
             linewidth = 0.7) +

  scale_x_continuous(breaks = breaks_pretty(),
                     expand = c(0, 0)) +

  scale_y_continuous(expand = c(0, 0)) +

  xlab(' ') +

  ylab(' ') +

  ggtitle('wolf') +

  theme(axis.text = element_text(size = 14),
        plot.title = element_text(hjust = 0.5,
                                  size = 16,
                                  face = 'bold'))

d_squirrel <-

  ggplot(sim_results %>%
           filter(Parameter == 'sim_squirrel'),
         aes(x = Estimate)) +

  geom_density(fill = 'tomato',
               color = 'tomato') +

  geom_vline(xintercept = red_squirrel_truth,
             linetype = 'dashed',
             linewidth = 0.7) +

  scale_x_continuous(breaks = breaks_pretty(),
                     expand = c(0, 0)) +
```

```
  scale_y_continuous(expand = c(0, 0)) +

  xlab(' ') +

  ylab(' ') +

  ggtitle('squirrel') +

  theme(axis.text = element_text(size = 14),
        plot.title = element_text(hjust = 0.5,
                                  size = 16,
                                  face = 'bold'))

d_hare <-

  ggplot(sim_results %>%
           filter(Parameter == 'sim_hare'),
         aes(x = Estimate)) +

  geom_density(fill = 'tomato',
               color = 'tomato') +

  geom_vline(xintercept = snowshoe_hare_truth,
             linetype = 'dashed',
             linewidth = 0.7) +

  scale_x_continuous(breaks = breaks_pretty(),
                     expand = c(0, 0)) +

  scale_y_continuous(expand = c(0, 0)) +

  xlab(' ') +

  ylab(' ') +

  ggtitle('hare') +

  theme(axis.text = element_text(size = 14),
        plot.title = element_text(hjust = 0.5,
                                  size = 16,
                                  face = 'bold'))

d_wtd <-
```

```r
  ggplot(sim_results %>%
           filter(Parameter == 'sim_wtd'),
         aes(x = Estimate)) +

  geom_density(fill = 'tomato',
               color = 'tomato') +

  geom_vline(xintercept = white_tailed_deer_truth,
             linetype = 'dashed',
             linewidth = 0.7) +

  scale_x_continuous(breaks = breaks_pretty(),
                     expand = c(0, 0)) +

  scale_y_continuous(expand = c(0, 0)) +

  xlab(' ') +

  ylab(' ') +

  ggtitle('deer') +

  theme(axis.text = element_text(size = 14),
        plot.title = element_text(hjust = 0.5,
                                  size = 16,
                                  face = 'bold'))

d_moose <-

  ggplot(sim_results %>%
           filter(Parameter == 'sim_moose'),
         aes(x = Estimate)) +

  geom_density(fill = 'tomato',
               color = 'tomato') +

  geom_vline(xintercept = moose_truth,
             linetype = 'dashed',
             linewidth = 0.7) +

  scale_x_continuous(limits = c(-0.129, 0.00251),
                     breaks = seq(-0.12, 0.00251, by = 0.05),
                     expand = c(0, 0)) +
```

```
  scale_y_continuous(expand = c(0, 0),
                     breaks = breaks_pretty()) +

  xlab(' ') +

  ylab('') +

  ggtitle('moose') +

  theme(axis.text = element_text(size = 14),
        plot.title = element_text(hjust = 0.5,
                                  size = 16,
                                  face = 'bold'))
```

arrange density plots into a single panel:

```
d_plot <-

  ggarrange(d_nat_land,
            d_wide_lf,
            d_fisher,
            d_lynx,
            d_wolf,
            d_squirrel,
            d_hare,
            d_wtd,
            d_moose,
            labels = NULL,
            label.x = 0.88,
            font.label = list(size = 14),
            ncol = 3,
            nrow = 3) %>%

  annotate_figure(left = text_grob('density',
                                   rot = 90,
                                   vjust = 0.5,
                                   size = 20)) %>%

  annotate_figure(bottom = text_grob('beta coefficient estimate',
                                     hjust = 0.5,
                                     size = 20))

# run d_plot to see figure
d_plot
```

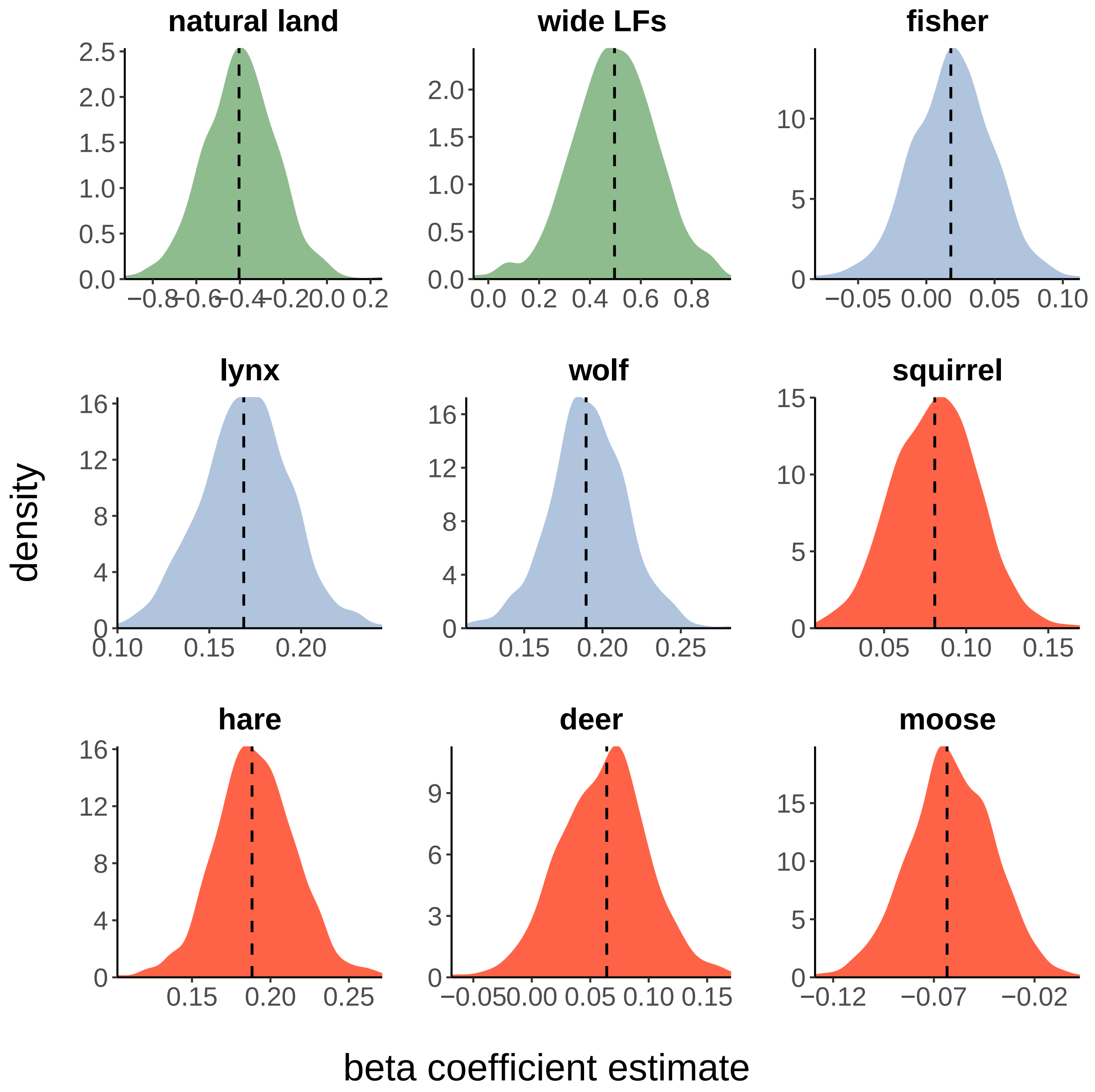

save `d_plot` to 'figures' folder:

```
ggsave('simulated_parameters_panel.png',
       d_plot,
       path = 'figures',
       width = 250,
       height = 250,
       units = 'mm',
       bg = 'white')
```

plot a bar graph of simulated model selection outcomes:

```r
sel_plot <-

  ggplot(sim_results %>%
           dplyr::select(model) %>%
           na.omit(),
         aes(x = model)) +

  geom_bar(fill = 'darkgrey') +

  scale_x_discrete(labels = c ("sim_global" = "global",
                               "sim_global_int" = "global interaction")) +

  scale_y_continuous(limits = c(0, 1000),
                     breaks = seq(0, 1000, by = 200),
                     expand = c(0, 0)) +

  theme(axis.text = element_text(size = 14),
        axis.title.x = element_text(size = 16),
        axis.title.y = element_text(size = 16))

# run sel_plot to see figure
sel_plot
```

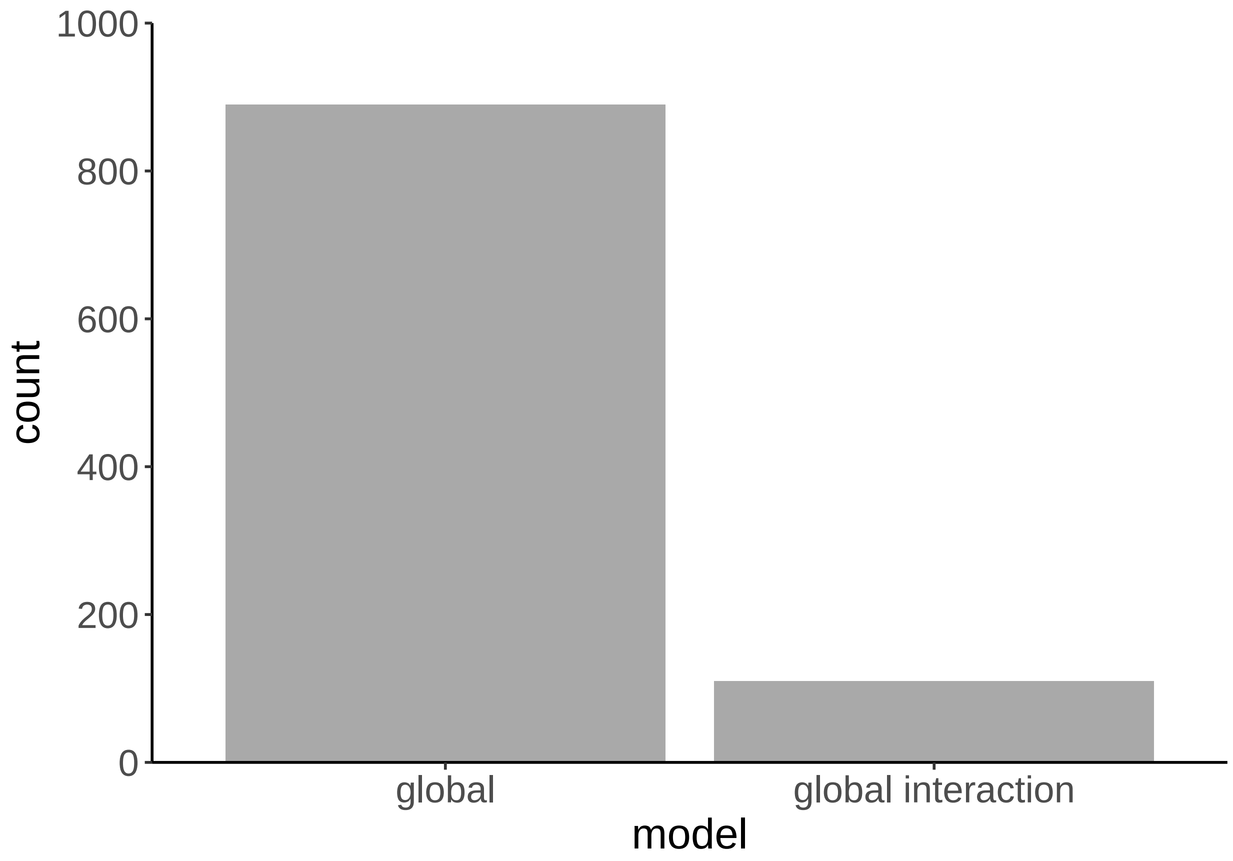

save `sel_plot` to 'figures' folder:

```
ggsave('simulated_top_models.png',
       sel_plot,
       path = 'figures',
       width = 150,
       height = 100,
       units = 'mm',
       bg = 'white')
```

mapping set-up

read in LU polygons:

```
# first: specify which LUs
# for 2021-2022 - LUs 2 + 3
# for 2022-2023 - LUs 1, 12, 15 + 21
target <- c(2, 3, 1, 13, 15, 21)
```

```
# then: read in data + filter for those LUs
lus <-

  st_read('data/spatial/ALL_LUs.shp') %>%

  set_names(
    names(.) %>%
      tolower()) %>%

  st_transform(crs = 26912) %>%

  filter(lu %in% target)
```

```
## Reading layer `ALL_LUs' from data source
##   `/Users/jamiefclarke/Documents/MASTERS/COURSEWORK/ES582/COYOTE PAPER/osm_coyote_glm
##   using driver `ESRI Shapefile'
## Simple feature collection with 12 features and 11 fields
## Geometry type: POLYGON
## Dimension:     XY
## Bounding box:  xmin: 496009.6 ymin: 6044552 xmax: 818907.2 ymax: 6406713
## Projected CRS: NAD83 / Alberta 10-TM (Forest)
```

read in CT deployment data for 2022:

```
cts_2022 <-

  st_read('data/spatial/OSM2022_SITES_ALL.shp') %>%

  set_names(
    names(.) %>%
      tolower())
```

```
## Reading layer `OSM2022_SITES_ALL' from data source
##   `/Users/jamiefclarke/Documents/MASTERS/COURSEWORK/ES582/COYOTE PAPER/osm_coyote_glm
##   using driver `ESRI Shapefile'
## Simple feature collection with 155 features and 19 fields
## Geometry type: POINT
## Dimension:     XY
## Bounding box:  xmin: 371208.6 ymin: 6046183 xmax: 559032.7 ymax: 6340373
## Projected CRS: NAD83 / UTM zone 12N
```

read in CT deployment data for 2021 per LU and combine into single dataframe:

```r
cts_2021 <-

  st_read('data/spatial/OSM_2021_LU2.shp') %>%
  rbind(st_read('data/spatial/OSM_2021_LU3.shp')) %>%

  set_names(
    names(.) %>%
      tolower())
```

```
## Reading layer `OSM_2021_LU2' from data source
##   `/Users/jamiefclarke/Documents/MASTERS/COURSEWORK/ES582/COYOTE PAPER/osm_coyote_glm
##   using driver `ESRI Shapefile'
## Simple feature collection with 47 features and 13 fields
## Geometry type: POINT
## Dimension:     XY
## Bounding box:  xmin: 449361.5 ymin: 6078899 xmax: 477079.5 ymax: 6099490
## Projected CRS: WGS 84 / UTM zone 12N
## Reading layer `OSM_2021_LU3' from data source
##   `/Users/jamiefclarke/Documents/MASTERS/COURSEWORK/ES582/COYOTE PAPER/osm_coyote_glm
##   using driver `ESRI Shapefile'
## Simple feature collection with 46 features and 13 fields
## Geometry type: POINT
## Dimension:     XY
## Bounding box:  xmin: 480770 ymin: 6131585 xmax: 515778.2 ymax: 6159308
## Projected CRS: WGS 84 / UTM zone 12N
```

read in provincial boundaries:

```r
# source = Statistics Canada
provs <-

  st_read('data/spatial/lpr_000b16a_e.shp') %>%

  set_names(
    names(.) %>%
      tolower()) %>%

  st_transform(crs = 26912)
```

```
## Reading layer `lpr_000b16a_e' from data source
##   `/Users/jamiefclarke/Documents/MASTERS/COURSEWORK/ES582/COYOTE PAPER/osm_coyote_glm
##   using driver `ESRI Shapefile'
## Simple feature collection with 13 features and 6 fields
```

```
## Geometry type: MULTIPOLYGON
## Dimension:     XY
## Bounding box:  xmin: 3689439 ymin: 659338.9 xmax: 9015737 ymax: 5242179
## Projected CRS: PCS_Lambert_Conformal_Conic
```

subset Alberta:

```
ab_boundary <-

  provs %>%
  filter(prename == 'Alberta')
```

crop Alberta spatial info to northeast (oil sands region):

```
# figure out spatial extent of LUs
raster::extent(lus)
```

```
## class       : Extent
## xmin        : 368007.3
## xmax        : 561572
## ymin        : 6038172
## ymax        : 6354387
```

```
# make bounding box of NE Alberta based on extents
ne_ab_bbox <-

  st_bbox(c(xmin = 365982.3,
            xmax = 563572,
            ymin = 6036147,
            ymax = 6356412),
          crs = st_crs(26912))
```

read in Alberta roads layer:

```
# source = National Road Network
roads <-

  st_read('data/spatial/NRN_AB_14_0_ROADSEG.shp') %>%

  set_names(
    names(.) %>%
      tolower()) %>%
```

```r
  st_transform(crs = 26912) %>%

  # simplify layer to speed up mapping
  st_union()
```

```
## Reading layer `NRN_AB_14_0_ROADSEG' from data source
##   `/Users/jamiefclarke/Documents/MASTERS/COURSEWORK/ES582/COYOTE PAPER/osm_coyote_glm
##   using driver `ESRI Shapefile'
## Simple feature collection with 413349 features and 48 fields
## Geometry type: LINESTRING
## Dimension:     XY
## Bounding box:  xmin: -120.0014 ymin: 48.99741 xmax: -110.0046 ymax: 59.99955
## Geodetic CRS:  GCS_North_American_1983_CSRS98
```

read in cities layer:

```r
cities <-

  st_read('data/spatial/city_points.shp') %>%

  set_names(
    names(.) %>%
      tolower()) %>%

  st_transform(crs = 26912) %>%

  filter(name == 'Fort McMurray')
```

```
## Reading layer `city_points' from data source
##   `/Users/jamiefclarke/Documents/MASTERS/COURSEWORK/ES582/COYOTE PAPER/osm_coyote_glm
##   using driver `ESRI Shapefile'
## Simple feature collection with 664 features and 9 fields
## Geometry type: POINT
## Dimension:     XY
## Bounding box:  xmin: 185955.5 ymin: 5430152 xmax: 855098.4 ymax: 6638791
## Projected CRS: NAD_1983_Transverse_Mercator
```

map of study area

extend bounding box so map features (e.g., scale bar) don’t overlap with map itself:

```r
bbox1 <-  st_bbox(ne_ab_bbox)

xrange <- bbox1$xmax - bbox1$xmin

bbox1[1] <- bbox1[1] - (0.4 * xrange) # xmin - left

bbox1 <- bbox1 %>%
  st_as_sfc() # make bounding box an sf polygon
```

set tmap mode to `plot`:

```r
tmap_mode('plot')
```

map LUs and CT deployments in NE Alberta:

```r
lus_cts <-

  # this establishes the spatial extent of the map
  tm_shape(ab_boundary,
           bbox = bbox1) +
  tm_fill() +

  # map neighbouring provinces in the background
  tm_shape(provs) +
  tm_fill(col = 'grey20') +

  # layer Alberta on top
  tm_shape(ab_boundary,
           bbox = ne_ab_bbox) +
  tm_fill(col = 'grey70') +

  # add a polygon for LU1
  tm_shape(lus %>%
             filter(namerefere == 'LU1')) +
  tm_fill(col = 'grey95') +

  # add a polygon for LU2
  tm_shape(lus %>%
             filter(namerefere == 'LU2')) +
  tm_fill(col = 'grey95') +

  # add a polygon for LU3
  tm_shape(lus %>%
```

```r
            filter(namerefere == 'LU3')) +
tm_fill(col = 'grey95') +

# add a polygon for LU13
tm_shape(lus %>%
            filter(namerefere == 'LU13')) +
tm_fill(col = 'grey95') +

# add a polygon for LU15
tm_shape(lus %>%
            filter(namerefere == 'LU15')) +
tm_fill(col = 'grey95') +

# add a polygon for LU21
tm_shape(lus %>%
            filter(namerefere == 'LU21')) +
tm_fill(col = 'grey95') +

# layer roads and cities on top
tm_shape(roads) +
tm_lines(col = 'grey40',
         lwd = 1) +

tm_shape(cities) +
tm_dots(col = 'black',
        size = 1) +
tm_text('name',
        size = 1.8,
        just = c(-0.08,-0.08),
        fontface = 'bold') +

# add points for CT deployment locations in LU1
tm_shape(cts_2022 %>%
            crop_shape(lus %>%
                         filter(namerefere == 'LU1'))) +
tm_dots(col = '#005AB5',
        size = 0.2) +

# add points for CT deployment locations in LU2
tm_shape(cts_2021 %>%
            crop_shape(lus %>%
                         filter(namerefere == 'LU2'))) +
tm_dots(col = '#DC3220',
        size = 0.2) +
```

```r
# add points for CT deployment locations in LU3
tm_shape(cts_2021 %>%
           crop_shape(lus %>%
                        filter(namerefere == 'LU3'))) +
tm_dots(col = '#DC3220',
        size = 0.2) +

# add points for CT deployment locations in LU13
tm_shape(cts_2022 %>%
           crop_shape(lus %>%
                        filter(namerefere == 'LU13'))) +
tm_dots(col = '#005AB5',
        size = 0.2) +

# add points for CT deployment locations in LU15
tm_shape(cts_2022 %>%
           crop_shape(lus %>%
                        filter(namerefere == 'LU15'))) +
tm_dots(col = '#005AB5',
        size = 0.2) +

# add points for CT deployment locations in LU21
tm_shape(cts_2022 %>%
           crop_shape(lus %>%
                        filter(namerefere == 'LU21'))) +
tm_dots(col = '#005AB5',
        size = 0.2) +

tm_shape(lus) +
tm_text('namerefere',
        size = 1.5,
        just = c(1.8, -2.15),
        fontface = 'bold') +

# add scale bar
tm_scale_bar(position = c('center', 'BOTTOM'),
             text.size = 1.2,
             width = 0.3) +

# add north arrow
tm_compass(type = 'arrow',
           position = c(0.01, 0.88),
           show.labels = 0,
           size = 5) +
```

```
  # specify layout elements
  tm_layout(frame = FALSE,
            bg.color = 'transparent')

# run lus_cts to see map
lus_cts
```

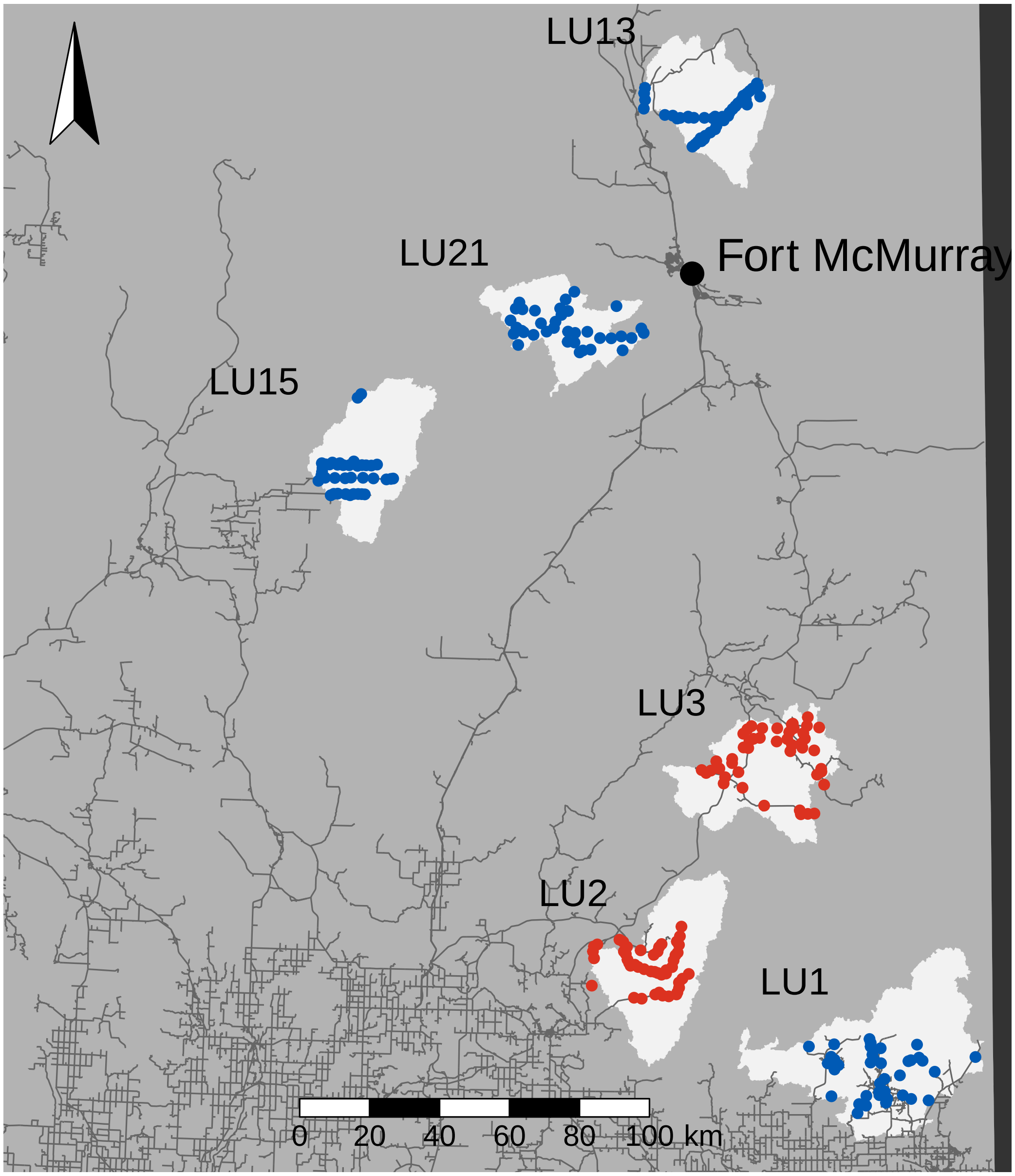

save `lus_cts` to 'figures' folder:

```r
tmap_save(lus_cts,
          'figures/study_area.png',
          width = 200,
          height = 250,
          units = 'mm',
          bg = 'transparent')
```

set NE Alberta bbox as an sf object:

```r
ne_ab_bbox <-
  ne_ab_bbox %>%
  st_as_sfc()
```

overlay map of Canada with Alberta highlighted with box around NE Alberta:

```r
ne_ab_can <-
  tm_shape(provs) +
  tm_fill(col = 'grey20') +

  tm_shape(ab_boundary) +
  tm_fill(col = 'grey70') +

  tm_shape(ne_ab_bbox) +
  tm_borders(col = 'black',
             lwd = 5) +

  tm_layout(frame = FALSE,
            bg.color = 'transparent')

# run ne_ab_can to see map
ne_ab_can
```

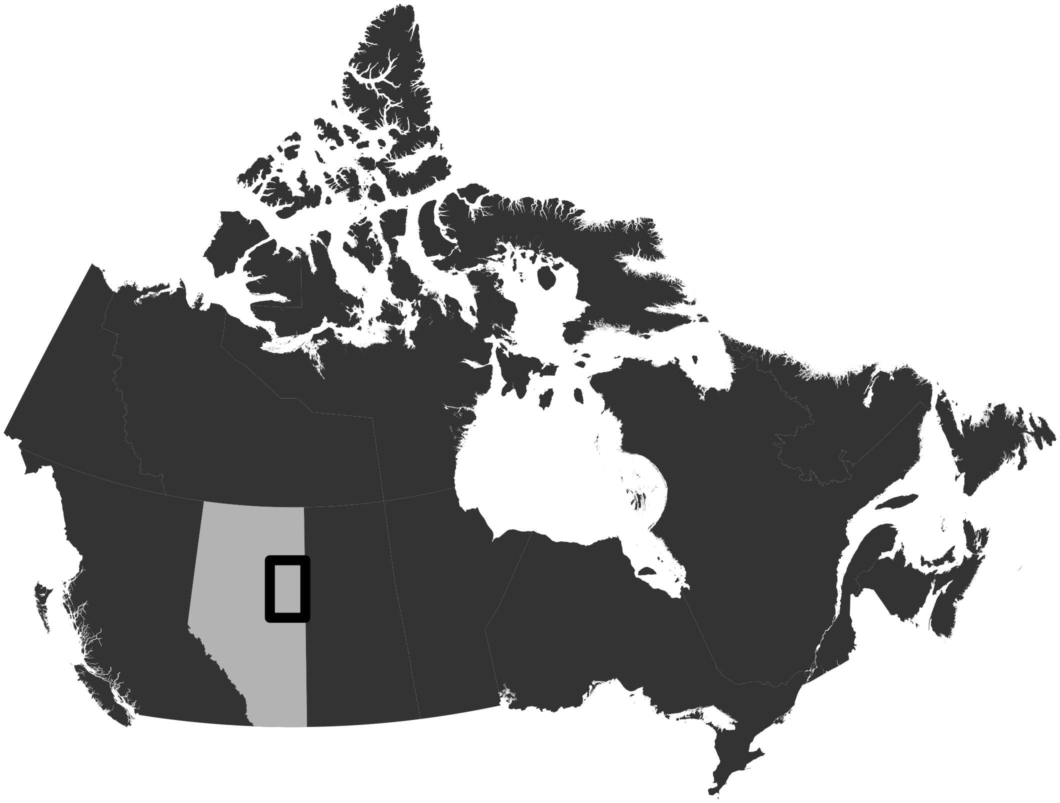

save `ne_ab_can` to ‘figures’ folder:

```
tmap_save(ne_ab_can,
          'figures/study_area_box.png',
          width = 250,
          height = 200,
          units = 'mm',
          bg = 'transparent')
```
