# Supplementary material for: Energy Infrastructure Clears the Way for Coyotes in Alberta's Oil Sands
Source: Ecol Evol. 2025 Aug 18;15(8):e71904. doi: 10.1002/ece3.71904 (PMC12360962; doi:10.1002/ece3.71904)
Supplement: Supplementary file 1 — Appendix S1. [file ECE3-15-e71904-s001.zip › coyote_formatting.pdf]

coyote_formatting

Jamie Clarke

last updated: 26/03/2025

about

This is a data formatting code walk-through for the manuscript *Energy infrastructure clears the way for coyotes in Alberta's oil sands*. It was written by Jamie F Clarke with help from Marissa A Dyck, and based on preliminary scripts by Larissa Bron. The data required to run the script is available on Jamie's GitHub. Happy formatting :-)

set-up

start by loading in relevant packages:

```
library(tidyverse)
library(PerformanceAnalytics)
library(purrr)
```

data import

load in percent cover of human/vegetation features:

```
landcover <-

  read_csv('data/raw/OSM_covariates_grouped_2021_2022.csv') %>%

  # select relevant features
  select(array,
         site,
         buff_dist,
```

```
        pipeline,
        roads,
        seismic_lines,
        seismic_lines_3D,
        trails,
        transmission_lines,
        lc_grassland,
        lc_coniferous,
        lc_broadleaf,
        lc_mixed,
        lc_shrub) %>%

  # combine natural landcover types into nat_land (i.e., natural landcover)
  mutate(nat_land =
           lc_grassland +
           lc_coniferous +
           lc_broadleaf +
           lc_mixed +
           lc_shrub)
```

load in proportional detections of coyotes:

```
coyote_det <-

  read_csv('data/raw/OSM_proportional_detections_merged_2021_2022.csv') %>%

  # rename coyote presence and absence for clarity
  rename(coyote_pres = coyote, # new variable name = coyote_pres
         coyote_abs = absent_coyote) %>% # new variable name = coyote_abs

  # select site (LU #), coyote_pres and coyote_abs
  select(site,
         coyote_pres,
         coyote_abs)
```

load in total detections of other mammal species and join the 2021-2022 + 2022-2023 datasets using `purrr`:

```
mammal_det <-

  # provide file path
  file.path('data/raw',
            c('OSM_total_detections_2021.csv',
              'OSM_2022_total_detections.csv')) %>%
```

```
            # use purr map_dfr to read in files and merge them
            map_dfr(
              ~.x %>%
                read_csv(.,
                         col_types = cols(site = col_factor(),
                                          .default = col_integer()))) %>%
  # reformat column names
  set_names(
    names(.) %>%
      tolower() %>% # change letters to lower case
      gsub(" ", "_", .) %>% # substitute spaces for underscores
      gsub("-", "_", .)) %>% # substitute dashes for underscores

  # rename coyote column as coyote_tot to easily differentiate between proportional co
  rename(coyote_tot = coyote) %>%

  # select relevant mammal species
  select(site,
         coyote_tot,
         fisher,
         snowshoe_hare,
         white_tailed_deer,
         cougar,
         lynx,
         red_squirrel,
         moose,
         grey_wolf,
         caribou)
```

join dataframes together into `coyote_data` master dataframe and clean it up:

```
coyote_data <-

landcover %>%

  left_join(coyote_det,
            by = 'site') %>%

  left_join(mammal_det,
            by = 'site') %>%

  # filter this dataset for buffer distance of 4750 m
  filter(buff_dist == 4750) %>%
```

```
  # remove columns not needed
  select(-c(buff_dist,
            lc_grassland,
            lc_coniferous,
            lc_broadleaf,
            lc_mixed,
            lc_shrub))
```

remove old dataframes from the environment to keep things organized:

```
rm(coyote_det,
   landcover,
   mammal_det)
```

data exploration

check histograms for each covariate of interest - looking for very left-skewed, zero-inflated plots that indicate little presence on the lansdscape (for modelling):

```
hist(coyote_data$nat_land)
```

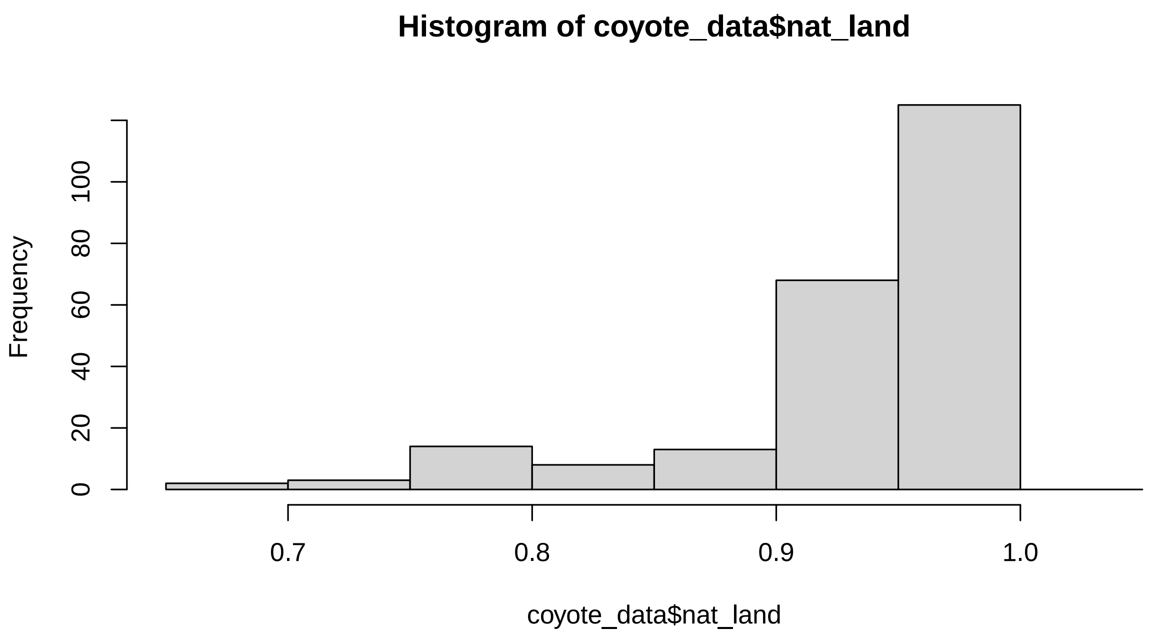

```r
hist(coyote_data$pipeline)
```

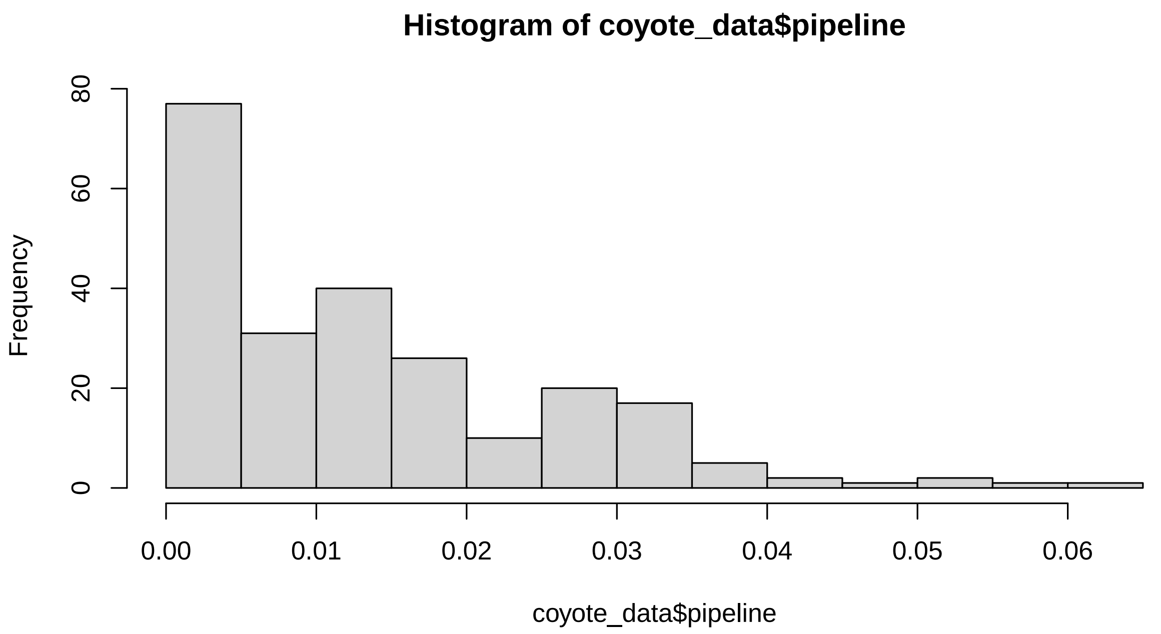


```r
hist(coyote_data$roads)
```

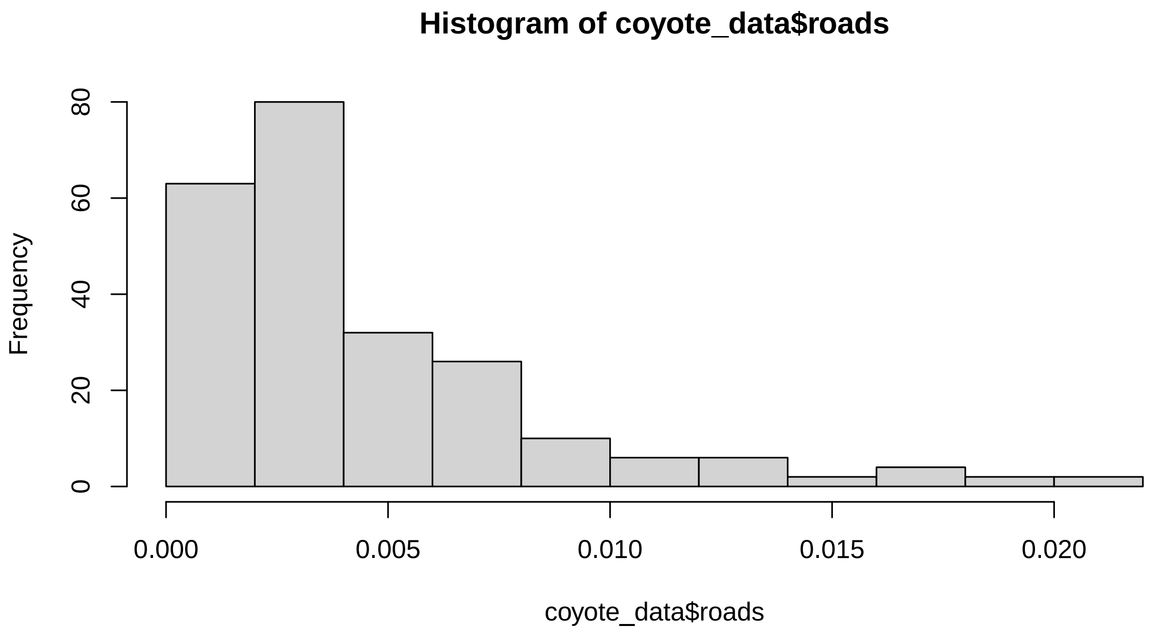

```r
hist(coyote_data$seismic_lines)
```

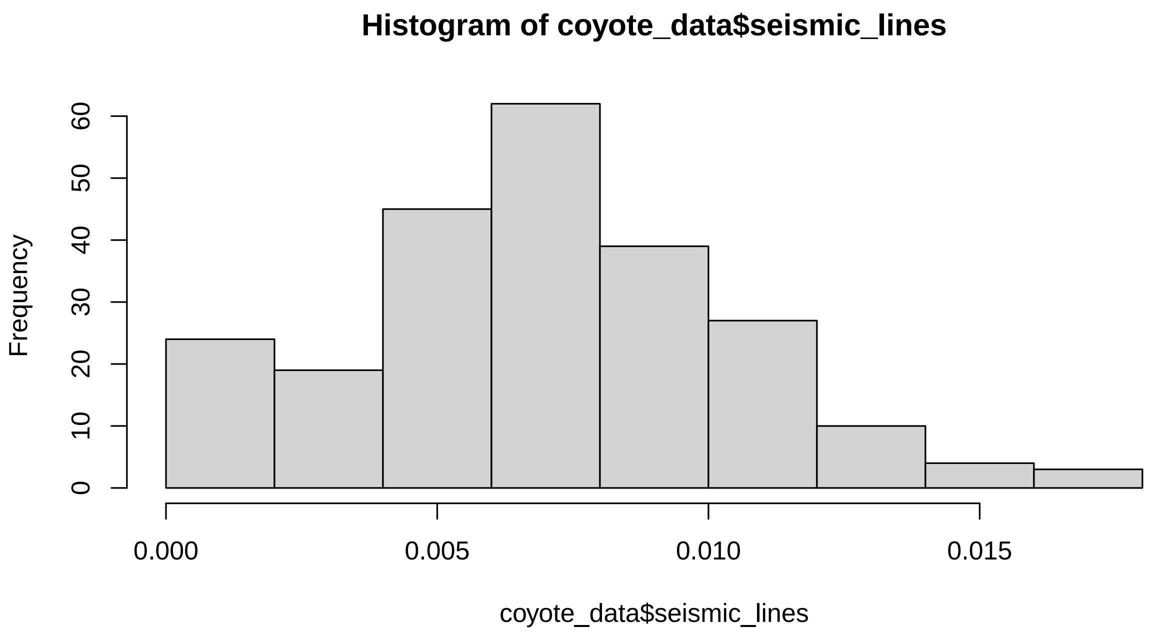


```r
hist(coyote_data$seismic_lines_3D)
```

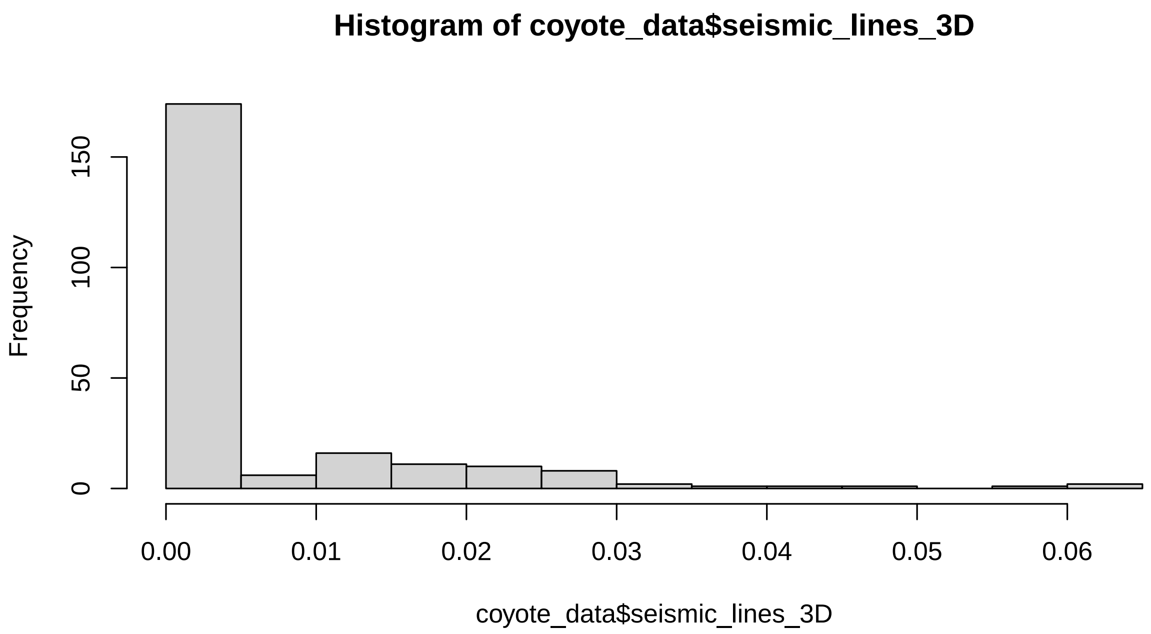

```r
hist(coyote_data$trails)
```

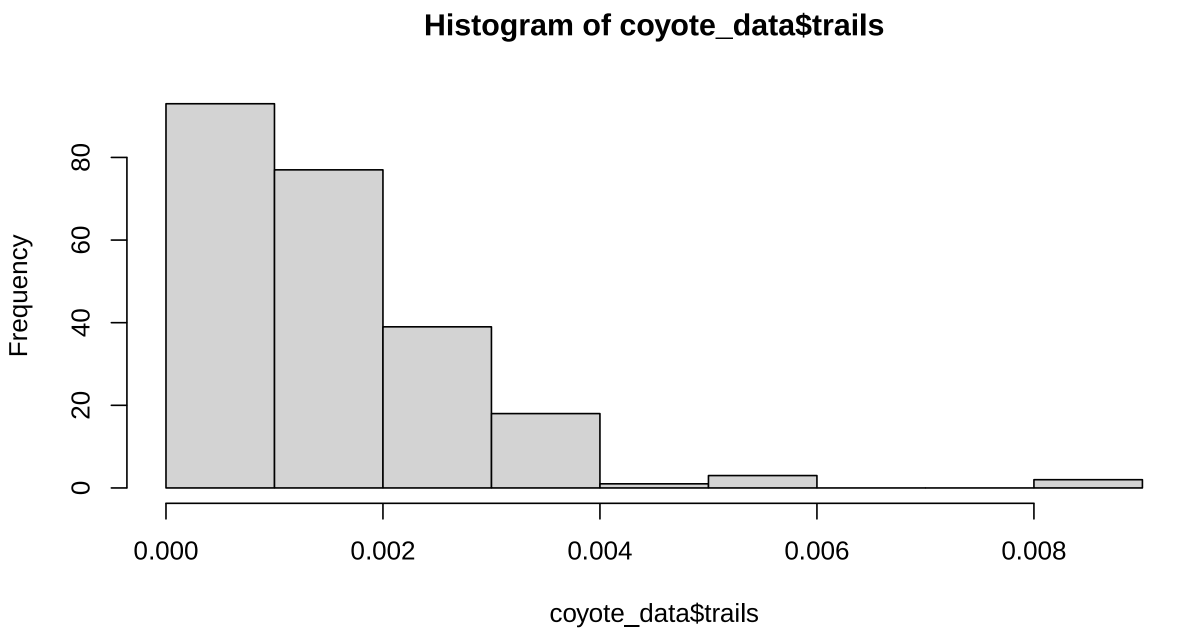


```r
hist(coyote_data$transmission_lines)
```

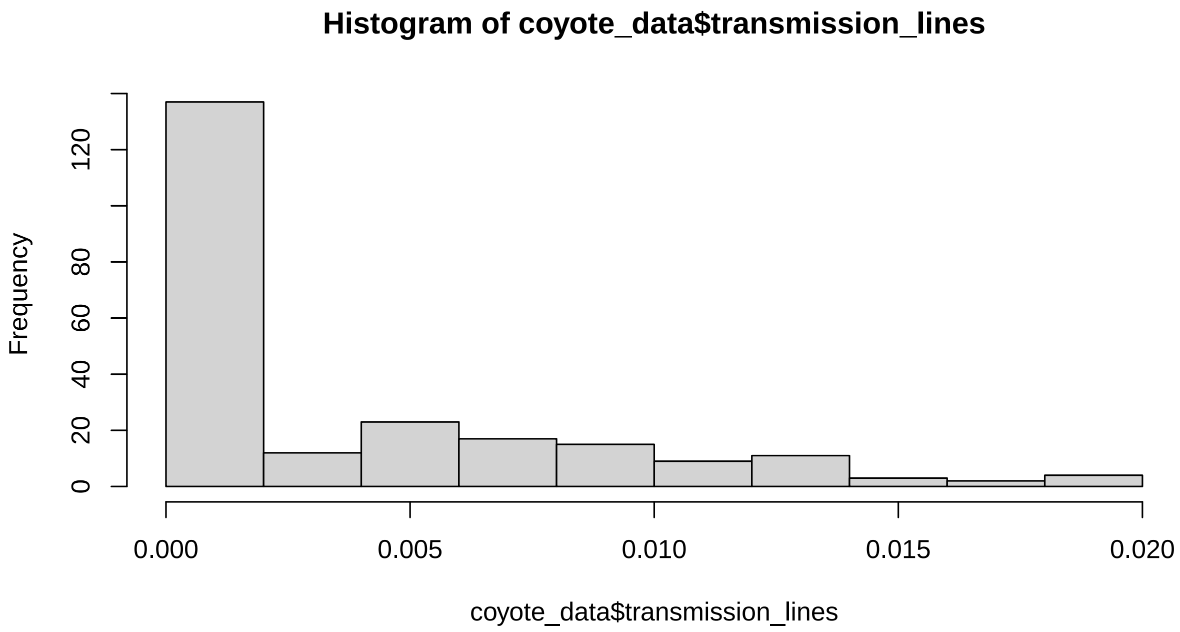

```r
hist(coyote_data$fisher)
```

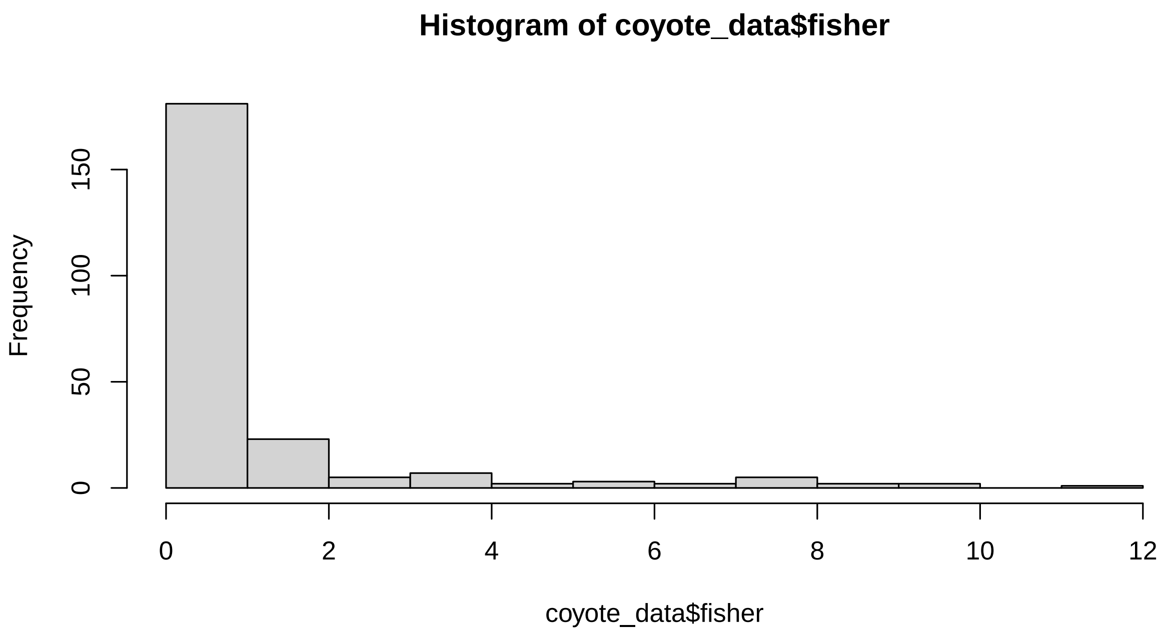


```r
hist(coyote_data$snowshoe_hare)
```

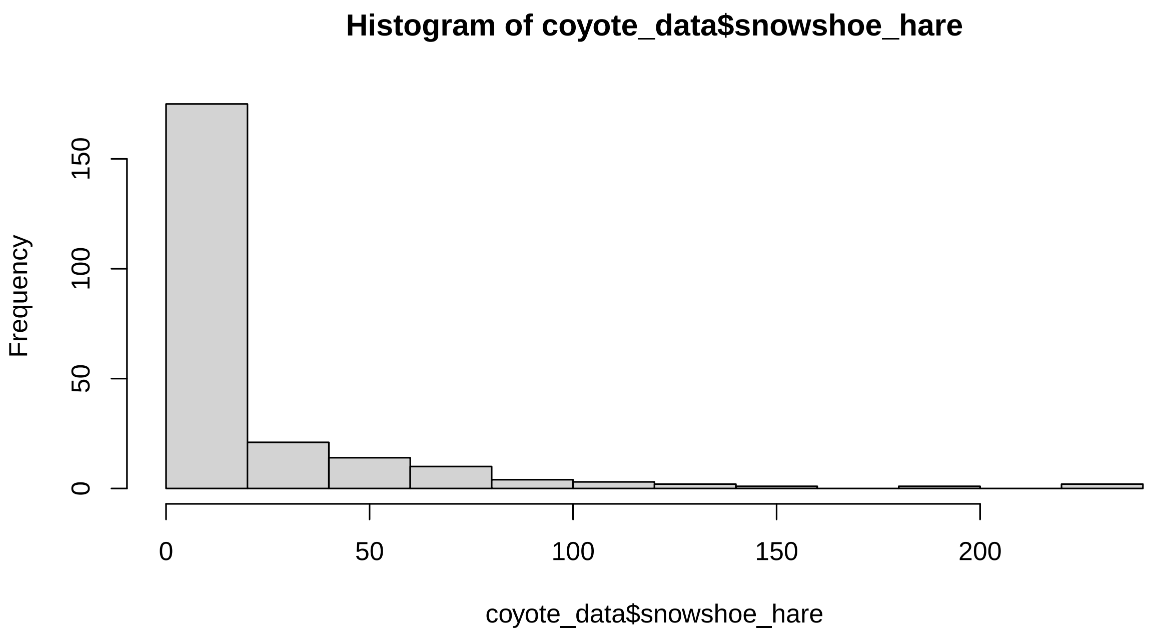

```
hist(coyote_data$white_tailed_deer)
```

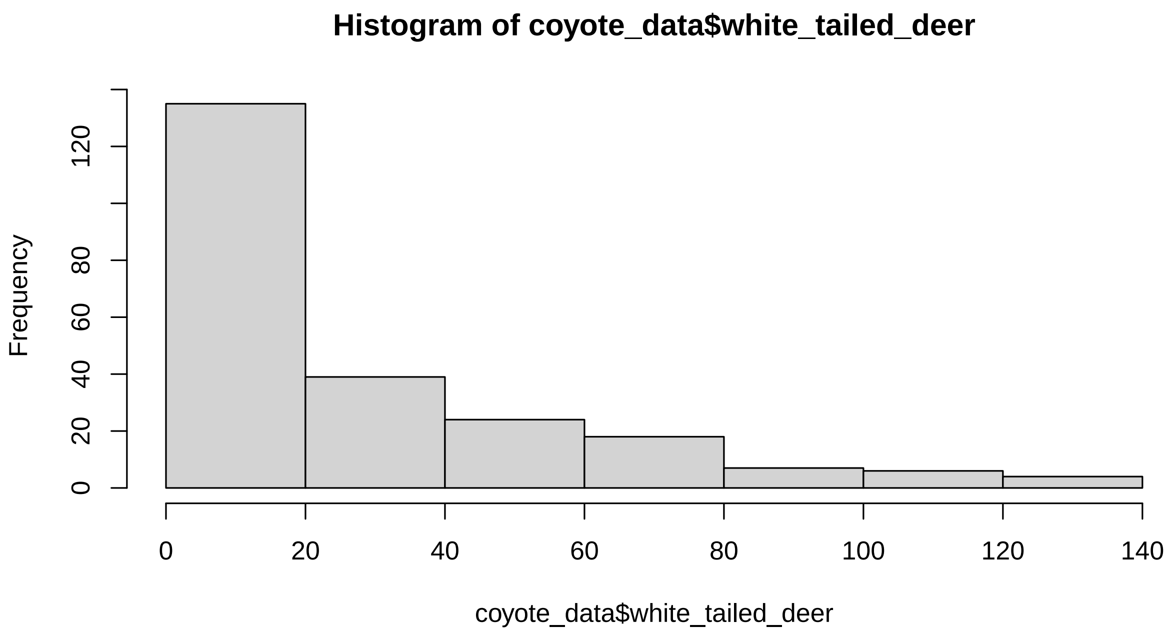


```
hist(coyote_data$cougar)
```

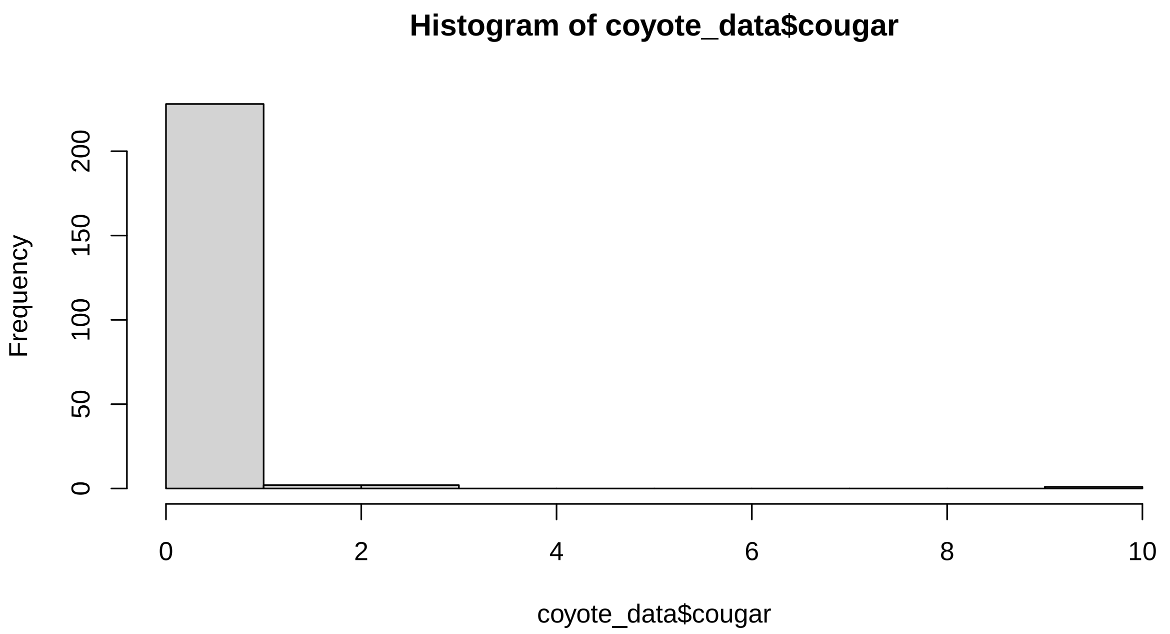

```r
hist(coyote_data$lynx)
```

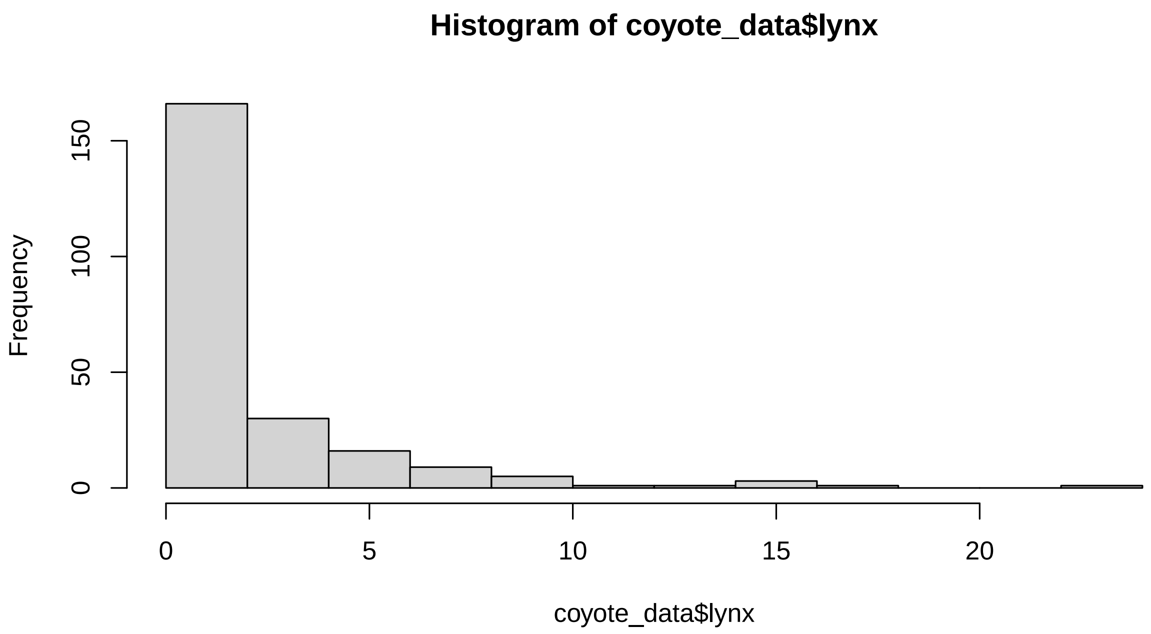


```r
hist(coyote_data$red_squirrel)
```

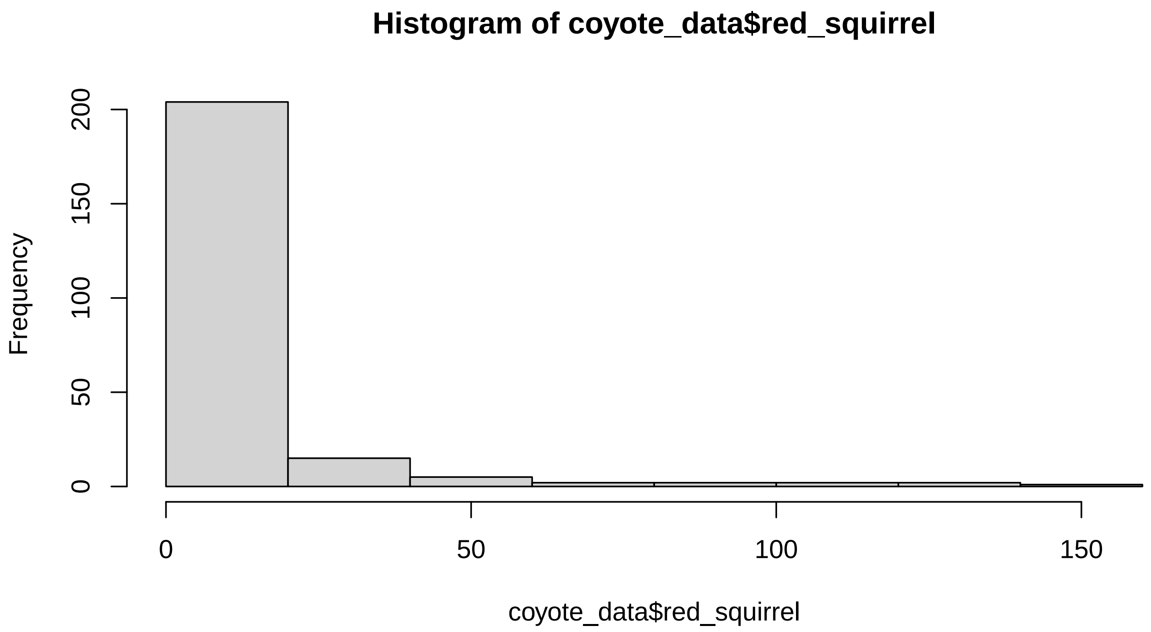

```r
hist(coyote_data$moose)
```

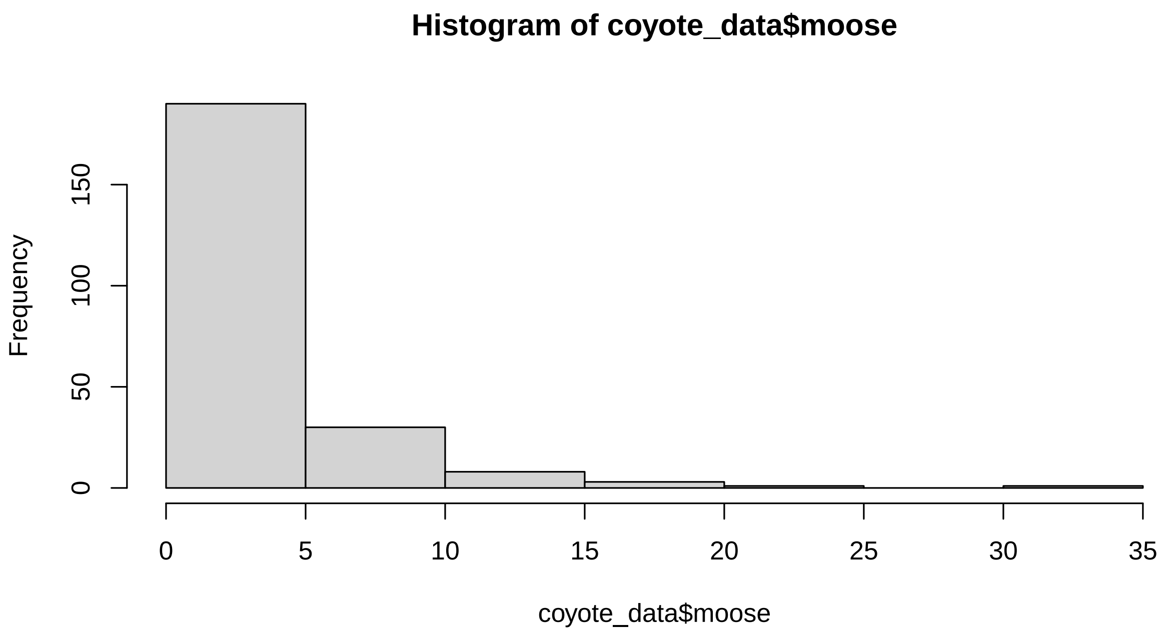


```r
hist(coyote_data$grey_wolf)
```

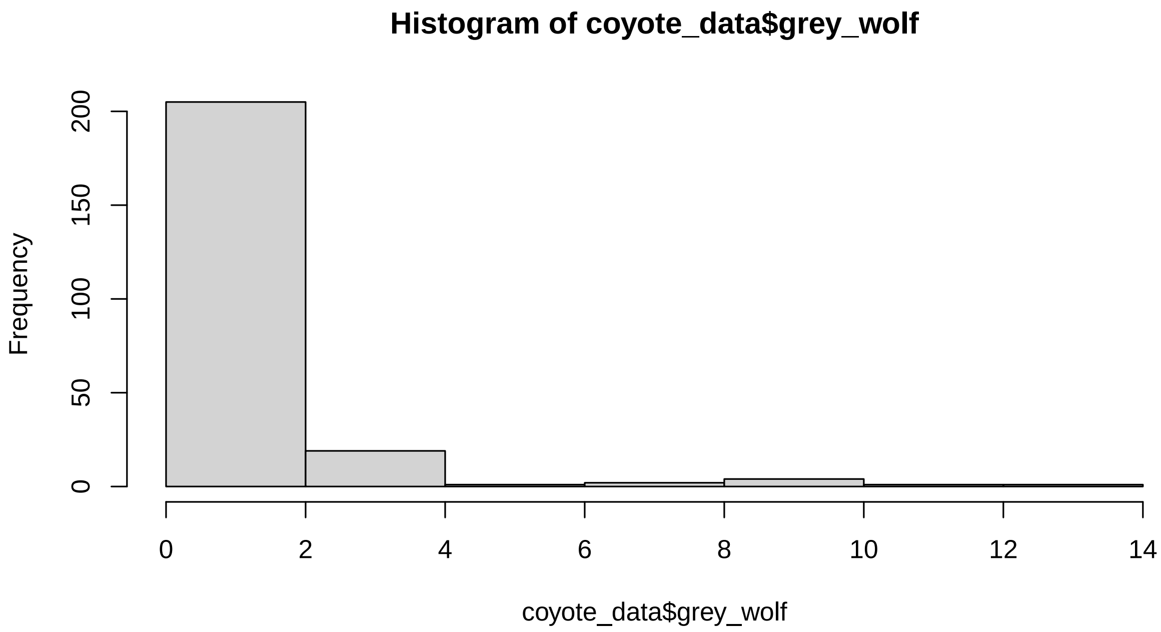

```
hist(coyote_data$caribou)
```

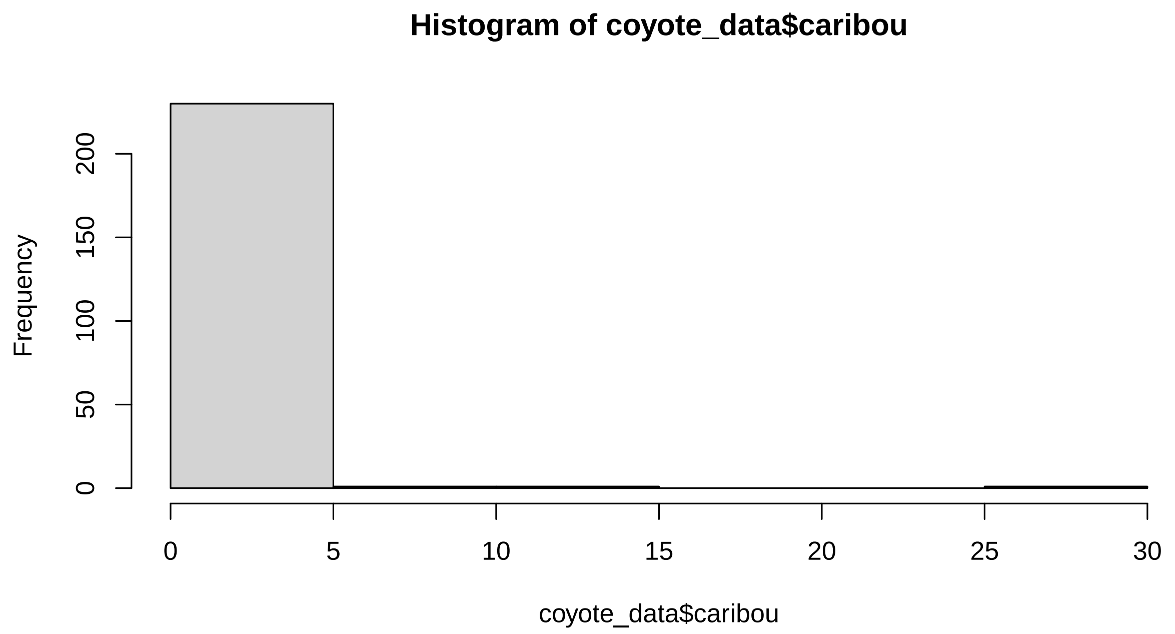

the plots above show us that there are too few detections of cougars and caribou to take further into analyses - so remove cougars and caribou from the dataframe:

```
coyote_data <-

  coyote_data %>%

  select(-c(cougar,
            caribou))
```

save formatted project dataframe

save `coyote_data` as .csv in 'processed' data folder:

```
write_csv(coyote_data,
          'data/processed/coyote_data.csv')
```

test for correlation

make a new variable of all the covariates to consider:

```r
coyote_cor <-

  coyote_data %>%

  select(pipeline,
         roads,
         seismic_lines,
         seismic_lines_3D,
         trails,
         transmission_lines,
         nat_land,
         fisher,
         snowshoe_hare,
         white_tailed_deer,
         lynx,
         red_squirrel,
         moose,
         grey_wolf)
```

side note: the text size in the correlation plots is really tiny… to change that, run the following line of code and, in the pop-up window, on line 17, change `cex` to 5 (or whatever you want):

```r
trace("chart.Correlation",
      edit = T)
```

graph a Pearson's correlation matrix:

```r
chart.Correlation(coyote_cor,
                  histogram = TRUE,
                  method = 'pearson')
```

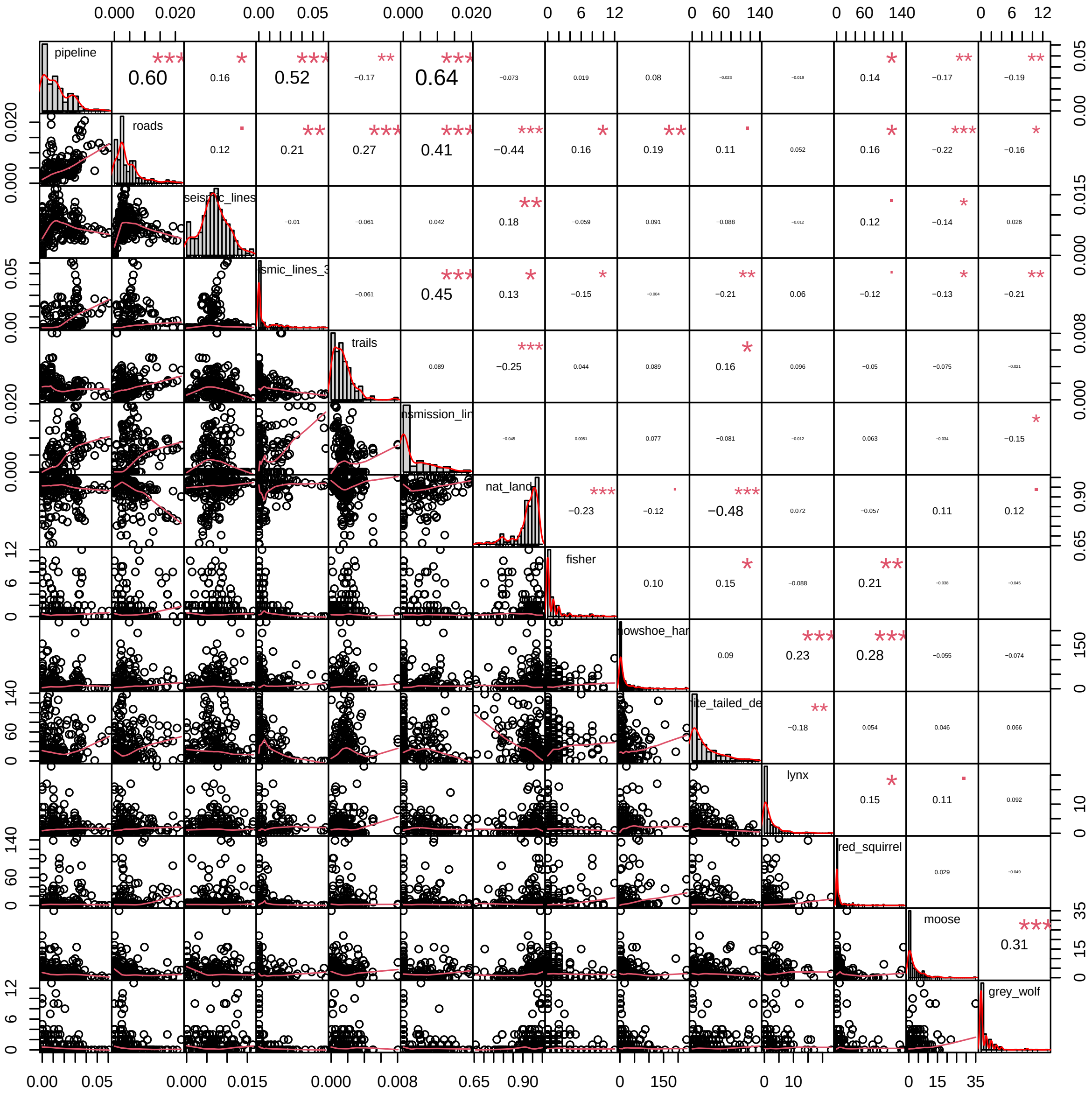

results:
- transmission lines and pipelines = 0.64
- roads and pipelines = 0.60

graph a Spearman's correlation matrix:

```
chart.Correlation(coyote_cor,
                  histogram = TRUE,
                  method = 'spearman')
```

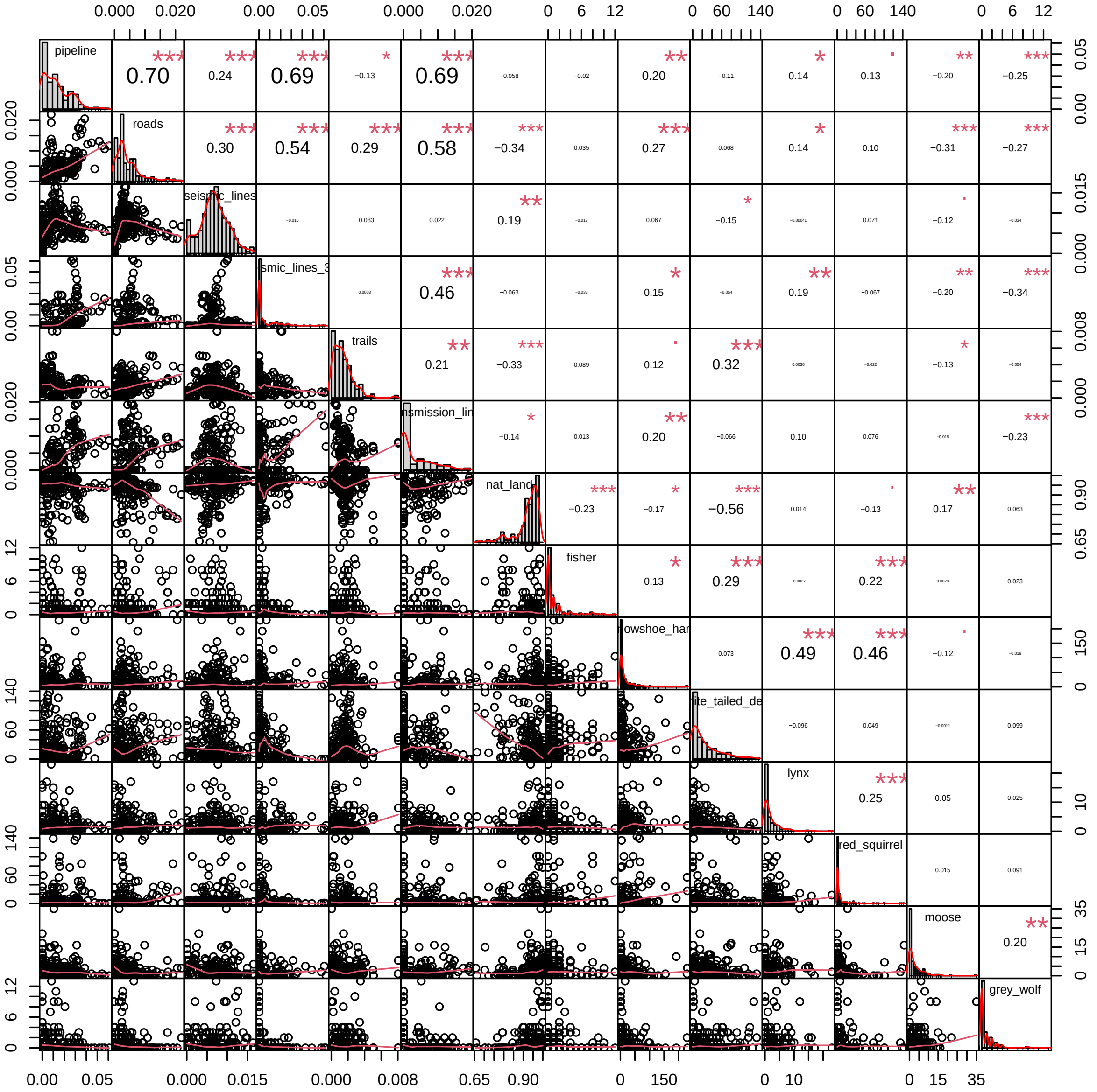

results:
- roads and pipelines = 0.70
- transmission lines and pipelines = 0.69
- 3D seismic lines and pipelines: 0.69
