# Supplementary material for: Energy Infrastructure Clears the Way for Coyotes in Alberta's Oil Sands
Source: Ecol Evol. 2025 Aug 18;15(8):e71904. doi: 10.1002/ece3.71904 (PMC12360962; doi:10.1002/ece3.71904)
Supplement: Supplementary file 1 — Appendix S1. [file ECE3-15-e71904-s001.zip › ece371904-sup-0001-AppendixS1.docx]

## Supplementary Information

**Table S1.** Description of linear feature types, adapted from Alberta Biodiversity Monitoring Institute (2021) and Dyck (in prep), with additional information from Stern et al. (2018).

| **linear feature** | **description** |
| --- | --- |
| pipelines | a line of underground and overground pipes of substantial length and capacity used for the conveyance of petrochemicals; the physical clearing that contains underground and above-ground high pressure pipelines |
| roads | non-vegetated, impermeable surfaces used for motorized vehicle or aircraft transportation or access |
| (conventional) seismic lines | cleared corridors created during hydrocarbon exploration; typically 5-10 m in width, many kilometres long and widely-spaced (200-500 m apart) |
| 3D seismic lines | cleared corridors created during hydrocarbon exploration; typically 1.5-3 m in width, in a cross-hatch pattern. |
| trails | cleared corridors surfaced with dirt or low vegetation for human and vehicle access |
| transmission lines | cleared corridors designated for the location of power transmission line infrastructure that are greater than 10 m wide |

**Table S2.** Beta coefficient table for the top-performing models from step 1. The wide linear feature model was the most supported, followed by the global linear feature model.

| **model** | **parameter** | **beta coefficient** | **standard error** | **Z value** | **Pr(>\|z\|)** |
| --- | --- | --- | --- | --- | --- |
| wide linear feature | roads | 0.586 | 0.059 | 9.867 | < 0.001 |
|  | seismic lines | 0.184 | 0.074 | 2.506 | 0.012 |
|  | transmission lines | 0.014 | 0.060 | 0.234 | 0.815 |
| global linear feature | roads | 0.564 | 0.061 | 9.219 | < 0.001 |
|  | seismic lines | 0.193 | 0.074 | 2.605 | 0.009 |
|  | 3D seismic lines | -0.064 | 0.069 | -0.927 | 0.354 |
|  | transmission lines | 0.053 | 0.066 | 0.794 | 0.427 |
|  | trails | 0.058 | 0.058 | 1.008 | 0.314 |

**Table S3.** Beta coefficient table for the top-performing models from step 2. The global model was the most supported, followed by the global model with interaction terms.

| **model** | **parameter** | **beta coefficient** | **standard error** | **Z value** | **Pr(>\|z\|)** |
| --- | --- | --- | --- | --- | --- |
| global | natural landcover | -0.404 | 0.055 | -7.396 | < 0.001 |
|  | wide linear features | 0.497 | 0.070 | 7.131 | < 0.001 |
|  | white-tailed deer | 0.064 | 0.065 | 0.982 | 0.326 |
|  | moose | -0.063 | 0.057 | -1.105 | 0.269 |
|  | red squirrel | 0.081 | 0.047 | 1.725 | 0.084 |
|  | snowshoe hare | 0.188 | 0.048 | 3.948 | < 0.001 |
|  | grey wolf | 0.190 | 0.051 | 3.747 | < 0.001 |
|  | lynx | 0.167 | 0.050 | 3.370 | < 0.001 |
|  | fisher | 0.018 | 0.050 | 0.356 | 0.722 |
| global interaction | natural landcover | -0.416 | 0.055 | -7.502 | < 0.001 |
|  | white-tailed deer | 0.042 | 0.067 | 0.618 | 0.536 |
|  | moose | -0.061 | 0.059 | -1.045 | 0.296 |
|  | red squirrel | 0.077 | 0.047 | 1.642 | 0.101 |
|  | lynx | 0.155 | 0.051 | 3.027 | 0.002 |
|  | fisher | 0.006 | 0.051 | 0.120 | 0.904 |
|  | wide linear features | 0.489 | 0.072 | 6.752 | < 0.001 |
|  | snowshoe hare | 0.221 | 0.056 | 3.922 | < 0.001 |
|  | grey wolf | 0.197 | 0.051 | 3.853 | < 0.001 |
|  | wide linear features *  snowshoe hare | -0.050 | 0.045 | -1.110 | 0.267 |
|  | wide linear features *  grey wolf | -0.098 | 0.076 | -1.289 | 0.197 |


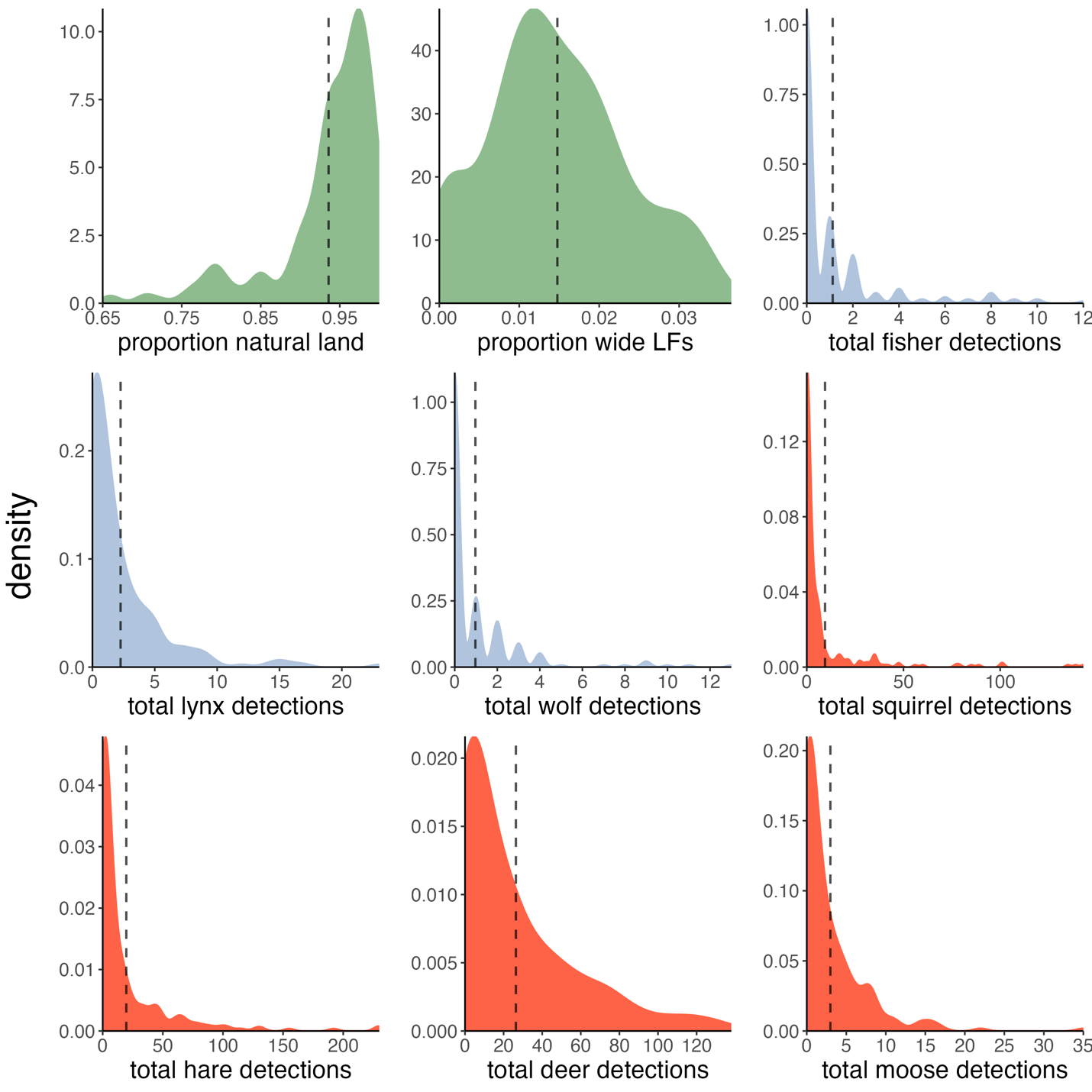


**Figure S1.** Distribution of covariates considered in step 2. Spread represents the proportion of landscape features within a 2,750 m buffer radius of a camera site or the total number of independent species detections at a camera site (n = 233). Dashed lines represent mean values for each covariate across all camera sites and LUs. Total independent species detections are zero-skewed given most camera sites will not detect all mammal species.


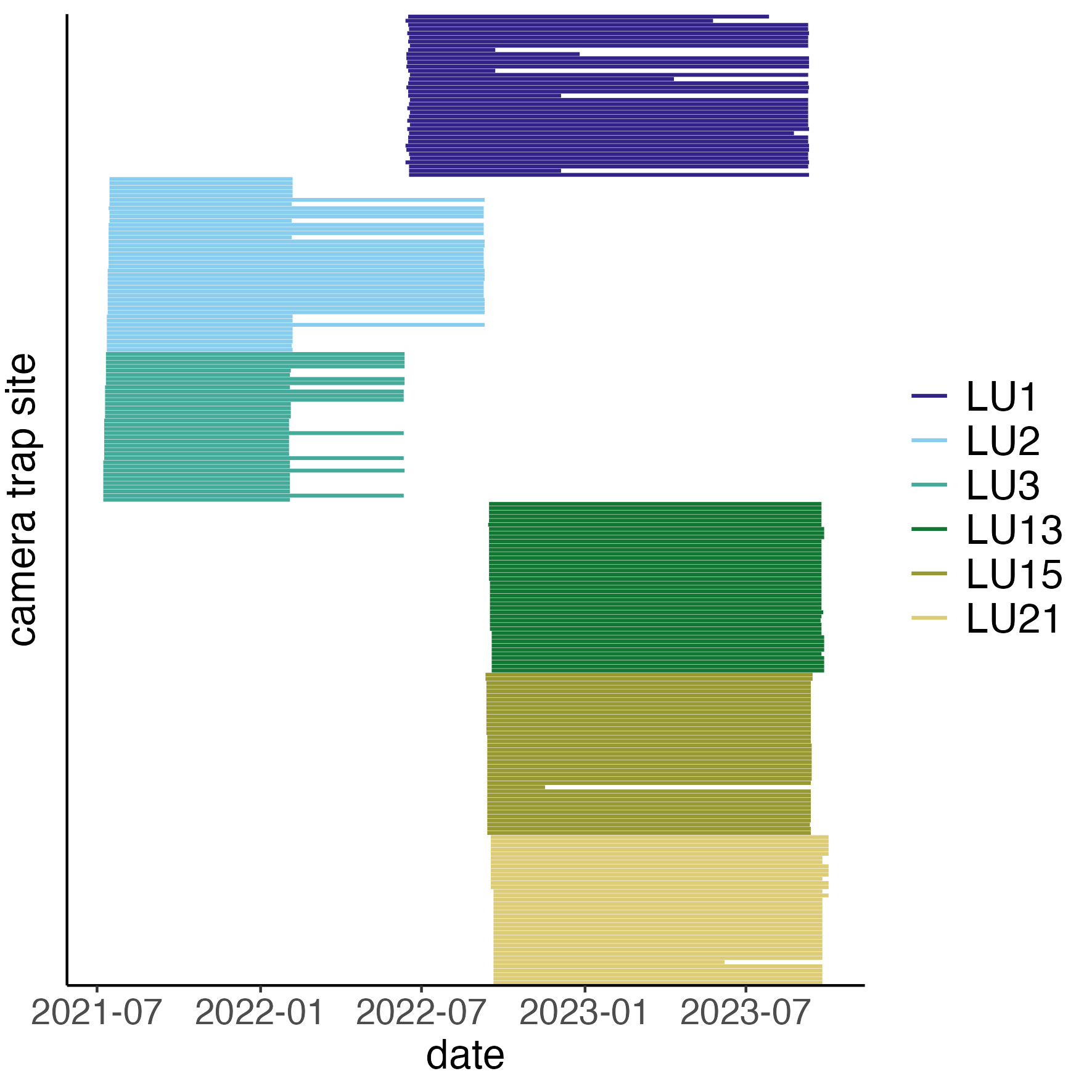


**Figure S2.** Camera trap operability plot, showing the date range each camera station (n = 233, coloured lines) were active across the study period.


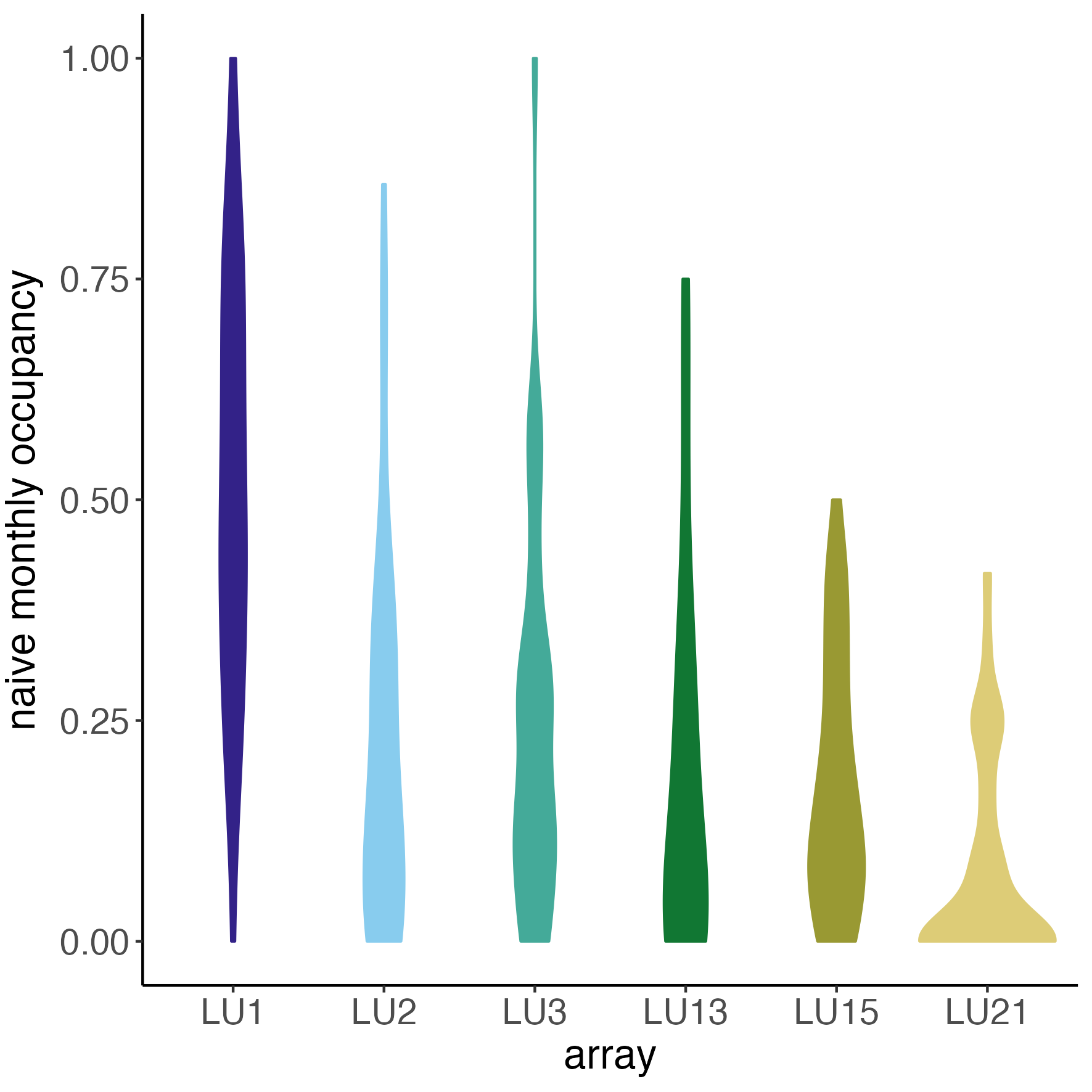


**Figure S3.** Naïve monthly coyote site occupancy (calculated as the number of months coyotes were present a camera site divided by the total number of months that camera site was active) across LUs. Note: the number of camera traps per landscape unit varies.


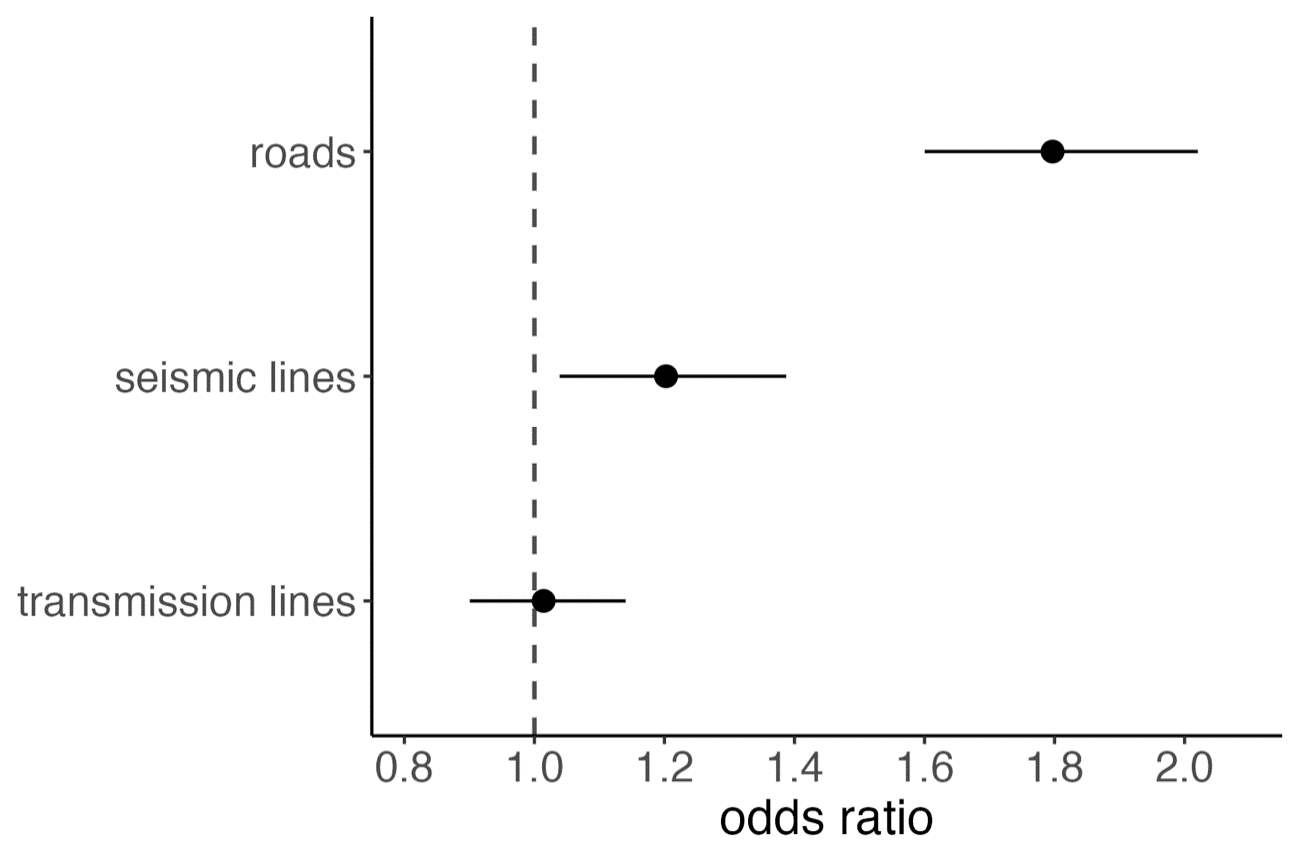


**Figure S4.** Odds ratio plot for the wide linear feature model – the best-supported model from step 1 – showing how the proportion of roads, seismic lines and transmission lines within a 4,750 m radius of a camera trap influence monthly coyote occurrence. Black dots represent exponentiated model coefficients; bars represent 95% confidence intervals.


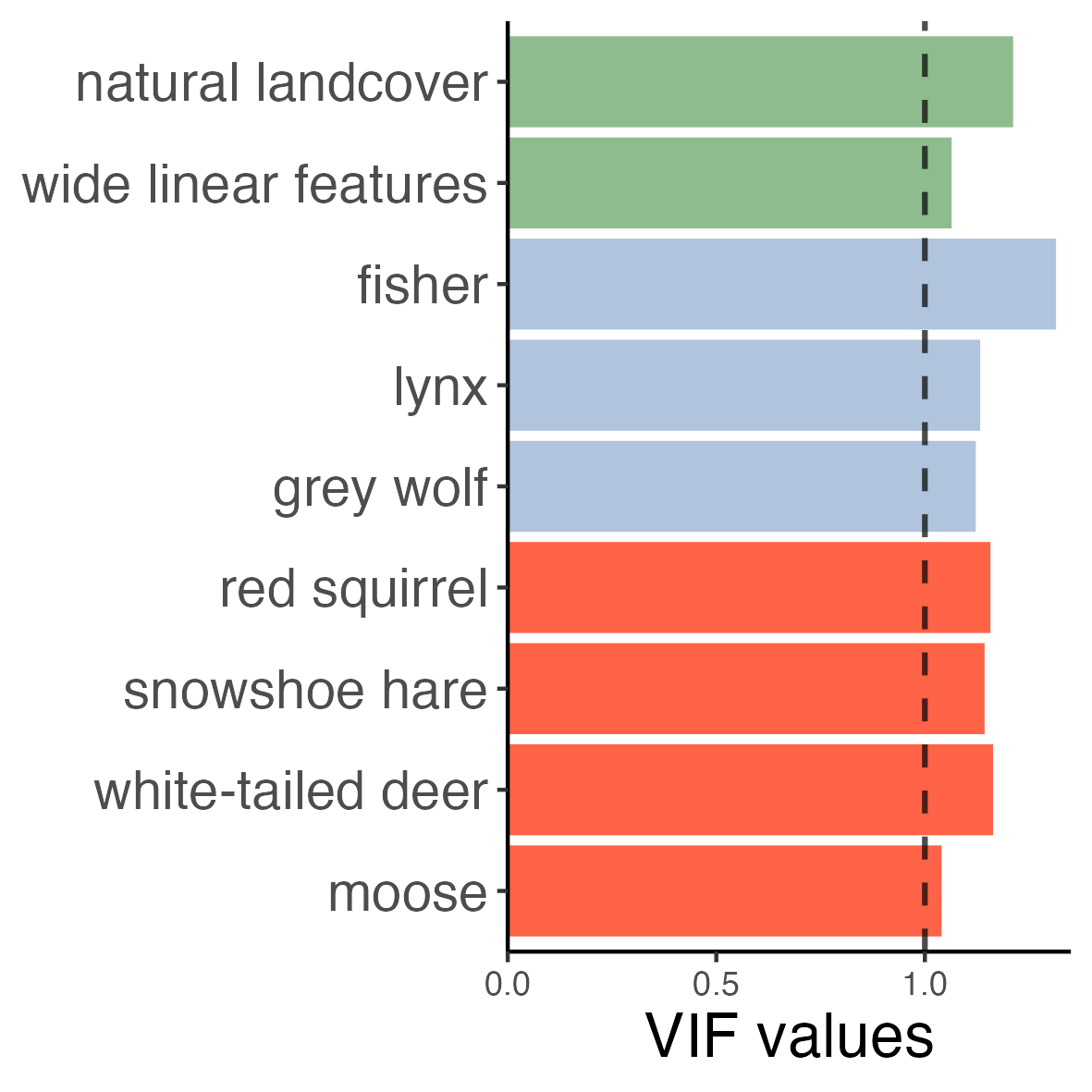


**Figure S5.** Variance inflation factors (VIFs) for global model covariates from step 2. A VIF value of 1 (dashed line) indicates no correlation, while larger numbers indicate more severe correlation. Bars are grouped into covariate categories: landscape features (green), competitor species (blues) and prey species (red).


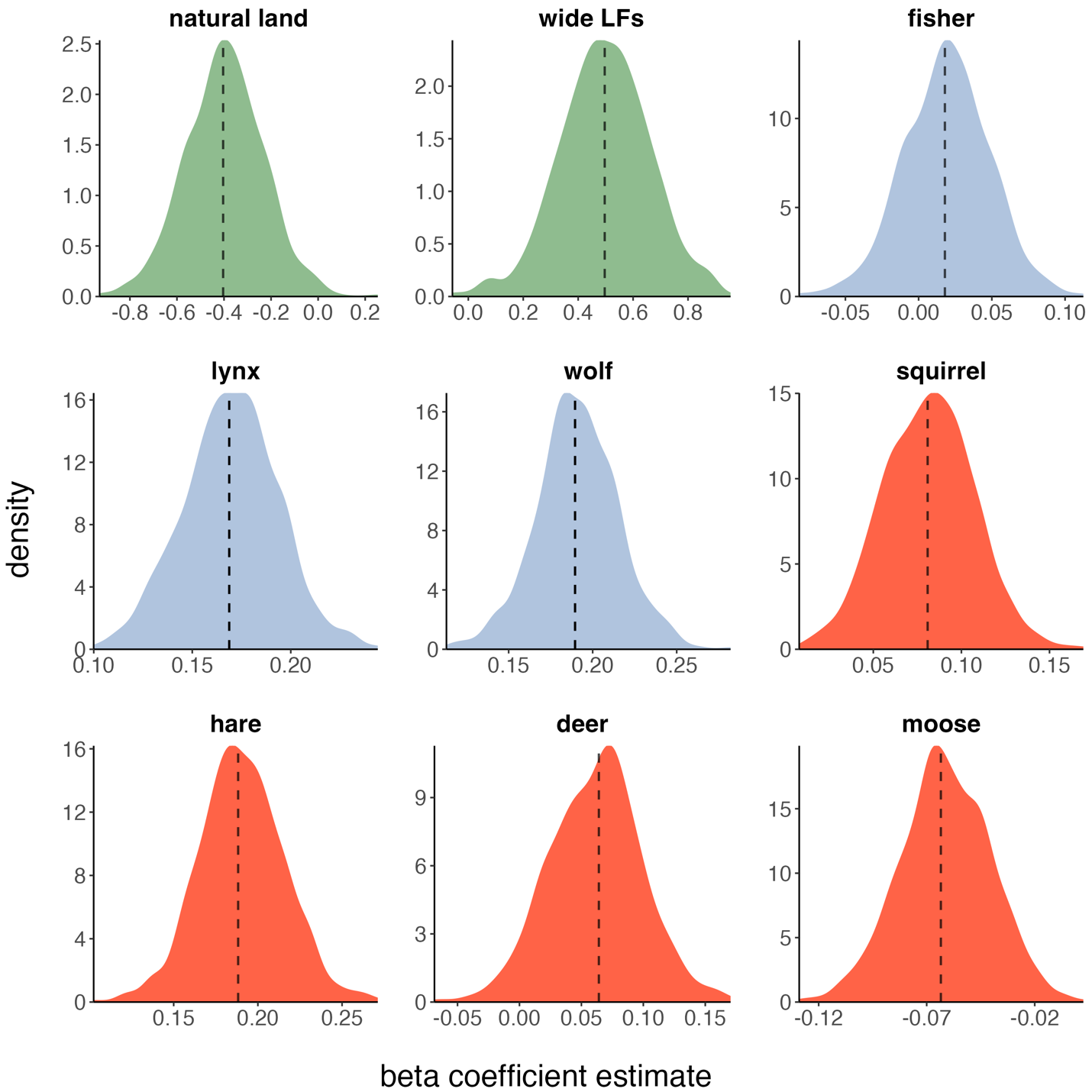

**Figure S6.** Spread of beta coefficient estimates from the simulated global model from step 2 after 1,000 iterations for landscape features (green), competitor species (blue) and prey species (red). Dashed lines represent “true” beta coefficient values from the global model. LFs is shorthand for linear features.


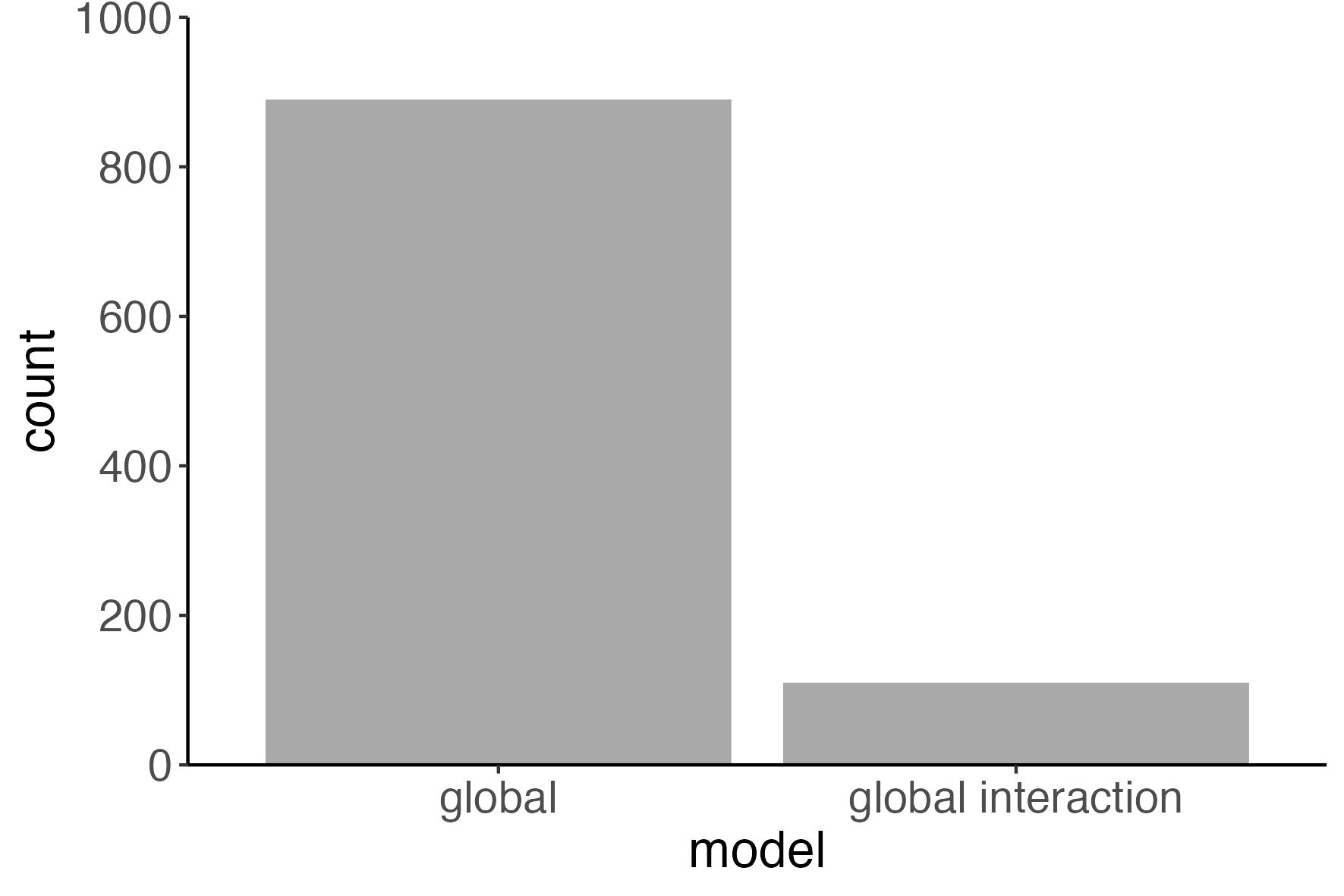


**Figure S7.** Best-supported models from simulated model selection (step 2). The global model – our top model – was best-supported in 89% of simulations. Our second best-performing model, the global interaction model, outranked the global model in 11% of simulations. No other models were top performing.
