# Supplementary figures and images for: Energy Infrastructure Clears the Way for Coyotes in Alberta's Oil Sands
Source: Ecol Evol. 2025 Aug 18;15(8):e71904. doi: 10.1002/ece3.71904 (PMC12360962; doi:10.1002/ece3.71904)

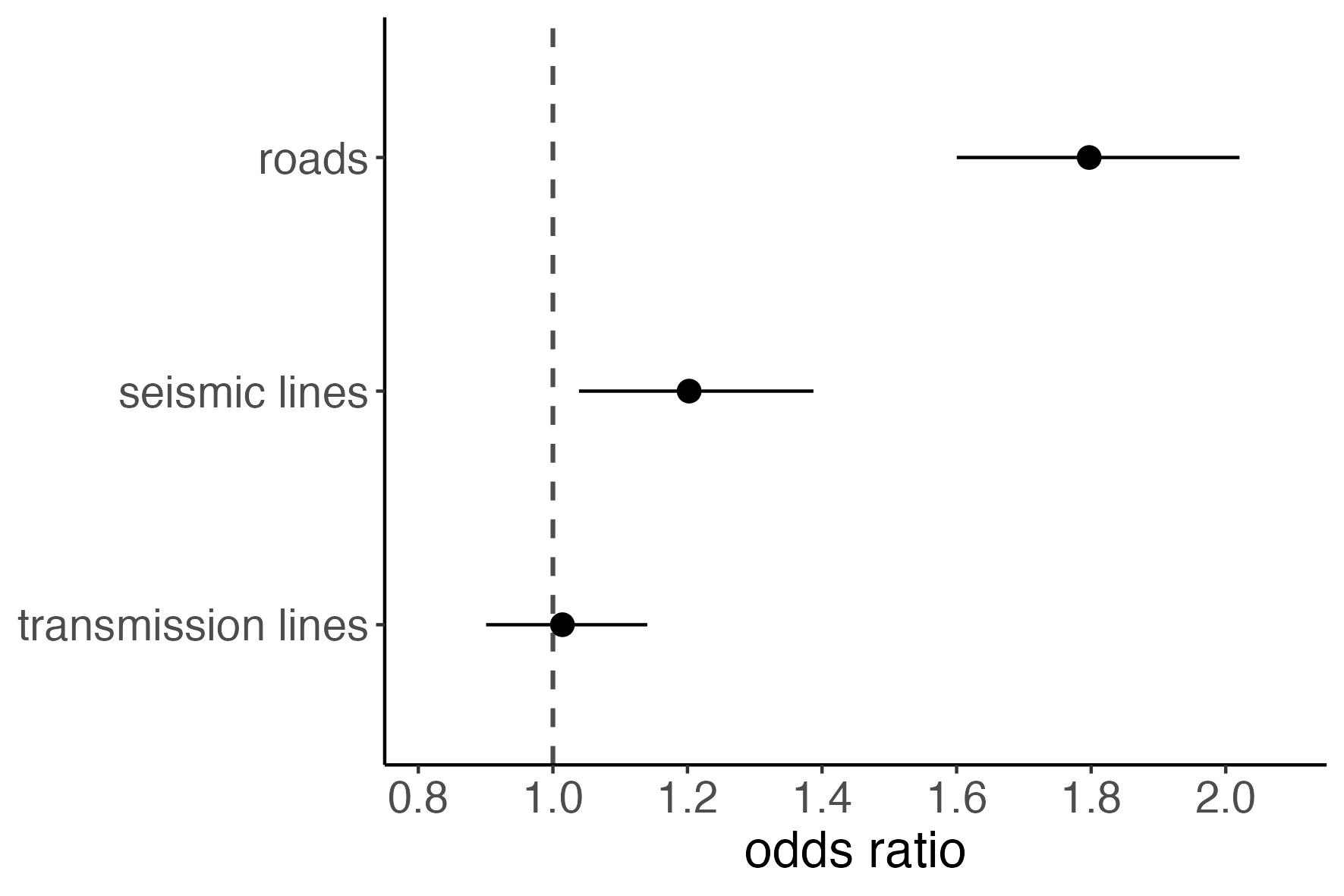

Supplement: Supplementary file 1 — Appendix S1. [file ECE3-15-e71904-s001.zip › sm_0004-FigS4.tiff]

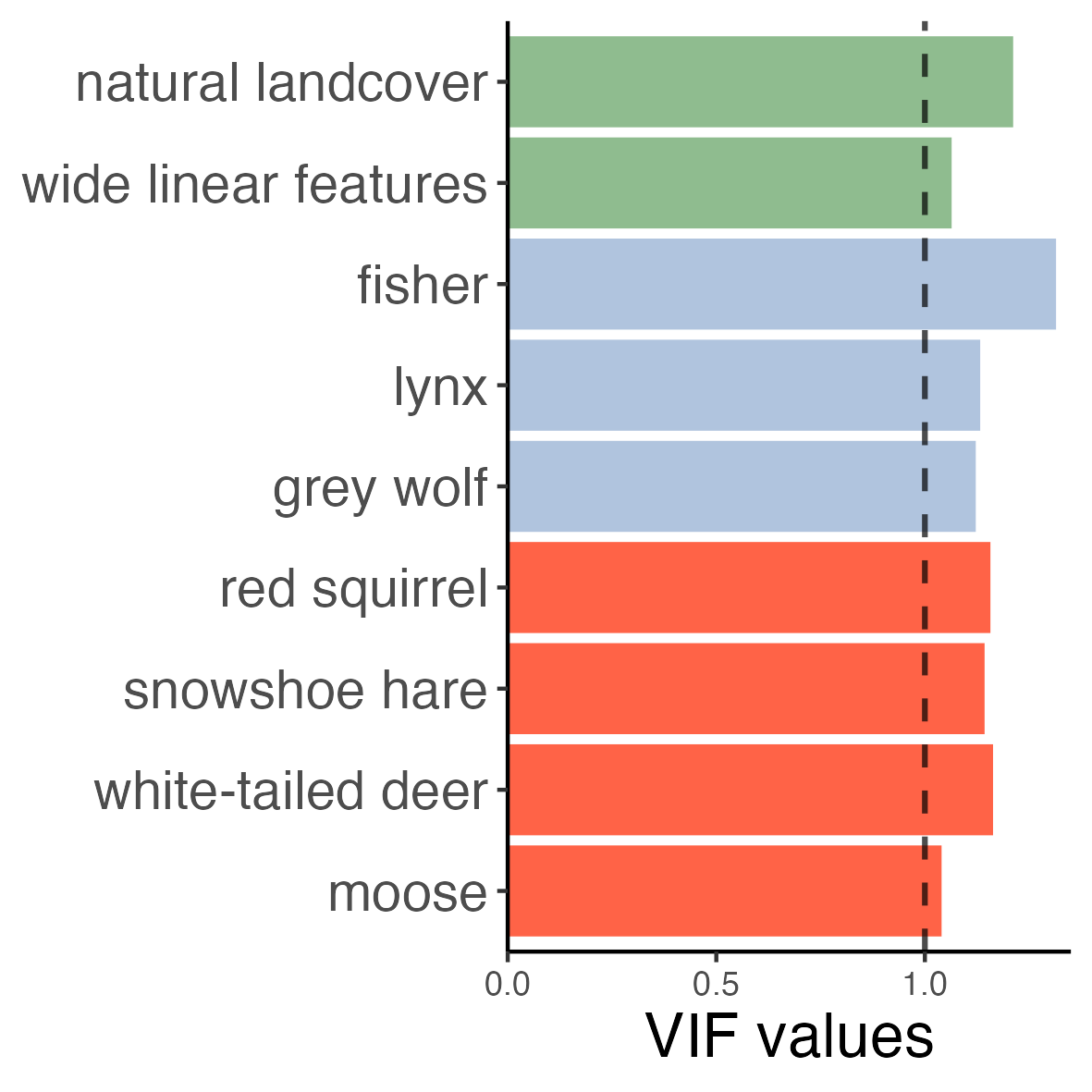

Supplement: Supplementary file 1 — Appendix S1. [file ECE3-15-e71904-s001.zip › sm_0005-FigS5.tiff]

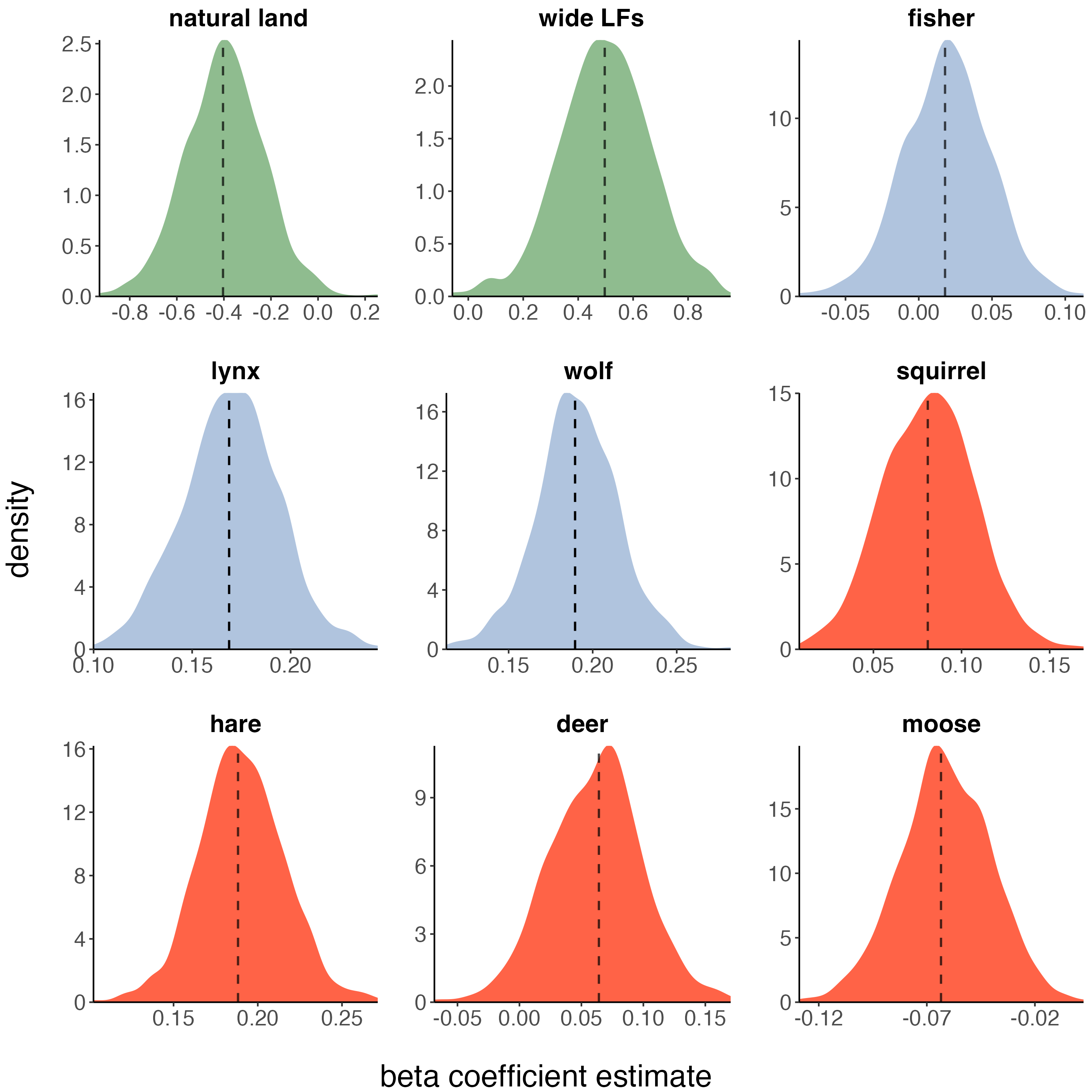

Supplement: Supplementary file 1 — Appendix S1. [file ECE3-15-e71904-s001.zip › sm_0006-FigS6.tiff]

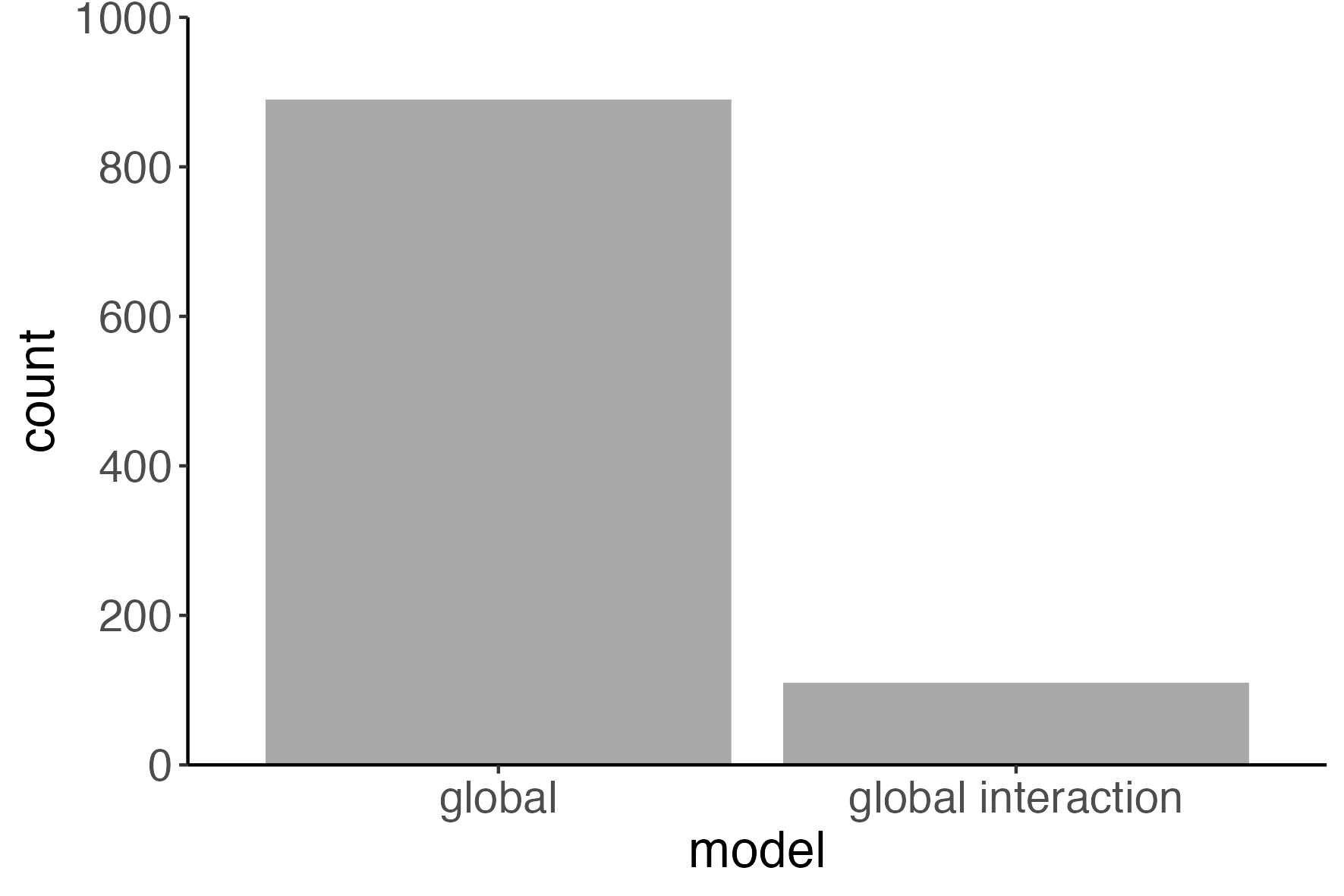

Supplement: Supplementary file 1 — Appendix S1. [file ECE3-15-e71904-s001.zip › sm_0007-FigS7.tiff]

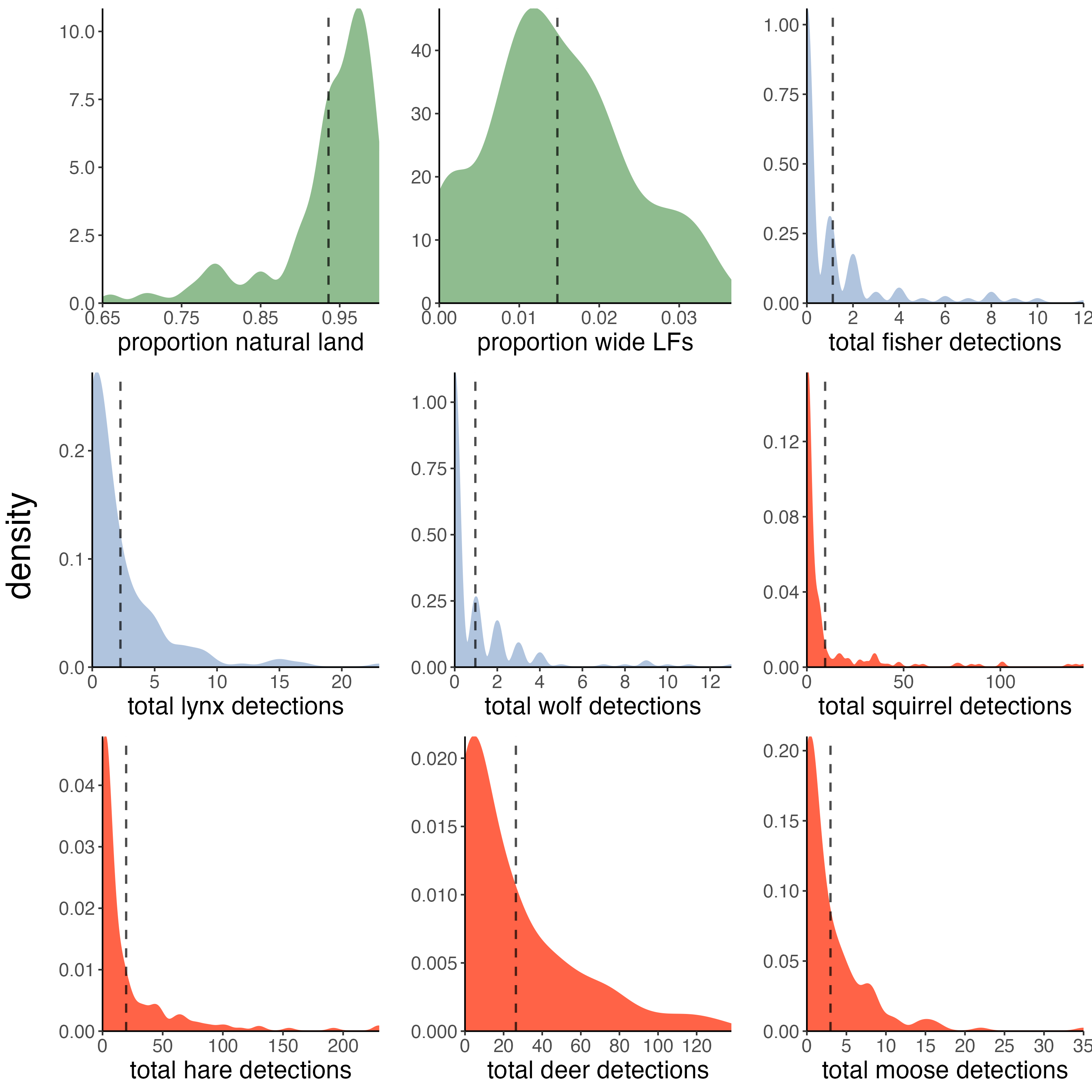

Supplement: Supplementary file 1 — Appendix S1. [file ECE3-15-e71904-s001.zip › sm_0001-FigS1.tiff]

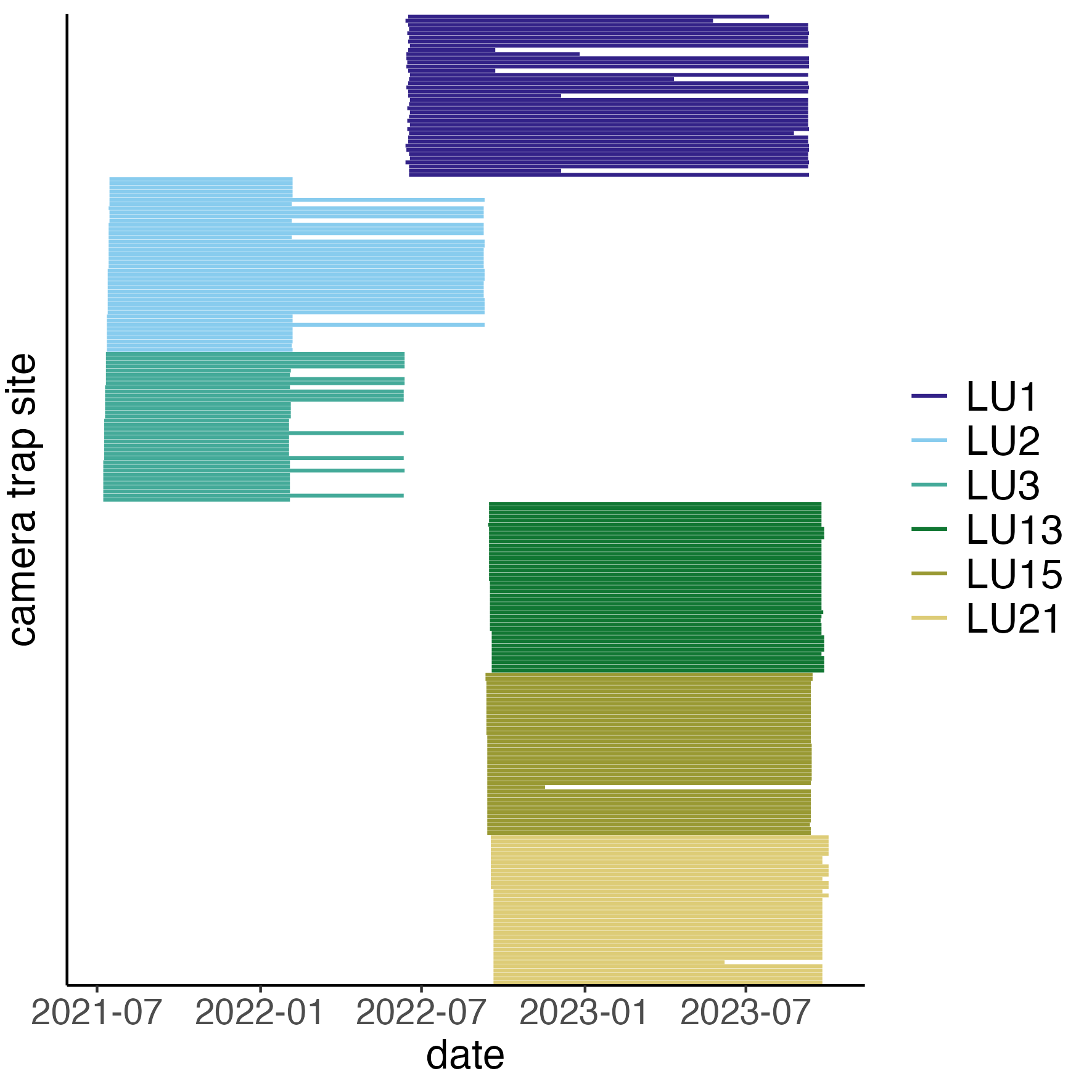

Supplement: Supplementary file 1 — Appendix S1. [file ECE3-15-e71904-s001.zip › sm_0002-FigS2.tiff]

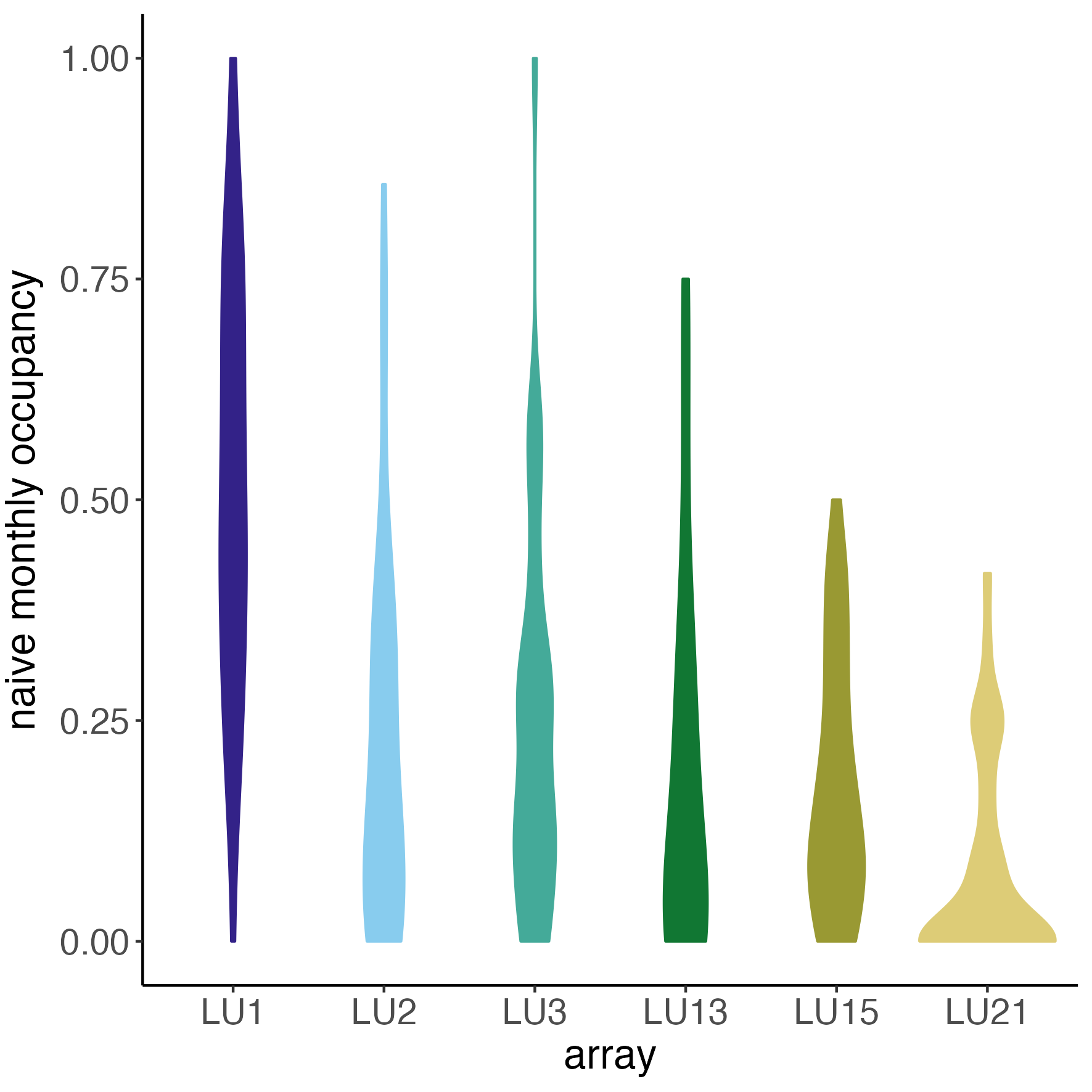

Supplement: Supplementary file 1 — Appendix S1. [file ECE3-15-e71904-s001.zip › sm_0003-FigS3.tiff]
